# The impact of cineole treatment timing on common cold duration and symptoms: Non-randomized exploratory clinical trial

**DOI:** 10.1371/journal.pone.0296482

**Published:** 2024-01-18

**Authors:** Andreas Michalsen, Kim Goldenstein, Peter Kardos, Ludger Klimek, Jürgen Palm, Dajana Parganlija, Johannes Stöckl

**Affiliations:** 1 Department of Internal, Integrative and Complementary Medicine, Immanuel Hospital Berlin, Berlin, Germany; 2 MCM Klosterfrau Vertriebsgesellschaft mbH, Klosterfrau Healthcare Group, Cologne, Germany; 3 Lung Centre Maingau, Frankfurt am Main, Germany; 4 Centre for Rhinology and Allergology, Wiesbaden, Germany; 5 ENT Practice, Röthenbach, Germany; 6 Signum | Brands GmbH, Cologne, Germany; 7 Institute of Immunology, Medical University of Vienna, Vienna, Austria; Srebrnjak Children’s Hospital, CROATIA

## Abstract

**Introduction:**

Common cold (CC) symptoms arise from an inflammatory response treatable with cineole and generally peak within two days, which complicates research implementation. We therefore explored the benefits of early cineole administration with enrolment of participants prior to CC onset.

**Methods:**

Out of 522 adults enrolled in our phase IV, open-label, non-randomized, exploratory clinical trial (EudraCT No. 2020-000860-51), 329 developed a CC and used 200 mg cineole (Soledum^®^, CNL-1976) t.i.d. for max. 15 (± 2) days. Primary endpoint was burden of disease based on the Wisconsin Upper Respiratory Symptom Survey (WURSS-11).

**Results:**

Comparing three strata based on time to treatment (≤ 12 h, > 12 to ≤ 24 h and > 24 h), earliest treatment resulted in lowest AUC-WURSS (Spearman correlation coefficient of 0.36) and reduced the overall burden of disease by 38% (p < 0.0001). Earlier and lower symptom severity peak resulted, with shorter time to remission (average 8.9 vs. 10.7 days with latest treatment initiation, p < 0.05), and higher and faster recovering quality of life (p < 0.05). Tolerability was mostly rated as “very good”, with adverse events of suspected causal relationship reported in 4.3% of participants.

**Conclusions:**

Early intervention shows clinical benefits relevant for the effective treatment of CC with cineole.

## Introduction

The common cold is an infection of the upper respiratory tract associated with respiratory symptoms such as sneezing, sore throat and runny nose, as well as more general symptoms including headache, malaise, and fever. It is a popular term coined and generally used for the disease category of upper respiratory tract infections, which are mostly of viral origin and, depending on the most affected anatomic area, classified as rhinitis, rhinosinusitis, and pharyngitis [1,2]. Common cold is frequently accompanied by acute bronchitis, making differential diagnosis challenging [3]. It is usually a self-limited infection, but can also spread to adjacent organs resulting in different clinical manifestations, and occasionally also predispose to bacterial complications [4]. On average, a common cold lasts for 7 to 10 days, but some patients can be symptomatic even after 3 weeks. Symptom types and severity vary among individuals and depending on the underlying pathogen, for example with fever being more common in children but rather rare and mild in adults [5]. High incidence combined with substantial symptomatic and functional impairment make the common cold a significant public health issue leading to the need for professional consultation and sick leave [1,4,6]. On average, adults suffer from 2 to 3, and children from up to 5 cold episodes per year [2]. Around 30% of school absences and 40% of work absences ultimately result from common cold and other acute respiratory tract infections [7].

Common cold being caused by various virus types with different pathogenetic mechanisms makes universal treatment difficult to develop, rather allowing for a symptomatic approach aimed at relieving burden of disease [4,8,9]. Aside from symptomatic treatment, common cold therapy is frequently associated with inappropriate use of antibiotics, exposing the patients to related side effects and contributing to the development of antibiotic resistance [8,10]. Appropriate treatment of common cold is therefore also vital for saving antibiotics. Cineole is an anti-inflammatory monoterpene present in different eucalyptus species and various other plants such as tea tree, rosemary, sage and cinnamon, and is also known for its mucolytic and mucoregulatory, antioxidant, bronchodilatory and antiviral properties [11–16]. Some of the underlying molecular mechanisms include the induction of interferon regulatory factor 3 (IRF3), the control of nuclear factor kappa-light-chain-enhancer of activated B cells (NF-κB) along with decreasing expression of mucin genes (MUC2, MUC19) [17]. Beneficial effects of cineole have previously been described for various respiratory conditions including bronchial asthma, chronic obstructive pulmonary disease (COPD), sinusitis, and acute bronchitis [7,17–21]. Cineole notably improves symptoms of a common cold without significant adverse effects and has also been recommended as an integrative remedy for the symptomatic improvement of patients with mild and uncomplicated infections caused by coronaviruses [22,23].

Conducting clinical trials on common cold treatment is complicated by its short-lived and self-limiting character [24]. Severity of common cold symptoms increases rapidly and usually peaks within the first 2 to 3 days [4,24]. Enrolling patients in a clinical trial after symptom onset therefore limits the therapeutic potential and complicates any comparison between treatment groups. We thus aimed to establish a novel clinical trial approach with enrolment of healthy individuals with previous history of common cold in order to be able to characterize symptoms and progression of an acute common cold episode from its very onset. In light of the previously described benefits of early intervention for the treatment of influenza [4,25,26], we hypothesized an analogous advantage of early treatment with cineole for reducing the burden of disease and duration of common cold. Our exploratory study was therefore focused on investigating the effects of different treatment timings for cineole. Corresponding advantages of early cineole treatment were ultimately reflected in the findings of our study.

## Methods

### Study design

We report here on a phase IV, prospective, open-label, non-randomized, exploratory, multicentre clinical trial whose primary objective was to investigate the impact of cineole treatment timing on the course of a common cold in adult patients with or without acute bronchitis. The clinical trial design was selected analogously to a previous study on acute viral respiratory disease investigating potential benefits of early treatment initiation [26]. Present study was conducted between September 2020 and May 2021 in 25 suitable trial centres in Germany specializing in otolaryngology, internal medicine and general practice, with 23 sites actively involved in recruiting subjects. Patients presenting for consultation in one of the respective outpatient settings were offered the possibility of participating in the trial, irrespective of reasons for their consultation. Recruitment could also take place via letters, emails, posters, flyers, announcements or advertisements.

In a novel approach to common cold research, healthy subjects were enrolled in order to be able to investigate the progression of a common cold from its very onset, through the treatment phase and into remission. Furthermore, the use of an electronic diary (eDiary) ensured adequate and timely collection of relevant patient-reported outcomes. Principal enrolment criteria were age between 18 and 70 years and recollection of at least one common cold episode in the preceding winter season. Principal exclusion criteria were any chronic diseases with potential impact on a common cold or on the tolerability of the investigational product. These were for example ear, nose, throat, and respiratory tract diseases such as chronic bronchitis as defined by the World Health Organization, WHO [27], COPD, bronchial asthma, chronic active allergic rhinitis (e.g., dust mite allergy), chronic rhinosinusitis, cystic fibrosis, immunosuppression and clinically relevant renal or liver disease. Other key exclusion criteria were known hypersensitivity to cineole or any other compound of the investigational product, as well as a previous SARS-CoV-2 infection. Smokers were enrolled only if free of any related respiratory symptoms or disease. Following therapies were not allowed within 7 days prior to second visit or as concomitant treatments during the study: systemic antibiotics, glucocorticosteroids, β2-mimetics, theophylline supplements; common cold or cough medication e.g., expectorants or mucolytics, drops/liquids for nasal decongestion, antitussives, essential oils, phytotherapeutics; systemic non-steroidal anti-inflammatory drugs / analgesics (except low dose acetylsalicylic acid); drugs without marketing authorization (in general or for specific indications); ACE blockers, apart from long-term therapy over at least 8 weeks on a stable dose prior to patient enrolment and without cough as side effect. A total of 4 visits took place across an individual trial duration of up to 8 months, with the screening phase (period between first visit and common cold onset) lasting up to 7.5 months. Second study visit followed within 48 h after symptom onset (i.e., start of the eDiary documentation) and included confirmation of diagnosis by the investigator and cineole (CNL-1976, Soledum^®^ Kapseln forte) treatment initiation (one 200 mg capsule, 3x per day). Common cold definition closely followed the criteria previously described by Barrett et al. [28]. Diagnosis of a common cold (with or without acute bronchitis i.e., an upper respiratory tract infection) was confirmed by the investigator based on clinical symptoms, patient’s perception of having a common cold with an onset ≤ 48 h prior to treatment initiation, and a total Jackson symptom score of ≥ 3 points for selected criteria including nasal discharge, nasal obstruction, sneezing and sore throat. An interim visit took place 3 days into the treatment phase (visit 3). Intake of cineole continued until recovery from the common cold or otherwise up to 16 days of treatment, with the final visit taking place at recovery or day 14 of treatment. Recovery was defined as 2 consecutive ratings of “0” in the eDiary for the Wisconsin Upper Respiratory Symptom Survey (WURSS-11) items 1–10. Paracetamol (500 mg tablets) served as rescue medication, to be used “as needed” in case of fever and/or profound or protracted headache.

### Ethics approval and consent to participate

Present clinical trial was conducted in accordance with the Declaration of Helsinki on Ethical Principles for Medical Research Involving Human Subjects adopted by the General Assembly of the World Medical Association (WMA 2013). Planning and implementation of the clinical trial followed the appropriate national laws, the principles and guidelines for good clinical practice (GCP) laid down in Directives 2001/20/EC (ICH Topic E9) and 2005/28/EC (ICH Topic E2A) of the European Parliament and the International Conference on Harmonisation (ICH) Guideline for Good Clinical Practice E6 (R2) as well as SOPs of Cassella-med and Pharmalog based on the ICH-GCP guidelines. All participants gave written informed consent prior to taking part in the trial.

According to local regulations, the protocol was reviewed and approved by the national competent authority, the German Federal Institute for Drugs and Medical Devices (*Bundesinstitut für Arzneimittel und Medizinprodukte*, *BfArM*; approval no. 61-3910-4044155). Approval was also obtained from the Ethics Commission of the State of Berlin at the State Office for Health and Social Affairs (*Ethikkommission des Landes Berlin des Landesamts für Gesundheit und Soziales*), the local competent commission for the coordinating trial site (approval no. 20/0147 –IV E 15). The trial was prospectively registered with EudraCT under the number 2020-000860-51 (date of registration: 04 May 2020).

### Data collection via an eDiary

Electronic data collection was performed via a validated web-based eDiary (Climedo Health App^®^) on the participants’ personal devices (e.g., smartphone, tablet or computer). In order to ensure proper use, participants were trained by the investigator and/or trial site staff on the eDiary app during their initial study visit. The eDiary also facilitated appropriate gathering of data through reminders appearing on participants’ devices in accordance with the respective trial phase:

- Starting at one week following their initial visit and until recording of patient-reported outcomes began, participants received a weekly reminder to immediately document any onset of common cold symptoms and fill out the WURSS-11 form [29]. Documentation could start anytime, except in case of an acute respiratory infection diagnosed at the initial visit, for which a three-week gap was set before the first reminder, ensuring the infection would clear before documentation began. Participants were also instructed not to start the eDiary documentation in case such infection had not cleared within 3 weeks after diagnosis.
- From the onset of common cold and start of eDiary documentation, and until participants recovered or reached their final study visit, a reminder was provided twice a day for the WURSS-11 form, and every evening for documenting the intake of cineole or rescue medication. If the documentation was missed, the reminders were repeated up to 2 additional times.

Symptom onset based on the eDiary was defined as the timepoint of first registering a common cold and reporting symptoms via the WURSS-11 questionnaire. At the start of the eDiary documentation, subjects were asked to fill in date and time of first occurrence of common cold symptoms. If participants failed to fill out the WURSS-11 form within 12 hours after experiencing first symptoms, the current common cold episode was not considered adequately reported for inclusion in the trial and the eDiary remained locked for a subsequent 3 weeks, after which it could be used to document a new common cold episode. Time stamps were automatically recorded and used to monitor compliance. Beyond reporting symptoms via WURSS-11, participants also regularly recorded the use of cineole and rescue medication. Completeness of the participants’ recordings and medication adherence were ascertained by the investigators at trial visits. Participants whose common cold diagnosis could not be verified by the investigators were instructed to refrain from further documentation for the current episode but were allowed to remain in the screening phase and could subsequently become eligible for new common cold documentation.

### Outcomes

Primary endpoint was burden of disease based on an area under the curve (AUC) analysis of the WURSS-11 total score. WURSS-11 had been derived from the WURSS-21 score with the intention of reducing questionnaire completion time and increasing the response rate, and it preserves the reliability and domain structure of its predecessor [29]. WURSS-21 was originally developed as means of standardizing the evaluation of the common cold impact and includes symptom and functional domains relevant for patients. Due to a lack of widely accepted criteria or tests for diagnosis and since most common cold treatments are initiated upon self-diagnosis, WURSS is based on self-assessment and as such has been used in previous clinical trials [1,28]. WURSS-11 enables patient-reported assessment of the overall burden of disease and its progression (feeling sick, common cold condition compared to the previous day), severity of specific nasal symptoms (runny nose, plugged nose, sneezing), throat symptoms (sore throat, scratchy throat, cough), feeling tired as a general symptom as well as aspects related to quality of life (clear thinking, accomplishing daily activities) [30]. Items are rated on a 7-point scale with 0 corresponding to “no” manifestation and 7 corresponding to “severe” manifestation, except for common cold condition, which is rated from “very much better” to “very much worse”. Secondary endpoints based on WURSS-11 included symptom severity, quality of life (QoL) and time to symptom relief and remission. Symptom severity peak was defined as the highest observed mean daily WURSS-11 total score. Symptom relief was defined as a reduction in the mean daily total score by at least 50% of the assessed symptom severity peak, and remission was considered as reached when daily single score of item 1 was ≤ 1 accompanied by only one symptom scored ≤ 3 and all other symptoms scored 0.

Additional secondary endpoints included investigator assessment of symptoms via the Jackson Symptom Score (JSS) and the presence of bronchitis via the Bronchitis Severity Score (BSS). JSS is comprised of 8 common cold symptoms: nasal discharge, nasal obstruction, sneezing, sore throat, headache, malaise, chilliness, and cough [31]. Each symptom is scored on a 4-point scale (0 = none / not present; 1 = mild; 2 = moderate; 3 = severe), resulting in a total score ranging between 0 (“no symptoms”) and 24 (“severe symptoms”). Total JSS was calculated as the sum of the individual item assessments. BSS assessment is based on the investigator’s clinical evaluation in conjunction with patient feedback and therefore combines objective and subjective items [3]. Five most important features of acute bronchitis are represented in the BSS i.e., cough, sputum production (expectoration), rales/rhonchi (auscultation), chest pain during coughing, and dyspnoea. Items are assessed by the investigator using a 5-point verbal rating scale ranging from 0 to 4 (0 = absent; 1 = mild; 2 = moderate; 3 = severe; 4 = very severe). The total BSS score ranges between 0 (“no acute bronchitis”) and 18–20 (“very severe acute bronchitis”). The BSS has previously been used to evaluate the efficacy of various herbal therapies for acute bronchitis [19,32,33]. For the purposes of the present trial, a BSS assessment was only undertaken if “cough” was reported in the JSS. Acute bronchitis was considered as present if the total BSS score was > 2 for at least one trial visit.

Global judgement of efficacy and safety by the investigator and the global judgement of safety by the patient were provided based on a 5-point rating scale (0 = very good, 1 = good, 2 = moderate, 3 = poor, 4 = very poor). Days of sick leave and bed rest, and incidence of adverse events (AEs) were also investigated as secondary endpoints.

### Statistical analysis

#### Sample size calculation

In light of the exploratory nature of the trial aiming to investigate the complete course of a common cold from the very first symptom onset under real world conditions, information available for any sample size calculation was sparse. The trial was consequently designed based on confidence interval precision for the primary endpoint, the WURSS-11 score, rather than on power considerations based on statistical testing. Based on literature findings regarding previous common cold trials, the symptom severity peak was assumed to be at a total WURSS-11 symptom score of 15, allowing an estimate for the 95% confidence intervals having a width of mean ± 2 points, with an expected balanced distribution of subjects into 3 time-to-treatment strata with 110 subjects per stratum. The estimated total WURSS-11 symptom scores would consequently have a precision of 4 points at symptom severity peak, considered a substantial precision for the clinical interpretation of key results. Assuming only 66% to 85.5% of screened participants would experience a common cold episode fulfilling all eligibility criteria within a screening phase of up to 7.5 months, a target of approximately 400 to 500 subjects was set for the screening in order to achieve 330 patients eligible for analysis.

#### Analysis of study endpoints

The primary efficacy endpoint, AUC-WURSS, was derived from the mean daily total WURSS-11 scores as averages of an evening and subsequent morning assessment for each item. If only one of the two assessments was available, then that assessment was entered as the mean item score for the given day. Mean daily total score was calculated as the sum of the mean item scores for questions 2 to 10. The more general items 1 (“how ill the patient feels”) and 11 (“subject’s impression of common cold progression compared to previous day”) were not used for this derivation. Sum of mean daily total WURSS-11 scores for day 1 to 17 was obtained using trapezoidal approximation, which corresponds to a two-week treatment (assuming that treatment starts 2 days after symptom onset). Analysis of the secondary endpoints mean daily total score, mean daily group score (symptom domain and quality of life) and daily single item scores was conducted analogously to that of the mean daily total score described for the primary endpoint. Imputation of values for the mean daily group scores of WURSS-11 was analogous to that of the mean daily total score.

#### Data imputation

Imputation of values was undertaken for specific assessments according to the type of missing data. Incomplete dates containing only month and/or year and not assignable to a specific study date or visit were assigned days and months representing the longest/shortest possible period, as appropriate. Regarding common cold recovery, population-based imputation methods were considered too unspecific in light of the subjective character and the heterogeneous aetiology of the disease as well as its self-limiting nature characterized by a constant symptom decline after their peak and into recovery. Missing WURSS-11 assessments until recovery (e.g., due to the presence of symptoms or early withdrawal) were replaced through individual quadratic regression models fitted to available mean daily total symptom scores of the participants. Common cold recovery day was considered as reached when the estimated regression line crossed zero, whereby different model functions could be considered if zero had not been reached. Missing values of WURSS-11 single items after recovery were imputed up to day 17 based on the Last Observation Carried Forward (LOCF) principle (i.e., all values were set to zero after recovery, in accordance with resolution of common cold symptoms). In case of missing mean daily total score before common cold recovery, values were imputed up to the estimated duration of common cold based on the individual regression line used to define day of common cold recovery and used to derive the AUC-WURSS. For the mean daily group scores of WURSS-11, individual quadratic regression was fitted based on available mean daily group scores and data imputation performed up to symptom day 17 based on the individual regression line.

#### Statistical tests

Statistical analysis was performed using the Statistical Analysis System (SAS) software, Version 9.4. Analysis was based on a modified intention-to-treat (mITT) set comprised of patients who received at least 4 treatment doses within the first 4 days after visit 2 with a maximum of 1 day without treatment, provided at least 5 valid WURSS-11 assessments within the first 7 days after symptom onset with a maximum of 2 days without assessment and had not taken antibiotics after treatment initiation due to an upper respiratory tract infection. Scope of the trial was purely exploratory, and all statistical tests were performed on a nominal significance level of 5% without controlling for multiplicity issues. Definition of strata was data-driven and refined during an interim analysis planned to take place after approximately 60 subjects of the mITT set had completed the trial. This approach enabled ascertaining that an adequate number of patients per stratum would be achieved, particularly in light of practical difficulties potentially complicating an early consultation with a physician in a real-world setting. The interim analysis was also conducted to gain a preliminary insight into drop-out rates, efficacy, and heterogeneity of collected data and included the following assessments: analysis of disposition / drop-out patterns including incidence of SARS-CoV2, demographics, baseline characteristics / confounders, compliance, exposure to study medication, AUC-WURSS scatter plot including correlation analysis, descriptive summary of mean daily total WURSS-11 score by symptom day, symptom severity peak and duration of common cold. Its results were ultimately based on data of 95 mITT subjects and summarized in an interim report detailing any potential changes in conduct or analysis of the trial together with an evaluation of their potential impact.

A basic general linear model (termed GLM1) was used to investigate the influence of time to treatment on the AUC-WURSS, with age, gender, previous influenza vaccination and baseline WURSS-11 score as additional fixed factors. A pairwise test on stratum differences was performed using the stratum with the longer time-to-treatment interval as reference. Time courses for the secondary endpoints were analysed via mixed models for repeated measurements (MMRM), time to remission via an Accelerated Failure Time (AFT) model, and occurrence of acute bronchitis via a logistic model. Comparability of the investigated strata was ascertained via two types of sensitivity analyses, a confounder-based analysis and a model based on propensity score methods involving relevant confounders. Confounder-based analysis involved a linear regression model performed via a “backward elimination procedure” with respect to the primary endpoint including the following potential confounders: treatment stratum, gender, previous influenza vaccine (yes/no), smoking (non/ex-smoker, smoker), work status [(self-)employed or not], number of common cold episodes during previous winter (ad-hoc categorization; 1,2,3 and more episodes), alcohol consumption (none / low level / substantial), baseline total WURSS score (ad-hoc categorization, ≤ 27 and > 27). Risk factors without significance on a 5% level were eliminated. Remaining risk factors (time to treatment stratum, previous influenza vaccination, alcohol consumption, work status and baseline WURSS) were considered relevant confounders in terms of common cold healing and were included in the sensitivity analysis for the primary endpoint (GLM2) and selected secondary endpoints (sensitivity analyses based on MMRM, AFT and logistic models). Results of confounder-based sensitivity analyses were consistent with those of respective basic model calculations and are reported in the manuscript, unless otherwise specified. The propensity score analysis was based on scores calculated via a logistic regression model with strata as a dependent variable and including the four confounders additionally identified as relevant for the primary endpoint. This enabled assessing the probability of subjects with a specific risk factor combination entering the respective strata. Inverse probability weights were then used to adjust for differences in risk between the strata. Sensitivity analyses without imputed data were also performed for the basic model calculations. Categorical data are reported with two-sided 95% confidence intervals (CI), where applicable. Means are presented with standard deviation. Unless otherwise specified, percentages are based on the reported stratum.

## Results

**Fig 1:**
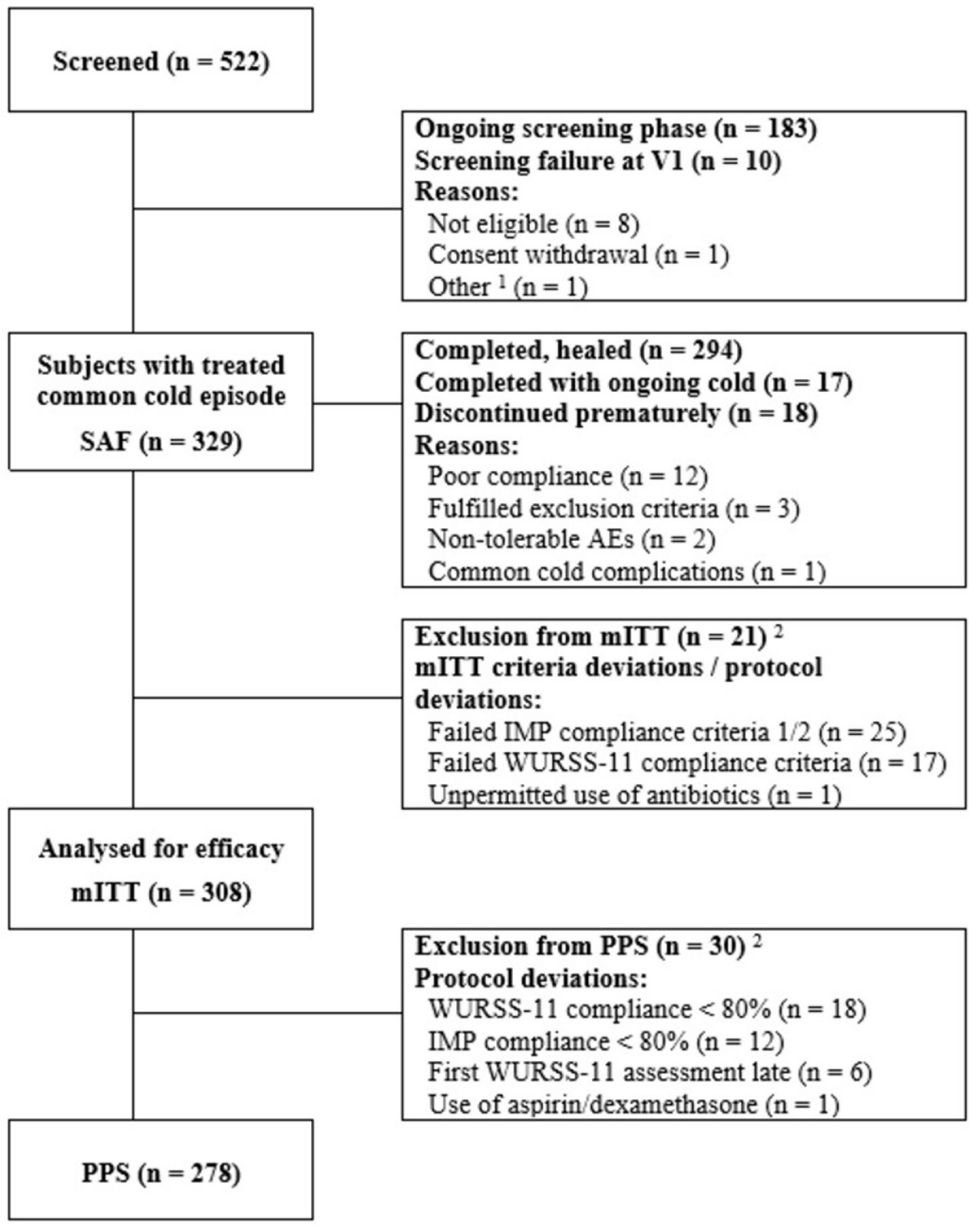
Patient flow. ^1^Patient failed screening due to chronic sinusitis. ^2^A single patient could have more than one modified ITT (mITT) criteria deviation/protocol deviation.

522 participants were enrolled in the trial, with 329 (63%) ultimately developing a common cold treated with at least one dose of cineole (Fig 1). One patient could not be assigned to any stratum due to incomplete data. A total of 18 patients prematurely discontinued the treatment and their participation in the trial, 12 due to poor compliance, 2 due to AEs (vomiting and diverticulitis) and 1 due to a common cold complication (Tonsillitis bacterial).

The mITT population was comprised of 308 patients, out of which 122 were in the stratum ≤ 12 h, 88 in the stratum > 12 to 24 h, and 98 in the stratum > 24 h. Demographic characteristics of the three strata were fairly balanced, with some differences regarding the male-to-female ratio and alcohol consumption (Table 1). The basic GLM1 analysis did not show any impact of gender on efficacy outcomes. The imbalance in alcohol consumption was addressed and adjusted in the sensitivity analysis GLM2.

**Table 1:** Subject baseline characteristics (mITT).

	Time to treatment stratum						Total	
Parameter Category	≤12 h (N = 122)		>12 to 24 h (N = 88)		>24 h (N = 98)		(N = 308)	
	n	%	n	%	n	%	n	%
**Gender**								
Female	86	70.5	52	59.1	53	54.1	191	62.0
Male	36	29.5	36	40.9	45	45.9	117	38.0
**Smoking status**								
Non-smoker	87	71.3	64	72.7	73	74.5	224	72.7
Ex-smoker	13	10.7	0	0	5	5.1	18	5.8
Smoker	22	18.0	24	27.3	20	20.4	66	21.4
**Working status**								
(Self-) employed	94	77.0	66	75.0	79	80.6	239	77.6
Not employed	12	9.8	11	12.5	4	4.1	27	8.8
Student	16	13.1	11	12.5	15	15.3	42	13.6
**Influenza vaccination for the coming/current winter season**								
No	98	80.3	72	81.8	87	88.8	257	83.4
Yes	24	19.7	16	18.2	11	11.2	51	16.6
**Confounder categorization for alcohol consumption**								
None	90	73.8	72	81.8	66	67.3	228	74.0
Low level consumption (males < 20 g, females < 10 g)	15	12.3	6	6.8	7	7.1	28	9.1
Substantial consumption (males ≥ 20 g, females ≥ 10 g)	17	13.9	10	11.4	25	25.5	52	16.9

**Age (years)**				
Mean	37.9	38.4	39.8	38.7
SD	12.7	13.0	14.1	13.2
**BMI (kg/m** ^**2**^ **)**				
Mean	25.80	26.00	27.02	26.25
SD	4.67	4.74	5.02	4.82
**Common cold episodes during previous winter, Sep to Mar (number)**				
Mean	2.3	2.3	2.3	2.3
SD	1.1	1.6	0.9	1.2
**Alcohol consumption (g/day on average)**				
Mean	3.7	2.9	6.0	4.2
SD	7.5	7.5	9.3	8.2
**Duration of smoking in smokers only (years)**				
Mean	17.2	21.5	23.3	20.6
SD	9.0	12.5	13.3	11.8
**Baseline WURSS score**				
Mean	27.31	29.30	26.80	27.71
SD	12.17	11.86	11.65	11.92

WURSS: Wisconsin Upper Respiratory Symptom Survey.

Mean daily dose of 568.87 (± 60.15) mg/day was well in range with the prespecified cineole dose of 600 mg/day. Mean duration of treatment was 9.6 (± 3.4) days and similar among the three strata. 14.3% of subjects required rescue medication during the trial, with no apparent differences between the strata.

### Primary outcome

**Fig 2:**
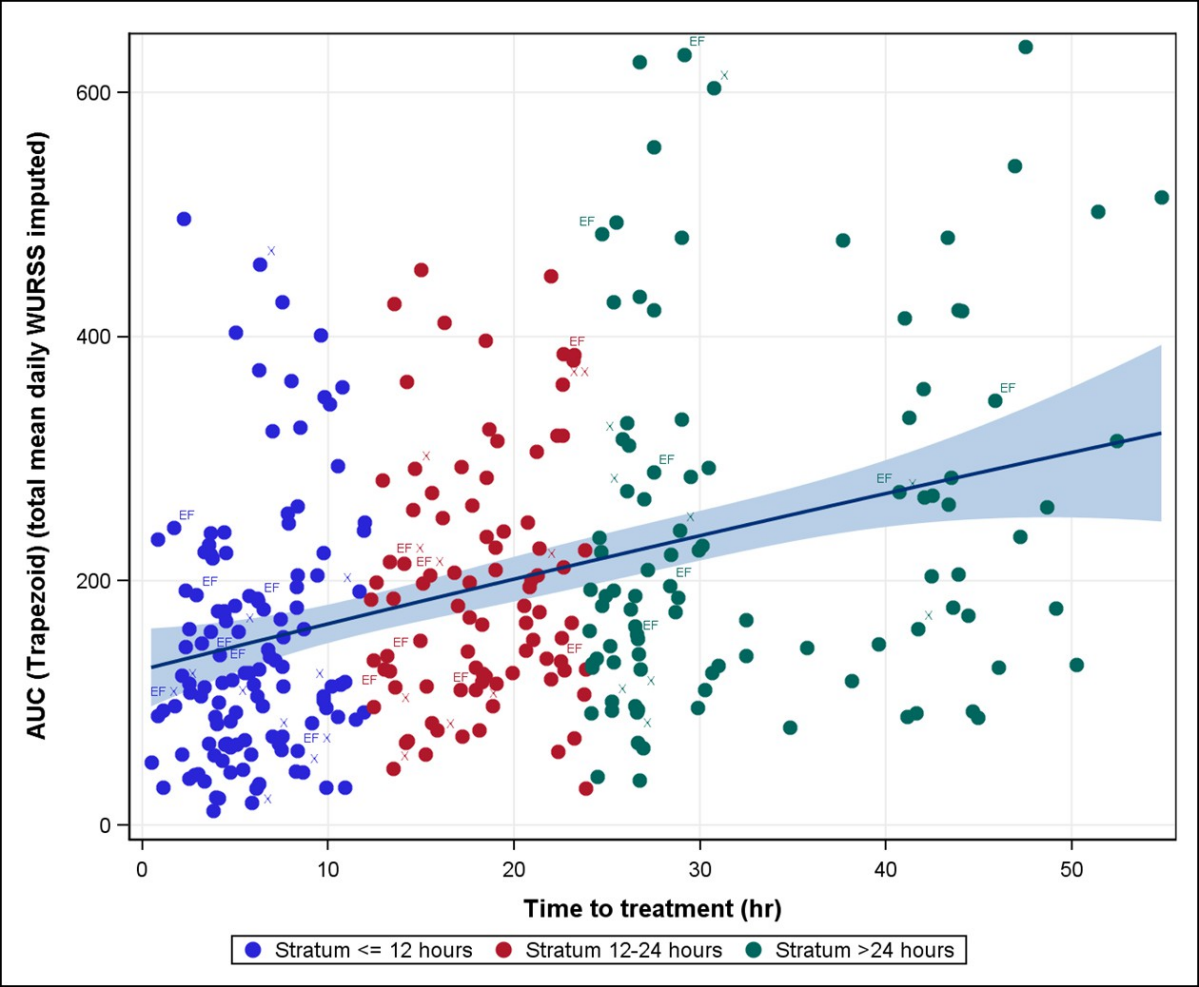
WURSS-11 AUC vs. time to treatment.

Early treatment resulted in lower AUC WURSS-11 values, with a moderate correlation corresponding to a Spearman correlation coefficient of 0.36 (Fig 2). Median WURSS-11 AUC progressively increased with longer time to treatment, from 120.8 (CI 11.5 to 496.7) in stratum ≤ 12 h to 179.8 (30 to 454.6) in stratum > 12 to 24 h and 194.6 (37 to 637.5) in stratum > 24 h. GLM1 revealed a significant effect of time to treatment on the WURSS-11 AUC (p < 0.0001), which was confirmed by the sensitivity analysis GLM2.

**Fig 3:**
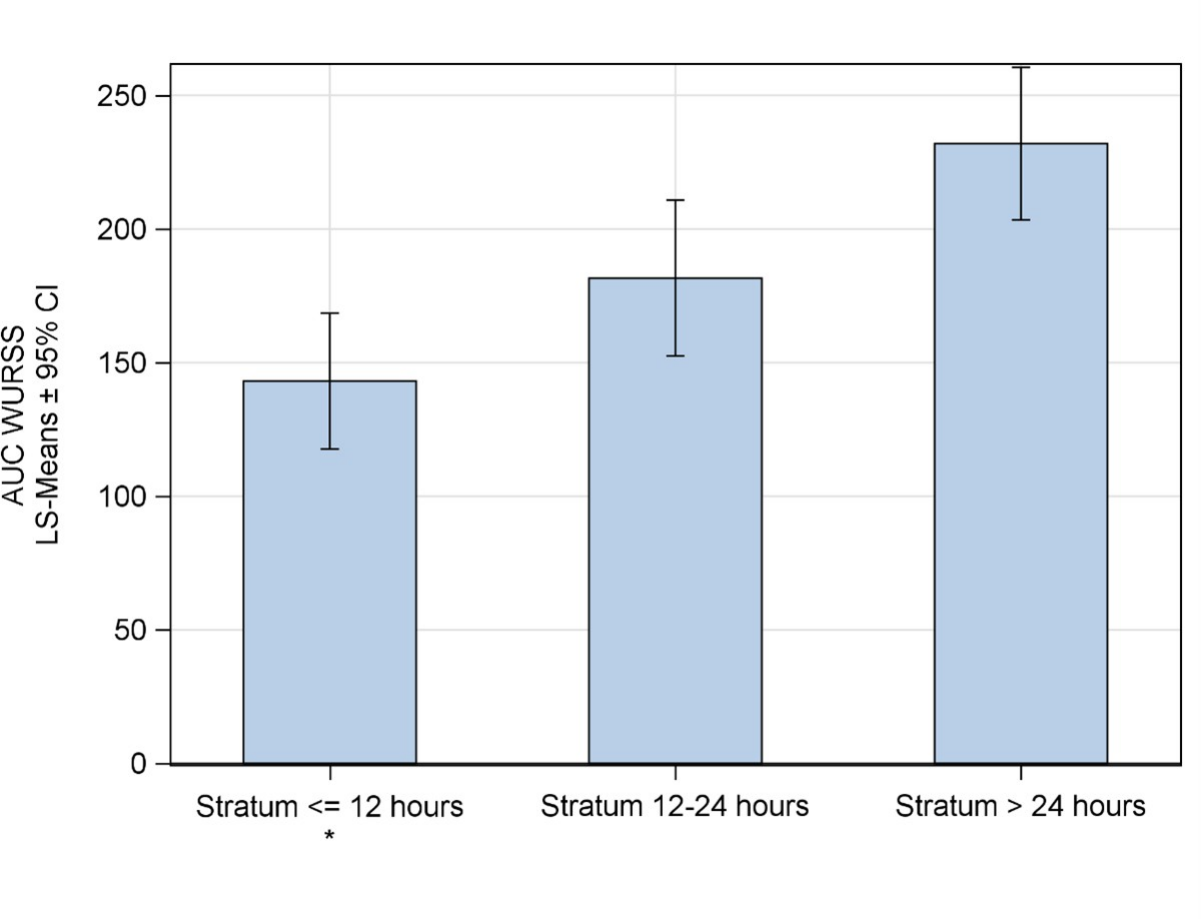
GLM analysis for the WURSS-11 AUC. Bars represent the GLM2 model LS means of the mean daily total WURSS-11 AUC for individual time to treatment strata, and the whiskers represent the 95% CIs of the respective LS means. * p < 0.05.

Pairwise comparisons based on non-overlapping CIs of least squares means (LS means) in GLM2 yielded significant differences between stratum > 24 h (LS mean 232 [203.4 to 260.5]) and ≤ 12 h (LS mean 143.1 [117.7 to 168.5]; Fig 3), corresponding to a 38% reduction in burden of disease for the shortest compared to longest time to treatment. Results were confirmed by the propensity score analysis and the sensitivity analysis without imputed values (S1 and S2 Tables).

### Secondary outcomes

#### Efficacy outcomes

**Fig 4:**
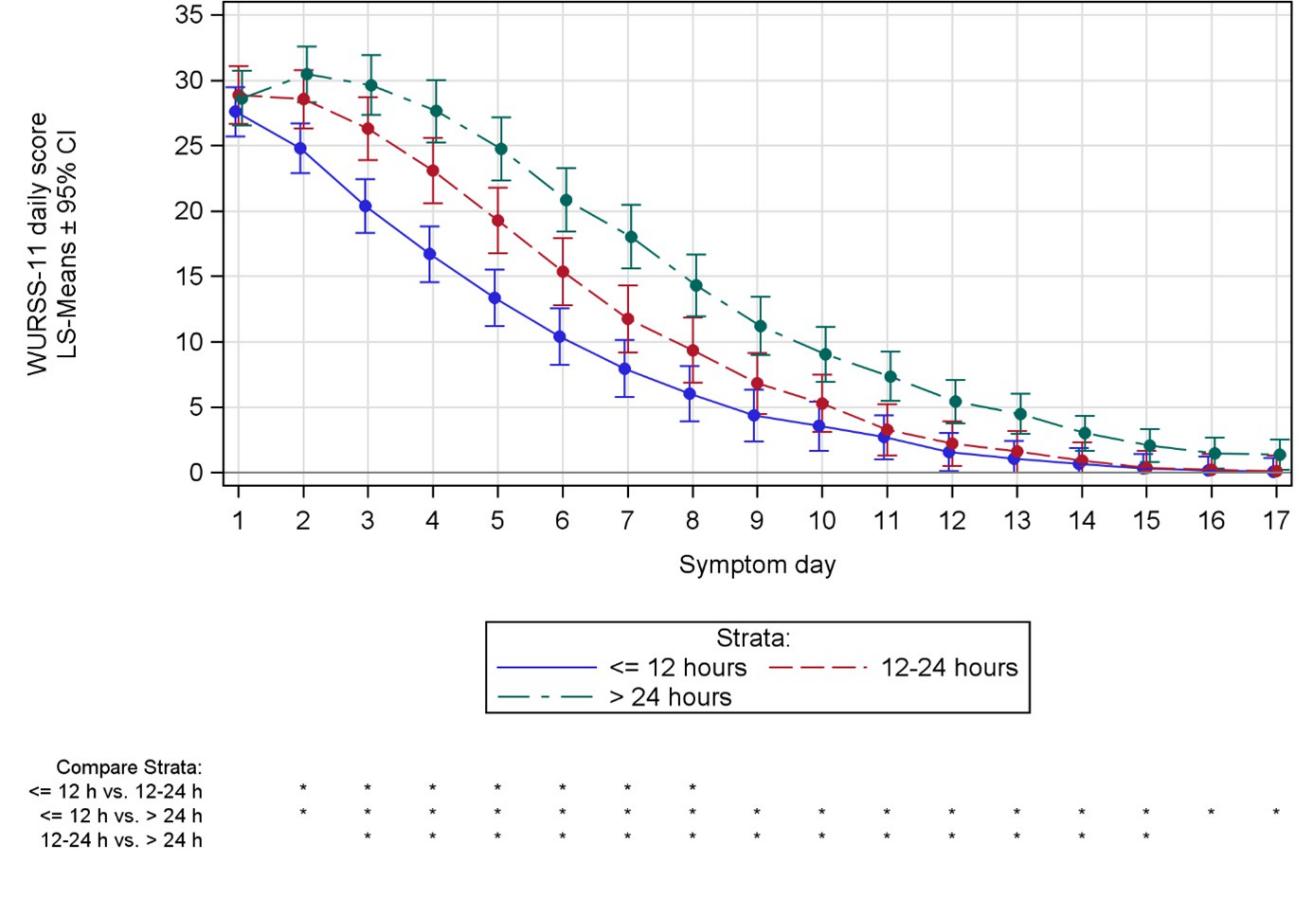
WURSS-11 mean daily total score. Curves represent the MMRM model LS means of the WURSS-11 mean daily total score during the common cold episode for individual time to treatment strata (sensitivity analysis), and the whiskers represent the 95% CIs of the respective LS means. * p < 0.05.

*Confounder-based analysis*.Time curve of the WURSS-11 mean daily total score showed a substantially faster decline in case of the earliest time to treatment stratum, starting from day 1 of the common cold episode in contrast to day 3 and 4 in the remaining two strata (Fig 4). An average WURSS-11 mean daily total score below 1 was seen as early as day 14 for strata ≤ 12 h and > 12 to 24 h but remained above 1 until day 17 for stratum > 24 h. Furthermore, analysis of LS means confirmed WURSS-11 mean daily total score was significantly lower with stratum ≤ 12 h compared to > 24 h from day 2 to 17, indicating a reduced burden of disease over the course of the common cold episode (p < 0.05).

**Fig 5:**
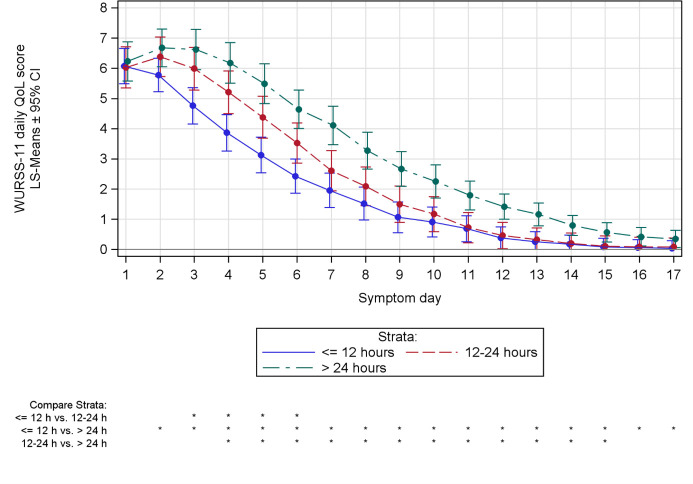
Quality of life during the common cold episode, based on WURSS-11. MMRM analysis depicted analogously to Fig 4. * p < 0.05.

Similar pattern was also observed with the mean daily group scores of the WURSS-11 (symptom and QoL domain). Scores for the symptom domain showed analogous differences in decline described for the mean daily total score, with LS means analysis indicating a significant difference between the earliest and latest time to treatment for most of the common cold episode (day 2 to 15, S3 Table). Scores of the QoL domain followed the same pattern and showed equivalent significance of time to treatment between day 2 and 17, indicating higher quality of life with early treatment initiation (p < 0.05; Fig 5). In addition, average mean daily QoL score below 1 was observed by day 10 for stratum ≤ 12 h, day 11 for stratum > 12 to 24 h, and day 14 for stratum > 24 h, suggesting that quality of life also recovered sooner with early treatment. As was the case with the primary endpoint, sensitivity analyses for the above-described secondary endpoints without imputed values showed similar results to the model calculations containing such values (S4–S6 Tables).

**Fig 6:**
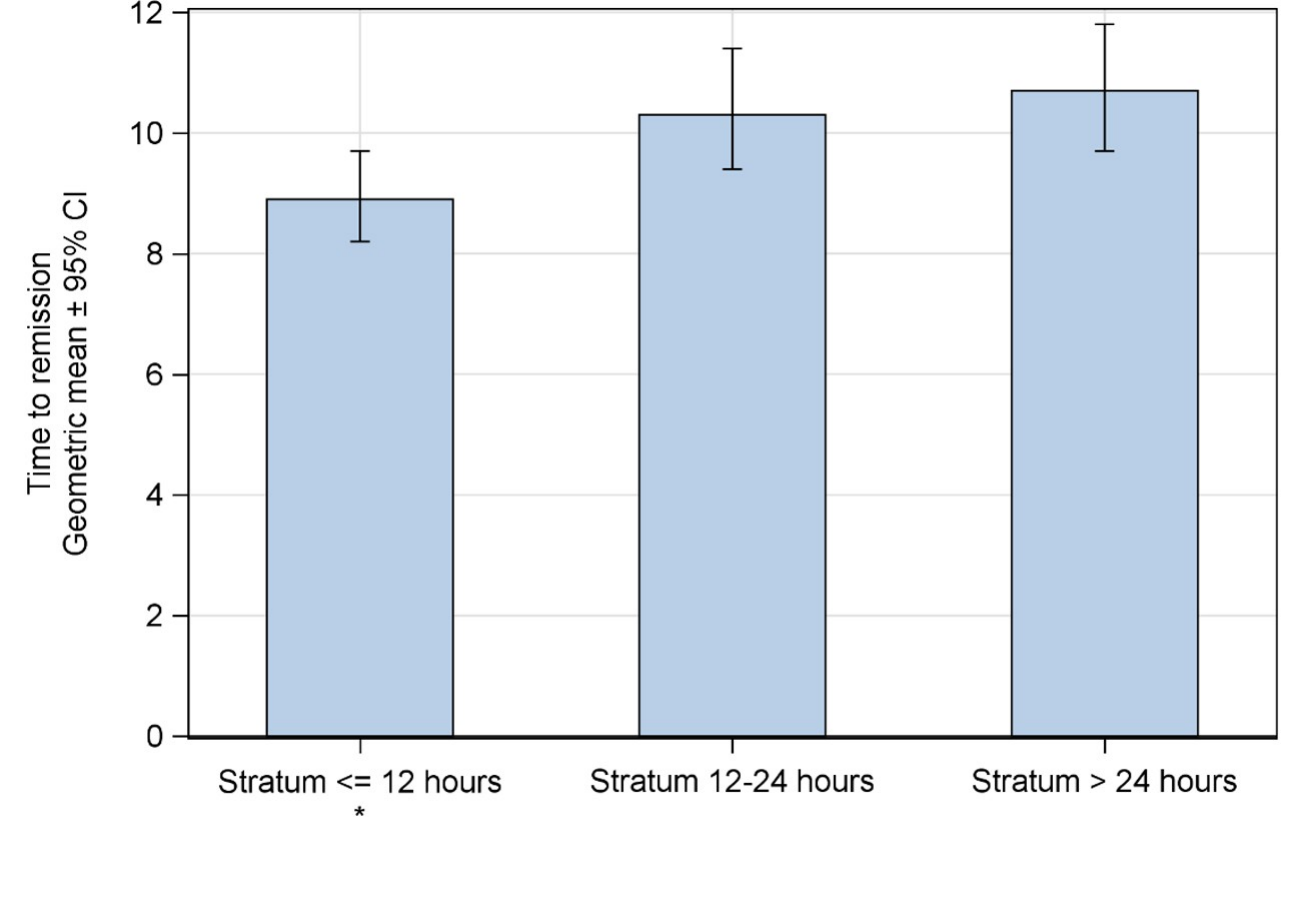
Time to remission; bars represent the AFT model geometric means of the endpoint for individual time to treatment strata (sensitivity analysis), with 95% CIs as whiskers. * p < 0.05.

Time to remission was shortest with the earliest treatment, with a difference of almost 2 days (corresponding to 16.7%) compared to the longest time to treatment: 8.9 (8.2 to 9.7) days for stratum ≤ 12 h, 10.3 (9.4 to 11.4) days for stratum > 12 to 24 h, and 10.7 (9.7 to 11.8) days for stratum > 24 h (Fig 6). This resulted in a significant acceleration factor of 0.8 (0.8 to 0.9) for earliest compared to longest time to treatment, whereas no significant difference was observed between the > 12 to 24 h and > 24 h stratum (acceleration factor 0.96 [0.9 to 1.1]).

39% of participants developed acute bronchitis during the trial, with predominantly mild manifestation (S7 Table). Proportion of participants with acute bronchitis increased with later treatment initiation. Prevalence of mild acute bronchitis was lower with earliest treatment initiation, while the prevalence of moderate and severe manifestation was more similar across the three strata. Observed differences in the occurrence of acute bronchitis were not statistically significant as determined by the confounder-based analysis.

*Descriptive statistics*. Above-described advantages of early treatment for faster symptom improvement were consistently reflected in individual symptom scores of the WURSS-11 (S8 Table). Earlier treatment also resulted in a lower median symptom severity peak, with a reduction of approximately 4 points compared to later treatment initiation (S9 Table). Furthermore, symptom severity peak was reached one day sooner with earliest treatment, median on day 1 (1 to 9) in the stratum ≤ 12 h vs. day 2 (1 to 8) in the > 12 to 24 h stratum and day 2 (1 to 11) in the > 24 h stratum. Earlier symptom peak was accompanied by shorter time to relief, with on average 1 day gained through early treatment (S10 Table).

Benefits of early treatment for lower burden and faster improvement of symptoms determined based on the patient-reported WURSS-11 were confirmed by the investigators’ JSS assessment (S11 Table). However, results for the strata ≤ 12 h and > 12 to 24 h were rather similar based on the JSS assessment. Mean number of bed rest days was 0.6 (± 1.4), with no apparent difference between the time to treatment strata (S12 Table). Mean number of sick leave days was 1.3 (± 2.5), whereby earlier treatment was associated with longer sick leave: 1.5 (± 2.9) days in the ≤ 12 h stratum and 1.4 (± 2.6) in the > 12 to 24 h stratum compared to 0.9 (± 2.0) in the > 24 h stratum. Investigators mostly rated the treatment efficacy as “very good” (for 76% of participants), with a minority reporting “moderate” (2.6%), “poor” or “very poor” (0.6%) efficacy. Prevalence of “very good” rating was somewhat lower for the ≤ 12 h stratum (70.5%) compared to the > 12 to 24 h stratum (75%) and > 24 h stratum (83.7%).

#### Safety outcomes

Tolerability of investigational product was similarly rated by investigators and participants, predominantly “very good” (investigator assessment in 80.9% of cases, and patient assessment in 68.1% of cases), followed by “good” (investigators: 15.8%; patients: 27.4%) and “moderate” (investigators and patients: 1.8% each). “Poor” tolerability was reported by none of the investigators, and 1.2% of patients, whereas “very poor” tolerability was reported in 0.3% of cases by both parties. 25 predominantly mild AEs were reported in 22 subjects (6.7%), out of which 17 events had suspected causal relationship with the cineole treatment. Most AEs did not require a change in treatment and had resolved by the end of the trial (S13 Table). One severe AE (Tonsillitis bacterial) was reported, without suspected causal relationship with the cineole treatment (Tables 2 and S13).

**Table 2:** Adverse events listing based on MedDRA classification.

	Total	
MedDRA System Organ Class Preferred Term	(N = 329)	
	AEs	n patients (%)
**AEs with a suspected causal relationship to cineole treatment**		
**At least 1 adverse event**	**17**	**14 (4.3%)**
**Gastrointestinal disorders**		
All Preferred Terms	17	14 (4.3%)
Abdominal pain	1	1 (0.3%)
Abdominal pain upper	3	2 (0.6%)
Diarrhoea	2	1 (0.3%)
Eructation	3	3 (0.9%)
Flatulence	1	1 (0.3%)
Gastrointestinal pain	1	1 (0.3%)
Nausea	5	5 (1.5%)
Vomiting	1	1 (0.3%)
**AEs without suspected causality**		
**All AEs**	**8**	**8 (2.4%)**
**Gastrointestinal disorders**		
All Preferred Terms	1	1 (0.3%)
Regurgitation	1	1 (0.3%)
**Infections and infestations**		
All Preferred Terms	5	5 (1.5%)
Diverticulitis	1	1 (0.3%)
Nasal herpes	1	1 (0.3%)
Sinusitis	1	1 (0.3%)
Tonsillitis bacterial	1	1 (0.3%)
Urinary tract infection	1	1 (0.3%)
**Nervous system disorders**		
All Preferred Terms	2	2 (0.6%)
Headache	1	1 (0.3%)
Sciatica	1	1 (0.3%)

AE: adverse event; MedDRA: Medical dictionary for regulatory activities.

## Discussion

The innovative design of our present study with enrolment of healthy participants and modern use of an eDiary enabled treatment and monitoring of an acute common cold episode from its very onset. Our principal finding is that early intake of cineole reduces the burden of disease based on patient-reported symptoms compared to later treatment initiation. The observed correlation between time to treatment and burden of disease is substantial considering that many different factors impact the manifestation of a common cold [5]. As our study was primarily focused on establishing the novel design and investigating the impact of time to treatment on relevant characteristics of a common cold, it does not feature a control group with a different treatment option or placebo. Future research into the effects of cineole with an appropriate comparator will therefore be relevant for further conclusions on efficacy and safety.

Common cold studies are notoriously difficult to implement due to the short-lived and self-limiting nature of the disease. As symptom peak occurs early on during a common cold episode, enrolling patients within the first 24 h after symptom onset is recommendable [24]. Appropriate enrolment of common cold patients has previously relied on patient recollection of symptoms and their onset [34–37]. However, patients reporting for treatment later in the course of their common cold than indicated by their symptom history has been identified as one of the major challenges in conducting common cold studies, together with subsequent poor medication compliance [24]. Research involving web-based recruitment and data collection has the advantage of early recruitment before a common cold onset but lacks clinical diagnosis confirmation and monitoring [38]. Our study addresses these issues through the recruitment of healthy patients and the use of an eDiary in order to accurately capture common cold onset and progression while minimizing recall bias. Furthermore, specialist consultation ensured accurate diagnosis and adequate capturing of disease progression into its resolution.

Our present study is, to our knowledge, the first clinical study on cineole as a monosubstance in the field of common cold treatment. Although our study design differs from that of a standard randomised controlled trial, our findings are in accordance with previous research results on cineole in other indications concerning the respiratory tract. Symptom improvement with cineole intake correlates well with previously reported benefits for the treatment of rhinosinusitis and acute bronchitis [18,19]. Tendency towards higher prevalence of acute bronchitis with later treatment initiation suggests a benefit of starting cineole intake when first symptoms of common cold occur and corresponds well to the previously described positive effects of cineole in the treatment of acute bronchitis [19,33]. In addition, faster recovery observed with acute bronchitis seems to extend to common cold, as our findings indicate corresponding benefits of early cineole treatment [33]. Altogether, the outlined efficacy results are in accordance with existing research indicating anti-inflammatory and antiviral properties of cineole [11,12,15,39–42]. Part of the immune response to rhinoviruses is the production of interferons which impair viral replication [43]. Cineole can potentiate poly(I:C)-induced activity of the antiviral transcription factor IRF3, while simultaneously reducing proinflammatory nuclear factor NF-κB activity as demonstrated in human cell lines, human nasal stem cells isolated from the inferior turbinate and in ex vivo cultivated human nasal mucosa [41]. Common cold is characterized by an overproduction of mucus, a typical symptom of inflammatory airway diseases, and cineole has been shown to reduce the production of mucus and expression of the NF-κB target gene MUC2 in human nasal slices ex vivo, associated with a reduced NF-κB-activity [16]. Favourable tolerability profile is particularly important for the common cold as a frequent, but mild and generally self-limiting condition [9]. In accordance with a previous study on the treatment of acute bronchitis with Soledum^®^, our findings also suggest good tolerability of this cineole-based treatment option [33].

Some of the efficacy endpoints in our trial present with findings which could on the first glance seem counterintuitive but can be explained in a broader context. Global judgement of efficacy was better with longer time to treatment, which is probably due to a stronger perception of symptom reduction in case of a stronger manifestation of common cold. Earlier treatment initiation being associated with a longer sick leave might be due to patients with earlier responsiveness to the illness being more cautious and therefore more prone to taking sick leave. In addition, patients with a later treatment start would not be able to have their sick leave retrospectively attested. Comparable number of bed rest days in the investigated strata might stem from the nature of the illness, with lighter symptoms in the early days of infection not being perceived as severe enough to warrant bed rest. Taken together, most efficacy endpoints of the trial including the reduced burden of symptoms, improved quality of life, shorter time to symptom relief, tendency toward lower prevalence of acute bronchitis and faster common cold remission suggest advantages of early cineole treatment initiation. Increased productivity and reduced burden on health care associated with reduction of common cold symptoms and duration might have wide-ranging societal benefits. In light of the significance of common cold for the exacerbation of asthma and COPD [4,9] and potential effects of faster healing on reduced transmission of viral infections, research into common cold treatment with cineole might carry positive implications for public health. Furthermore, curbing inappropriate antibiotic consumption through effective common cold treatment supports the fight against antimicrobial resistance. While establishing a common cold study design with a separate comparator will be relevant for future research, we are confident our current work offers insights that might help shape future studies on the kinetics of treatment efficacy in acute respiratory infections.

## Supporting information

S1 Table GLM model 3 for WURSS-11 AUC. (PDF)

S2 Table GLM model 1 for WURSS-11 AUC (without imputation). (PDF)

S3 Table MMRM model 2 for WURSS-11 mean daily symptom score. (PDF)

S4 Table MMRM model 1 for WURSS-11 mean daily total score (without imputation). (PDF)

S5 Table MMRM model 1 for WURSS-11 mean daily symptom score (without imputation). (PDF)

S6 Table MMRM model 1 for WURSS-11 mean daily QoL score (without imputation). (PDF)

S7 Table Occurrence of acute bronchitis during the trial. (PDF)

S8 Table WURSS-11 single items (descriptive statistics). (PDF)

S9 Table Symptom severity peak. (PDF)

S10 Table Time to symptom relief. (PDF)

S11 Table Investigator assessment of common cold symptoms. (PDF)

S12 Table Days of bed rest due to common cold. (PDF)

S13 Table Summary of reported adverse events. (PDF)

S1 File Source data tables. (DOCX)

S2 File Clinical trial protocol. (PDF)

S3 File TREND checklist. (PDF)
