# Supplementary material for: The impact of cineole treatment timing on common cold duration and symptoms: Non-randomized exploratory clinical trial
Source: PLoS One. 2024 Jan 18;19(1):e0296482. doi: 10.1371/journal.pone.0296482 (PMC10795983; doi:10.1371/journal.pone.0296482)
Supplement: S1 Table — (PDF) [file pone.0296482.s001.pdf]

S1 Table: GLM model 3 for WURSS-11 AUC

		AUC WURSS (imputed)			
	**Fixed factors**	**Stratum 1: <= 12 hours**	**Stratum 2: 12-24 hours**	**Stratum 3: > 24 hours**	**Type 3 two-sided p-values**
LS mean (95% CI)[a, c]		139.3 [113.3, 165.3]	180.5 [151.8, 209.2]	229.8 [202.0, 257.5]	
Difference between LS means (95% CI)[b, c]		-90.5 [-119.4, -61.6]	-49.3 [-80.5, -18.0]	N/A	
Type 3 effects[a]	Time to treatment stratum				<.0001
	Previous influenza vaccination				0.0008
	Alcohol consumption (cat.)				<.0001
	Working status				0.0085
	Baseline WURSS (cat.)				<.0001

[a] Model contains time-to-treatment strata, baseline total symptom score, vaccination status, working status, alcohol consumption.

[a] Non-overlapping confidence intervals of LS-Meams between 2 Strata indicate a significant difference between strata.
Generalized linear model used inverse probability weights derived from propensity scoring approach.

[b] Compared with initiation of therapy at > 24 hours after start of illness.

[b] If 0 is outside the confidence interval for the differences between LS-Means then this comparison is significant.

[c] N/A: not applicable, CI: confidence interval.
