# Supplementary material for: The impact of cineole treatment timing on common cold duration and symptoms: Non-randomized exploratory clinical trial
Source: PLoS One. 2024 Jan 18;19(1):e0296482. doi: 10.1371/journal.pone.0296482 (PMC10795983; doi:10.1371/journal.pone.0296482)
Supplement: S2 Table — (PDF) [file pone.0296482.s002.pdf]

S2 Table: GLM model 1 for WURSS-11 AUC (without imputation)

	Fixed factors	AUC WURSS (not imputed) Stratum 1: <= 12 hours	Stratum 2: 12-24 hours	Stratum 3: > 24 hours	Type 3 two-sided p-values
LS mean (95% CI)[a, c]		119.7 [98.6, 140.9]	164.0 [139.8, 188.2]	232.4 [208.6, 256.1]	
Difference between LS means (95% CI)[b, c]		-112.6 [-141.0, -84.3]	-68.4 [-98.9, -37.9]	N/A	
Type 3 effects[a]	Time to treatment stratum				<.0001
	Age				0.9898
	Sex				0.9170
	Previous influenza vaccination				0.0125
	Baseline WURSS Score				<.0001

[a] Model contains time-to-treatment strata, baseline total symptom score, sex, age, vaccination status.

[a] Non-overlapping confidence intervals of LS-Meams between 2 Strata indicate a significant difference between strata.

[b] Compared with initiation of therapy at > 24 hours after start of illness.

[b] If 0 is outside the confidence interval for the differences between LS-Means then this comparison is significant.

[c] N/A: not applicable, CI: confidence interval.
