# Supplementary material for: The impact of cineole treatment timing on common cold duration and symptoms: Non-randomized exploratory clinical trial
Source: PLoS One. 2024 Jan 18;19(1):e0296482. doi: 10.1371/journal.pone.0296482 (PMC10795983; doi:10.1371/journal.pone.0296482)
Supplement: S3 Table — (PDF) [file pone.0296482.s003.pdf]

S3 Table: MMRM model 2 for WURSS-11 mean daily symptom score

Effect	Symptom Day	LS-Means	95% CI	p-value
Alcohol consumption (cat.)	.	.		0.3568
Baseline WURSS (cat.)	.	.		<.0001
Previous influenza vaccination	.	.		0.0818
Working status	.	.		0.4303
Stratum <= 12 hours	1	21.59	[20.08, 23.09]	
	2	19.06	[17.53, 20.59]	
	3	15.69	[14.10, 17.28]	
	4	12.90	[11.26, 14.54]	
	5	10.28	[8.62, 11.94]	
	6	8.02	[6.33, 9.71]	
	7	6.05	[4.36, 7.74]	
	8	4.57	[2.94, 6.20]	
	9	3.34	[1.81, 4.87]	
	10	2.70	[1.26, 4.14]	
	11	2.06	[0.76, 3.36]	
	12	1.23	[0.09, 2.37]	
	13	0.84	[0.00, 1.90]	
	14	0.53	[0.00, 1.47]	
	15	0.26	[0.00, 1.13]	
	16	0.14	[0.00, 0.94]	
	17	0.09	[0.00, 0.90]	
Stratum 12-24 hours	1	22.95	[21.19, 24.71]	
	2	22.28	[20.49, 24.07]	
	3	20.42	[18.56, 22.29]	
	4	18.01	[16.09, 19.94]	
	5	15.03	[13.08, 16.97]	
	6	11.96	[9.98, 13.94]	
	7	9.26	[7.28, 11.24]	
	8	7.38	[5.47, 9.29]	
	9	5.45	[3.65, 7.25]	
	10	4.25	[2.57, 5.94]	
	11	2.68	[1.16, 4.19]	
	12	1.88	[0.56, 3.21]	
	13	1.38	[0.15, 2.62]	
	14	0.82	[0.00, 1.90]	
	15	0.36	[0.00, 1.36]	
	16	0.25	[0.00, 1.17]	
	17	0.15	[0.00, 1.08]	

Effect	Symptom Day	LS-Means	95% CI	p-value
Stratum >24 hours	1	22.43	[20.75, 24.11]	
	2	23.78	[22.08, 25.49]	
	3	23.03	[21.25, 24.81]	
	4	21.47	[19.64, 23.30]	
	5	19.27	[17.41, 21.12]	
	6	16.22	[14.34, 18.11]	
	7	13.95	[12.06, 15.84]	
	8	11.04	[9.22, 12.86]	
	9	8.57	[6.85, 10.28]	
	10	6.80	[5.19, 8.41]	
	11	5.57	[4.12, 7.02]	
	12	4.02	[2.74, 5.29]	
	13	3.34	[2.15, 4.53]	
	14	2.22	[1.17, 3.27]	
	15	1.50	[0.53, 2.47]	
	16	1.07	[0.17, 1.96]	
	17	1.03	[0.13, 1.93]	
Stratum <= 12 hours - Stratum 12-24 hours	1	-1.36	[-3.56, 0.84]	0.2238
	2	-3.22	[-5.46, -0.98]	0.0050 *
	3	-4.73	[-7.08, -2.38]	<.0001 *
	4	-5.12	[-7.54, -2.69]	<.0001 *
	5	-4.75	[-7.20, -2.29]	0.0002 *
	6	-3.94	[-6.44, -1.43]	0.0022 *
	7	-3.21	[-5.72, -0.70]	0.0123 *
	8	-2.81	[-5.22, -0.40]	0.0224 *
	9	-2.11	[-4.37, 0.14]	0.0662
	10	-1.56	[-3.66, 0.55]	0.1464
	11	-0.62	[-2.48, 1.25]	0.5148
	12	-0.65	[-2.25, 0.94]	0.4198
	13	-0.54	[-2.01, 0.93]	0.4684
	14	-0.28	[-1.53, 0.96]	0.6544
	15	-0.09	[-1.21, 1.02]	0.8706
	16	-0.11	[-1.10, 0.88]	0.8314
	17	-0.06	[-1.06, 0.94]	0.9067
Stratum <= 12 hours - Stratum >24 hours	1	-0.84	[-2.98, 1.29]	0.4376
	2	-4.72	[-6.90, -2.54]	<.0001 *
	3	-7.34	[-9.62, -5.05]	<.0001 *
	4	-8.57	[-10.93, -6.22]	<.0001 *
	5	-8.99	[-11.37, -6.60]	<.0001 *
	6	-8.20	[-10.64, -5.77]	<.0001 *
	7	-7.90	[-10.34, -5.47]	<.0001 *
	8	-6.47	[-8.81, -4.13]	<.0001 *
	9	-5.23	[-7.42, -3.04]	<.0001 *
	10	-4.10	[-6.14, -2.06]	<.0001 *
	11	-3.51	[-5.32, -1.70]	0.0002 *
	12	-2.79	[-4.34, -1.23]	0.0005 *
	13	-2.49	[-3.92, -1.07]	0.0007 *
	14	-1.68	[-2.90, -0.47]	0.0067 *
	15	-1.24	[-2.33, -0.15]	0.0264 *
	16	-0.92	[-1.89, 0.05]	0.0619
	17	-0.94	[-1.91, 0.04]	0.0603

Effect	Symptom Day	LS-Means	95% CI	p-value
Stratum 12-24 hours - Stratum >24 hours	1	0.52	[-1.80, 2.83]	0.6597
	2	-1.50	[-3.86, 0.86]	0.2113
	3	-2.60	[-5.07, -0.13]	0.0390 *
	4	-3.46	[-6.01, -0.90]	0.0081 *
	5	-4.24	[-6.83, -1.65]	0.0014 *
	6	-4.27	[-6.90, -1.63]	0.0016 *
	7	-4.69	[-7.33, -2.05]	0.0005 *
	8	-3.66	[-6.20, -1.13]	0.0047 *
	9	-3.12	[-5.49, -0.74]	0.0102 *
	10	-2.54	[-4.76, -0.33]	0.0244 *
	11	-2.89	[-4.86, -0.93]	0.0040 *
	12	-2.13	[-3.81, -0.45]	0.0132 *
	13	-1.95	[-3.50, -0.41]	0.0135 *
	14	-1.40	[-2.72, -0.08]	0.0371 *
	15	-1.14	[-2.33, 0.04]	0.0582
	16	-0.82	[-1.87, 0.24]	0.1284
	17	-0.88	[-1.94, 0.18]	0.1050

[c] If lower limit of 95% confidence interval of LSMEANS is below 0 then this value is set to 0. * = significant difference between LSMEANS.
