# Supplementary material for: The impact of cineole treatment timing on common cold duration and symptoms: Non-randomized exploratory clinical trial
Source: PLoS One. 2024 Jan 18;19(1):e0296482. doi: 10.1371/journal.pone.0296482 (PMC10795983; doi:10.1371/journal.pone.0296482)
Supplement: S4 Table — (PDF) [file pone.0296482.s004.pdf]

S4 Table: MMRM model 1 for WURSS-11 mean daily total score (without imputation)

Effect	Symptom Day	LS-Means	95% CI	p-value
Age	.	.		0.6922
Baseline WURSS Score	.	.		<.0001
Previous influenza vaccination	.	.		0.1608
Sex	.	.		0.1530
Stratum <= 12 hours	1	27.92	[26.40, 29.43]	
	2	25.11	[23.45, 26.76]	
	3	20.74	[18.90, 22.59]	
	4	17.03	[15.06, 18.99]	
	5	13.67	[11.68, 15.67]	
	6	10.72	[8.69, 12.74]	
	7	8.24	[6.18, 10.30]	
	8	6.37	[4.36, 8.37]	
	9	4.65	[2.77, 6.54]	
	10	3.85	[2.07, 5.63]	
	11	2.99	[1.40, 4.58]	
	12	1.84	[0.44, 3.23]	
	13	1.29	[0.00, 2.60]	
	14	0.84	[0.00, 2.01]	
	15	0.50	[0.00, 1.61]	
	16	0.22	[0.00, 1.23]	
	17	0.22	[0.00, 1.21]	
Stratum 12-24 hours	1	28.94	[27.16, 30.71]	
	2	28.68	[26.74, 30.62]	
	3	26.43	[24.27, 28.59]	
	4	23.27	[20.97, 25.57]	
	5	19.42	[17.09, 21.76]	
	6	15.42	[13.05, 17.79]	
	7	11.89	[9.47, 14.31]	
	8	9.49	[7.14, 11.84]	
	9	6.97	[4.76, 9.17]	
	10	5.45	[3.37, 7.53]	
	11	3.33	[1.48, 5.18]	
	12	2.20	[0.57, 3.82]	
	13	1.59	[0.07, 3.10]	
	14	0.90	[0.00, 2.25]	
	15	0.25	[0.00, 1.52]	
	16	0.00	[0.00, 1.03]	
	17	0.00	[0.00, 0.93]	

Effect	Symptom Day	LS-Means	95% CI	p-value
Stratum >24 hours	1	28.90	[27.20, 30.60]	
	2	30.82	[28.96, 32.67]	
	3	30.01	[27.95, 32.07]	
	4	28.06	[25.86, 30.25]	
	5	25.12	[22.89, 27.35]	
	6	21.23	[18.97, 23.49]	
	7	18.42	[16.11, 20.72]	
	8	14.68	[12.44, 16.91]	
	9	11.59	[9.49, 13.70]	
	10	9.41	[7.43, 11.40]	
	11	7.74	[5.97, 9.50]	
	12	5.79	[4.24, 7.35]	
	13	4.86	[3.40, 6.31]	
	14	3.37	[2.08, 4.66]	
	15	2.42	[1.20, 3.65]	
	16	1.67	[0.55, 2.79]	
	17	1.55	[0.46, 2.65]	
Stratum <= 12 hours - Stratum 12-24 hours	1	-1.02	[-3.31, 1.27]	0.3803
	2	-3.58	[-6.09, -1.07]	0.0054 *
	3	-5.69	[-8.49, -2.88]	<.0001 *
	4	-6.24	[-9.24, -3.25]	<.0001 *
	5	-5.75	[-8.79, -2.71]	0.0002 *
	6	-4.70	[-7.79, -1.62]	0.0029 *
	7	-3.65	[-6.80, -0.50]	0.0233 *
	8	-3.12	[-6.17, -0.07]	0.0451 *
	9	-2.31	[-5.18, 0.55]	0.1133
	10	-1.60	[-4.30, 1.10]	0.2456
	11	-0.34	[-2.74, 2.05]	0.7787
	12	-0.36	[-2.46, 1.74]	0.7363
	13	-0.30	[-2.25, 1.66]	0.7664
	14	-0.06	[-1.78, 1.66]	0.9449
	15	0.25	[-1.37, 1.87]	0.7624
	16	0.35	[-1.12, 1.83]	0.6367
	17	0.42	[-1.02, 1.86]	0.5671
Stratum <= 12 hours - Stratum >24 hours	1	-0.98	[-3.21, 1.24]	0.3840
	2	-5.71	[-8.15, -3.27]	<.0001 *
	3	-9.27	[-11.99, -6.55]	<.0001 *
	4	-11.03	[-13.93, -8.13]	<.0001 *
	5	-11.45	[-14.40, -8.50]	<.0001 *
	6	-10.51	[-13.50, -7.52]	<.0001 *
	7	-10.18	[-13.23, -7.12]	<.0001 *
	8	-8.31	[-11.27, -5.34]	<.0001 *
	9	-6.94	[-9.72, -4.16]	<.0001 *
	10	-5.56	[-8.18, -2.94]	<.0001 *
	11	-4.75	[-7.07, -2.42]	<.0001 *
	12	-3.96	[-5.99, -1.92]	0.0002 *
	13	-3.57	[-5.46, -1.67]	0.0003 *
	14	-2.53	[-4.20, -0.85]	0.0032 *
	15	-1.92	[-3.50, -0.35]	0.0170 *
	16	-1.45	[-2.88, -0.02]	0.0472 *
	17	-1.34	[-2.74, 0.06]	0.0606

Effect	Symptom Day	LS-Means	95% CI	p-value
Stratum 12-24 hours - Stratum >24 hours	1	0.04	[-2.37, 2.45]	0.9759
	2	-2.14	[-4.77, 0.50]	0.1120
	3	-3.58	[-6.52, -0.64]	0.0172 *
	4	-4.79	[-7.93, -1.64]	0.0029 *
	5	-5.70	[-8.89, -2.51]	0.0005 *
	6	-5.81	[-9.05, -2.57]	0.0005 *
	7	-6.53	[-9.83, -3.22]	0.0001 *
	8	-5.19	[-8.39, -1.98]	0.0016 *
	9	-4.63	[-7.63, -1.62]	0.0027 *
	10	-3.96	[-6.80, -1.13]	0.0062 *
	11	-4.40	[-6.92, -1.89]	0.0006 *
	12	-3.60	[-5.79, -1.40]	0.0014 *
	13	-3.27	[-5.32, -1.23]	0.0018 *
	14	-2.47	[-4.26, -0.67]	0.0074 *
	15	-2.17	[-3.86, -0.48]	0.0122 *
	16	-1.80	[-3.34, -0.26]	0.0219 *
	17	-1.76	[-3.26, -0.25]	0.0222 *

° If lower limit of 95% confidence interval of LSMEANS is below 0 then this value is set to 0. * = significant difference between LSMEANS.
