# Supplementary material for: The impact of cineole treatment timing on common cold duration and symptoms: Non-randomized exploratory clinical trial
Source: PLoS One. 2024 Jan 18;19(1):e0296482. doi: 10.1371/journal.pone.0296482 (PMC10795983; doi:10.1371/journal.pone.0296482)
Supplement: S5 Table — (PDF) [file pone.0296482.s005.pdf]

S5 Table: MMRM model 1 for WURSS-11 mean daily symptom score (without imputation)

Effect	Symptom Day	LS-Means	95% CI	p-value
Age	.	.		0.5607
Baseline symptom score	.	.		<.0001
Previous influenza vaccination	.	.		0.2914
Sex	.	.		0.0613
Stratum <= 12 hours	1	21.81	[20.56, 23.05]	
	2	19.28	[17.94, 20.62]	
	3	15.94	[14.49, 17.38]	
	4	13.12	[11.59, 14.64]	
	5	10.51	[8.96, 12.06]	
	6	8.24	[6.65, 9.83]	
	7	6.25	[4.64, 7.85]	
	8	4.80	[3.26, 6.35]	
	9	3.56	[2.10, 5.01]	
	10	2.91	[1.54, 4.27]	
	11	2.27	[1.04, 3.50]	
	12	1.43	[0.36, 2.51]	
	13	1.01	[0.00, 2.02]	
	14	0.65	[0.00, 1.54]	
	15	0.40	[0.00, 1.23]	
	16	0.16	[0.00, 0.90]	
	17	0.17	[0.00, 0.91]	
Stratum 12-24 hours	1	23.10	[21.63, 24.56]	
	2	22.43	[20.86, 24.00]	
	3	20.57	[18.87, 22.26]	
	4	18.18	[16.39, 19.97]	
	5	15.17	[13.35, 16.99]	
	6	12.04	[10.18, 13.90]	
	7	9.40	[7.52, 11.28]	
	8	7.52	[5.71, 9.34]	
	9	5.60	[3.89, 7.30]	
	10	4.41	[2.82, 6.01]	
	11	2.77	[1.34, 4.20]	
	12	1.92	[0.67, 3.16]	
	13	1.44	[0.28, 2.61]	
	14	0.87	[0.00, 1.89]	
	15	0.33	[0.00, 1.28]	
	16	0.00	[0.00, 0.83]	
	17	0.00	[0.00, 0.78]	

Effect	Symptom Day	LS-Means	95% CI	p-value
Stratum >24 hours	1	22.73	[21.33, 24.13]	
	2	24.16	[22.66, 25.66]	
	3	23.40	[21.78, 25.02]	
	4	21.87	[20.17, 23.58]	
	5	19.64	[17.91, 21.37]	
	6	16.60	[14.83, 18.37]	
	7	14.32	[12.53, 16.11]	
	8	11.41	[9.68, 13.14]	
	9	8.94	[7.32, 10.56]	
	10	7.17	[5.65, 8.69]	
	11	5.95	[4.59, 7.32]	
	12	4.38	[3.19, 5.58]	
	13	3.70	[2.58, 4.83]	
	14	2.58	[1.59, 3.57]	
	15	1.85	[0.93, 2.77]	
	16	1.31	[0.49, 2.13]	
	17	1.30	[0.47, 2.12]	
Stratum <= 12 hours - Stratum 12-24 hours	1	-1.29	[-3.18, 0.60]	0.1808
	2	-3.15	[-5.18, -1.11]	0.0026 *
	3	-4.63	[-6.83, -2.43]	<.0001 *
	4	-5.06	[-7.39, -2.73]	<.0001 *
	5	-4.66	[-7.03, -2.30]	0.0001 *
	6	-3.80	[-6.22, -1.38]	0.0022 *
	7	-3.16	[-5.60, -0.71]	0.0116 *
	8	-2.72	[-5.08, -0.36]	0.0241 *
	9	-2.04	[-4.25, 0.17]	0.0707
	10	-1.50	[-3.58, 0.57]	0.1544
	11	-0.50	[-2.35, 1.35]	0.5970
	12	-0.48	[-2.10, 1.13]	0.5541
	13	-0.44	[-1.94, 1.07]	0.5677
	14	-0.22	[-1.53, 1.09]	0.7429
	15	0.07	[-1.14, 1.29]	0.9072
	16	0.18	[-0.89, 1.25]	0.7366
	17	0.24	[-0.84, 1.32]	0.6631
Stratum <= 12 hours - Stratum >24 hours	1	-0.93	[-2.77, 0.91]	0.3211
	2	-4.87	[-6.85, -2.90]	<.0001 *
	3	-7.46	[-9.60, -5.33]	<.0001 *
	4	-8.76	[-11.02, -6.50]	<.0001 *
	5	-9.13	[-11.43, -6.84]	<.0001 *
	6	-8.36	[-10.71, -6.01]	<.0001 *
	7	-8.08	[-10.45, -5.70]	<.0001 *
	8	-6.61	[-8.90, -4.32]	<.0001 *
	9	-5.38	[-7.53, -3.23]	<.0001 *
	10	-4.26	[-6.27, -2.25]	<.0001 *
	11	-3.69	[-5.48, -1.89]	<.0001 *
	12	-2.95	[-4.52, -1.39]	0.0002 *
	13	-2.70	[-4.16, -1.24]	0.0003 *
	14	-1.93	[-3.21, -0.66]	0.0031 *
	15	-1.45	[-2.63, -0.27]	0.0160 *
	16	-1.15	[-2.19, -0.11]	0.0304 *
	17	-1.13	[-2.18, -0.08]	0.0344 *

Effect	Symptom Day	LS-Means	95% CI	p-value
Stratum 12-24 hours - Stratum >24 hours	1	0.36	[-1.63, 2.35]	0.7206
	2	-1.73	[-3.87, 0.41]	0.1124
	3	-2.83	[-5.14, -0.52]	0.0166 *
	4	-3.69	[-6.14, -1.25]	0.0032 *
	5	-4.47	[-6.95, -1.99]	0.0005 *
	6	-4.56	[-7.10, -2.02]	0.0005 *
	7	-4.92	[-7.49, -2.35]	0.0002 *
	8	-3.89	[-6.37, -1.41]	0.0022 *
	9	-3.34	[-5.66, -1.02]	0.0049 *
	10	-2.76	[-4.93, -0.58]	0.0131 *
	11	-3.19	[-5.13, -1.25]	0.0014 *
	12	-2.47	[-4.16, -0.78]	0.0043 *
	13	-2.26	[-3.83, -0.68]	0.0051 *
	14	-1.71	[-3.09, -0.34]	0.0147 *
	15	-1.52	[-2.79, -0.26]	0.0185 *
	16	-1.33	[-2.45, -0.22]	0.0197 *
	17	-1.37	[-2.50, -0.25]	0.0173 *

[c] If lower limit of 95% confidence interval of LSMEANS is below 0 then this value is set to 0. * = significant difference between LSMEANS.
