# Supplementary material for: The impact of cineole treatment timing on common cold duration and symptoms: Non-randomized exploratory clinical trial
Source: PLoS One. 2024 Jan 18;19(1):e0296482. doi: 10.1371/journal.pone.0296482 (PMC10795983; doi:10.1371/journal.pone.0296482)
Supplement: S6 Table — (PDF) [file pone.0296482.s006.pdf]

S6 Table: MMRM model 1 for WURSS-11 mean daily QoL score (without imputation)

Effect	Symptom Day	LS-Means	95% CI	p-value
Baseline QoL score	.	.		<.0001
Stratum <= 12 hours	1	6.15	[5.61, 6.68]	
	2	5.86	[5.34, 6.38]	
	3	4.84	[4.28, 5.40]	
	4	3.95	[3.38, 4.52]	
	5	3.20	[2.65, 3.76]	
	6	2.51	[1.98, 3.05]	
	7	2.03	[1.49, 2.57]	
	8	1.60	[1.08, 2.12]	
	9	1.13	[0.65, 1.62]	
	10	0.98	[0.51, 1.44]	
	11	0.76	[0.37, 1.14]	
	12	0.44	[0.11, 0.77]	
	13	0.32	[0.03, 0.61]	
	14	0.24	[0.00, 0.48]	
	15	0.15	[0.00, 0.38]	
	16	0.13	[0.00, 0.34]	
	17	0.10	[0.00, 0.27]	
Stratum 12-24 hours	1	6.07	[5.44, 6.70]	
	2	6.46	[5.85, 7.07]	
	3	6.06	[5.40, 6.72]	
	4	5.30	[4.63, 5.97]	
	5	4.45	[3.80, 5.11]	
	6	3.59	[2.96, 4.22]	
	7	2.69	[2.05, 3.32]	
	8	2.17	[1.56, 2.78]	
	9	1.57	[1.00, 2.14]	
	10	1.23	[0.69, 1.77]	
	11	0.76	[0.31, 1.22]	
	12	0.47	[0.09, 0.86]	
	13	0.34	[0.00, 0.68]	
	14	0.23	[0.00, 0.51]	
	15	0.12	[0.00, 0.39]	
	16	0.10	[0.00, 0.34]	
	17	0.07	[0.00, 0.26]	

Effect	Symptom Day	LS-Means	95% CI	p-value
Stratum >24 hours	1	6.22	[5.63, 6.82]	
	2	6.73	[6.15, 7.31]	
	3	6.68	[6.05, 7.30]	
	4	6.25	[5.62, 6.89]	
	5	5.55	[4.93, 6.17]	
	6	4.70	[4.10, 5.29]	
	7	4.16	[3.56, 4.76]	
	8	3.33	[2.75, 3.91]	
	9	2.72	[2.18, 3.26]	
	10	2.31	[1.80, 2.82]	
	11	1.85	[1.42, 2.28]	
	12	1.48	[1.11, 1.84]	
	13	1.22	[0.90, 1.54]	
	14	0.85	[0.59, 1.12]	
	15	0.63	[0.38, 0.89]	
	16	0.45	[0.22, 0.68]	
	17	0.34	[0.16, 0.52]	
Stratum <= 12 hours - Stratum 12-24 hours	1	0.07	[-0.75, 0.90]	0.8629
	2	-0.60	[-1.40, 0.20]	0.1419
	3	-1.22	[-2.09, -0.35]	0.0059 *
	4	-1.35	[-2.23, -0.47]	0.0027 *
	5	-1.25	[-2.11, -0.39]	0.0044 *
	6	-1.08	[-1.90, -0.25]	0.0106 *
	7	-0.65	[-1.49, 0.18]	0.1228
	8	-0.57	[-1.37, 0.23]	0.1609
	9	-0.44	[-1.19, 0.31]	0.2476
	10	-0.25	[-0.97, 0.46]	0.4824
	11	-0.01	[-0.61, 0.59]	0.9793
	12	-0.03	[-0.54, 0.48]	0.9035
	13	-0.02	[-0.46, 0.43]	0.9362
	14	0.01	[-0.36, 0.38]	0.9495
	15	0.03	[-0.33, 0.39]	0.8773
	16	0.03	[-0.29, 0.35]	0.8557
	17	0.04	[-0.22, 0.29]	0.7867
Stratum <= 12 hours - Stratum >24 hours	1	-0.08	[-0.88, 0.72]	0.8505
	2	-0.87	[-1.65, -0.10]	0.0279 *
	3	-1.84	[-2.68, -1.00]	<.0001 *
	4	-2.30	[-3.16, -1.45]	<.0001 *
	5	-2.35	[-3.18, -1.51]	<.0001 *
	6	-2.19	[-2.99, -1.39]	<.0001 *
	7	-2.13	[-2.94, -1.32]	<.0001 *
	8	-1.73	[-2.51, -0.96]	<.0001 *
	9	-1.59	[-2.32, -0.87]	<.0001 *
	10	-1.33	[-2.02, -0.64]	0.0002 *
	11	-1.10	[-1.68, -0.52]	0.0002 *
	12	-1.04	[-1.53, -0.54]	<.0001 *
	13	-0.90	[-1.33, -0.47]	<.0001 *
	14	-0.61	[-0.97, -0.25]	0.0009 *
	15	-0.48	[-0.83, -0.14]	0.0063 *
	16	-0.33	[-0.64, -0.02]	0.0398 *
	17	-0.24	[-0.49, 0.01]	0.0584

Effect	Symptom Day	LS-Means	95% CI	p-value
Stratum 12-24 hours - Stratum >24 hours	1	-0.15	[-1.02, 0.72]	0.7354
	2	-0.27	[-1.11, 0.57]	0.5238
	3	-0.62	[-1.52, 0.29]	0.1832
	4	-0.95	[-1.88, -0.03]	0.0428 *
	5	-1.10	[-2.00, -0.20]	0.0171 *
	6	-1.11	[-1.97, -0.24]	0.0123 *
	7	-1.48	[-2.35, -0.60]	0.0010 *
	8	-1.16	[-2.00, -0.32]	0.0068 *
	9	-1.15	[-1.94, -0.37]	0.0042 *
	10	-1.08	[-1.83, -0.33]	0.0047 *
	11	-1.09	[-1.72, -0.46]	0.0007 *
	12	-1.01	[-1.54, -0.47]	0.0002 *
	13	-0.88	[-1.35, -0.42]	0.0002 *
	14	-0.63	[-1.01, -0.24]	0.0017 *
	15	-0.51	[-0.88, -0.14]	0.0072 *
	16	-0.36	[-0.69, -0.02]	0.0370 *
	17	-0.27	[-0.54, -0.01]	0.0438 *

[c] If lower limit of 95% confidence interval of LSMEANS is below 0 then this value is set to 0. * = significant difference between LSMEANS. Model 1 did not converge. Therefore only baseline values were included into the model.
