# Supplementary material for: The impact of cineole treatment timing on common cold duration and symptoms: Non-randomized exploratory clinical trial
Source: PLoS One. 2024 Jan 18;19(1):e0296482. doi: 10.1371/journal.pone.0296482 (PMC10795983; doi:10.1371/journal.pone.0296482)
Supplement: S7 Table — (PDF) [file pone.0296482.s007.pdf]

S7 Table: Occurrence of acute bronchitis during the trial

	Time to treatment stratum						Total (N=308)	
	≤12 h (N=122)		>12 to 24 h (N=88)		>24 h (N=98)			
	n	%	n	%	n	%	n	%
**Presence of acute bronchitis**								
No[1]	79	64.8	53	60.2	52	53.1	184	59.7
Yes	40	32.8	35	39.8	45	45.9	120	39.0
Unknown	3	2.5	0	0	1	1.0	4	1.3
**Severity of acute bronchitis**								
Mild	32	26.2	28	31.8	38	38.8	98	31.8
Moderate	7	5.7	7	8.0	6	6.1	20	6.5
Severe	1	0.8	0	0	1	1.0	2	0.6
