# Supplementary material for: The impact of cineole treatment timing on common cold duration and symptoms: Non-randomized exploratory clinical trial
Source: PLoS One. 2024 Jan 18;19(1):e0296482. doi: 10.1371/journal.pone.0296482 (PMC10795983; doi:10.1371/journal.pone.0296482)
Supplement: S8 Table — (PDF) [file pone.0296482.s008.pdf]

S8 Table: WURSS-11 single items (descriptive statistics)

02 - Runny nose (daily mean) (imputed)		Stratum <= 12 hours (N=122)	Stratum 12 - 24 hours (N=88)	Stratum > 24 hours (N=98)	Total (N=308)
**Symptom day**					
**1**	N_{valid}	122	86	96	304
	$N_{missing}$	0	2	2	4
	**Mean**	3.47	3.15	3.30	3.32
	SD	1.67	1.85	1.87	1.78
	Minimum	0.0	0.0	0.0	0.0
	Median	3.50	3.50	3.00	3.50
	Maximum	7.0	7.0	7.0	7.0
**2**	N_{valid}	122	88	98	308
	$N_{missing}$	0	0	0	0
	**Mean**	3.05	3.13	3.70	3.28
	SD	1.74	1.69	1.75	1.75
	Minimum	0.0	0.0	0.0	0.0
	Median	3.00	3.50	4.00	3.50
	Maximum	7.0	7.0	7.0	7.0
**3**	N_{valid}	122	88	98	308
	$N_{missing}$	0	0	0	0
	**Mean**	2.66	3.04	3.59	3.07
	SD	1.73	1.75	1.83	1.81
	Minimum	0.0	0.0	0.0	0.0
	Median	3.00	3.50	4.00	3.50
	Maximum	7.0	7.0	7.0	7.0

02 - Runny nose (daily mean) (imputed)		Stratum <= 12 hours (N=122)	Stratum 12 - 24 hours (N=88)	Stratum > 24 hours (N=98)	Total (N=308)
**4**	N_{valid}	122	88	98	308
	$N_{missing}$	0	0	0	0
	**Mean**	2.15	2.82	3.28	2.70
	SD	1.63	1.88	1.80	1.82
	Minimum	0.0	0.0	0.0	0.0
	Median	2.00	3.00	3.50	3.00
	Maximum	7.0	7.0	6.0	7.0
**5**	N_{valid}	122	88	98	308
	$N_{missing}$	0	0	0	0
	**Mean**	1.69	2.35	2.99	2.29
	SD	1.46	1.71	1.84	1.74
	Minimum	0.0	0.0	0.0	0.0
	Median	1.50	2.00	3.00	2.00
	Maximum	6.0	6.5	6.0	6.5
**6**	N_{valid}	122	88	98	308
	$N_{missing}$	0	0	0	0
	**Mean**	1.31	1.98	2.50	1.88
	SD	1.34	1.56	1.84	1.65
	Minimum	0.0	0.0	0.0	0.0
	Median	1.00	2.00	2.50	1.50
	Maximum	7.0	5.0	6.0	7.0
**7**	N_{valid}	122	88	98	308
	$N_{missing}$	0	0	0	0
	**Mean**	1.00	1.56	2.06	1.49
	SD	1.30	1.55	1.84	1.62
	Minimum	0.0	0.0	0.0	0.0
	Median	0.50	1.00	2.00	1.00
	Maximum	5.0	5.0	6.0	6.0
**8**	N_{valid}	122	88	98	308
	$N_{missing}$	0	0	0	0
	**Mean**	0.73	1.23	1.70	1.18
	SD	1.18	1.48	1.71	1.50
	Minimum	0.0	0.0	0.0	0.0
	Median	0.00	0.75	1.00	0.50
	Maximum	5.0	5.0	6.0	6.0
**9**	N_{valid}	122	88	98	308
	$N_{missing}$	0	0	0	0
	**Mean**	0.55	0.89	1.36	0.91
	SD	1.02	1.39	1.63	1.38
	Minimum	0.0	0.0	0.0	0.0
	Median	0.00	0.00	1.00	0.00
	Maximum	3.5	5.0	6.0	6.0
**10**	N_{valid}	122	88	98	308
	$N_{missing}$	0	0	0	0
	**Mean**	0.44	0.70	1.13	0.74
	SD	0.90	1.21	1.54	1.25
	Minimum	0.0	0.0	0.0	0.0
	Median	0.00	0.00	0.25	0.00
	Maximum	4.0	4.5	6.0	6.0

02 - Runny nose (daily mean) (imputed)		Stratum <= 12 hours (N=122)	Stratum 12 - 24 hours (N=88)	Stratum > 24 hours (N=98)	Total (N=308)
**11**	N_{valid}	122	88	98	308
	$N_{missing}$	0	0	0	0
	**Mean**	0.35	0.47	0.90	0.56
	SD	0.77	0.89	1.48	1.10
	Minimum	0.0	0.0	0.0	0.0
	Median	0.00	0.00	0.00	0.00
	Maximum	3.0	3.5	6.0	6.0
**12**	N_{valid}	122	88	98	308
	$N_{missing}$	0	0	0	0
	**Mean**	0.19	0.35	0.62	0.37
	SD	0.56	0.78	1.31	0.93
	Minimum	0.0	0.0	0.0	0.0
	Median	0.00	0.00	0.00	0.00
	Maximum	2.5	3.0	6.0	6.0
**13**	N_{valid}	122	88	98	308
	$N_{missing}$	0	0	0	0
	**Mean**	0.16	0.28	0.54	0.31
	SD	0.53	0.69	1.18	0.85
	Minimum	0.0	0.0	0.0	0.0
	Median	0.00	0.00	0.00	0.00
	Maximum	3.0	3.0	5.5	5.5
**14**	N_{valid}	122	88	98	308
	$N_{missing}$	0	0	0	0
	**Mean**	0.11	0.19	0.35	0.21
	SD	0.40	0.50	0.97	0.67
	Minimum	0.0	0.0	0.0	0.0
	Median	0.00	0.00	0.00	0.00
	Maximum	3.0	2.0	5.0	5.0
**15**	N_{valid}	122	88	98	308
	$N_{missing}$	0	0	0	0
	**Mean**	0.06	0.11	0.25	0.14
	SD	0.33	0.35	0.93	0.60
	Minimum	0.0	0.0	0.0	0.0
	Median	0.00	0.00	0.00	0.00
	Maximum	3.0	1.5	5.0	5.0
**16**	N_{valid}	122	88	98	308
	$N_{missing}$	0	0	0	0
	**Mean**	0.06	0.10	0.20	0.12
	SD	0.28	0.32	0.84	0.53
	Minimum	0.0	0.0	0.0	0.0
	Median	0.00	0.00	0.00	0.00
	Maximum	2.0	1.5	6.0	6.0
**17**	N_{valid}	122	88	98	308
	$N_{missing}$	0	0	0	0
	**Mean**	0.06	0.10	0.21	0.12
	SD	0.28	0.34	0.91	0.57
	Minimum	0.0	0.0	0.0	0.0
	Median	0.00	0.00	0.00	0.00
	Maximum	2.0	1.5	6.0	6.0

03 - Plugged nose (daily mean) (imputed)		Stratum <= 12 hours (N=122)	Stratum 12 - 24 hours (N=88)	Stratum > 24 hours (N=98)	Total (N=308)
**Symptom day**					
**1**	N_{valid}	122	86	96	304
	$N_{missing}$	0	2	2	4
	**Mean**	3.71	3.87	3.47	3.68
	SD	1.62	1.77	1.88	1.75
	Minimum	0.0	0.0	0.0	0.0
	Median	4.00	4.00	4.00	4.00
	Maximum	7.0	7.0	7.0	7.0
**2**	N_{valid}	122	88	98	308
	$N_{missing}$	0	0	0	0
	**Mean**	3.36	3.86	3.69	3.61
	SD	1.69	1.67	1.72	1.70
	Minimum	0.0	0.0	0.0	0.0
	Median	3.50	4.00	4.00	4.00
	Maximum	7.0	7.0	7.0	7.0
**3**	N_{valid}	122	88	98	308
	$N_{missing}$	0	0	0	0
	**Mean**	2.82	3.65	3.45	3.26
	SD	1.65	1.59	1.73	1.69
	Minimum	0.0	0.5	0.0	0.0
	Median	3.00	4.00	3.50	3.50
	Maximum	6.0	7.0	6.5	7.0
**4**	N_{valid}	122	88	98	308
	$N_{missing}$	0	0	0	0
	**Mean**	2.32	3.05	3.31	2.84
	SD	1.67	1.55	1.69	1.69
	Minimum	0.0	0.0	0.0	0.0
	Median	2.00	3.00	3.25	3.00
	Maximum	6.0	7.0	7.0	7.0
**5**	N_{valid}	122	88	98	308
	$N_{missing}$	0	0	0	0
	**Mean**	1.86	2.73	2.98	2.47
	SD	1.59	1.59	1.79	1.73
	Minimum	0.0	0.0	0.0	0.0
	Median	1.50	2.75	3.00	2.50
	Maximum	6.0	6.5	6.0	6.5
**6**	N_{valid}	122	88	98	308
	$N_{missing}$	0	0	0	0
	**Mean**	1.53	2.14	2.59	2.04
	SD	1.51	1.45	1.87	1.68
	Minimum	0.0	0.0	0.0	0.0
	Median	1.00	2.00	2.50	1.50
	Maximum	5.5	6.0	6.0	6.0
**7**	N_{valid}	122	88	98	308
	$N_{missing}$	0	0	0	0
	**Mean**	1.14	1.75	2.18	1.65
	SD	1.43	1.54	1.86	1.66
	Minimum	0.0	0.0	0.0	0.0
	Median	0.50	1.50	2.50	1.00
	Maximum	6.0	5.0	7.0	7.0

03 - Plugged nose (daily mean) (imputed)		Stratum <= 12 hours (N=122)	Stratum 12 - 24 hours (N=88)	Stratum > 24 hours (N=98)	Total (N=308)
**8**	N_{valid}	122	88	98	308
	$N_{missing}$	0	0	0	0
	**Mean**	0.86	1.42	1.74	1.30
	SD	1.34	1.54	1.73	1.57
	Minimum	0.0	0.0	0.0	0.0
	Median	0.00	1.00	1.50	0.50
	Maximum	5.5	5.0	7.0	7.0
**9**	N_{valid}	122	88	98	308
	$N_{missing}$	0	0	0	0
	**Mean**	0.71	1.06	1.41	1.04
	SD	1.23	1.47	1.64	1.47
	Minimum	0.0	0.0	0.0	0.0
	Median	0.00	0.00	1.00	0.00
	Maximum	5.0	5.0	7.0	7.0
**10**	N_{valid}	122	88	98	308
	$N_{missing}$	0	0	0	0
	**Mean**	0.53	0.86	1.13	0.82
	SD	1.11	1.35	1.56	1.35
	Minimum	0.0	0.0	0.0	0.0
	Median	0.00	0.00	0.00	0.00
	Maximum	5.0	5.0	7.0	7.0
**11**	N_{valid}	122	88	98	308
	$N_{missing}$	0	0	0	0
	**Mean**	0.40	0.60	0.90	0.62
	SD	0.95	1.03	1.49	1.18
	Minimum	0.0	0.0	0.0	0.0
	Median	0.00	0.00	0.00	0.00
	Maximum	5.0	4.0	7.0	7.0
**12**	N_{valid}	122	88	98	308
	$N_{missing}$	0	0	0	0
	**Mean**	0.28	0.56	0.68	0.49
	SD	0.74	1.11	1.34	1.08
	Minimum	0.0	0.0	0.0	0.0
	Median	0.00	0.00	0.00	0.00
	Maximum	4.0	5.0	7.0	7.0
**13**	N_{valid}	122	88	98	308
	$N_{missing}$	0	0	0	0
	**Mean**	0.24	0.39	0.58	0.39
	SD	0.71	0.88	1.27	0.97
	Minimum	0.0	0.0	0.0	0.0
	Median	0.00	0.00	0.00	0.00
	Maximum	4.0	4.0	7.0	7.0
**14**	N_{valid}	122	88	98	308
	$N_{missing}$	0	0	0	0
	**Mean**	0.18	0.30	0.44	0.29
	SD	0.63	0.72	1.10	0.84
	Minimum	0.0	0.0	0.0	0.0
	Median	0.00	0.00	0.00	0.00
	Maximum	3.5	4.0	5.5	5.5

03 - Plugged nose (daily mean) (imputed)		Stratum <= 12 hours (N=122)	Stratum 12 - 24 hours (N=88)	Stratum > 24 hours (N=98)	Total (N=308)
**15**	N_{valid}	122	88	98	308
	$N_{missing}$	0	0	0	0
	**Mean**	0.18	0.21	0.34	0.24
	SD	0.66	0.62	1.03	0.79
	Minimum	0.0	0.0	0.0	0.0
	Median	0.00	0.00	0.00	0.00
	Maximum	4.0	4.0	5.0	5.0
**16**	N_{valid}	122	88	98	308
	$N_{missing}$	0	0	0	0
	**Mean**	0.15	0.19	0.25	0.19
	SD	0.57	0.61	0.97	0.73
	Minimum	0.0	0.0	0.0	0.0
	Median	0.00	0.00	0.00	0.00
	Maximum	3.5	4.0	5.5	5.5
**17**	N_{valid}	122	88	98	308
	$N_{missing}$	0	0	0	0
	**Mean**	0.15	0.16	0.24	0.18
	SD	0.57	0.58	0.97	0.72
	Minimum	0.0	0.0	0.0	0.0
	Median	0.00	0.00	0.00	0.00
	Maximum	3.5	4.0	5.0	5.0

04 - Sneezing (daily mean) (imputed)		Stratum <= 12 hours (N=122)	Stratum 12 - 24 hours (N=88)	Stratum > 24 hours (N=98)	Total (N=308)
**Symptom day**					
**1**	N_{valid}	122	86	96	304
	$N_{missing}$	0	2	2	4
	**Mean**	2.79	3.28	2.94	2.98
	SD	1.67	1.77	1.76	1.73
	Minimum	0.0	0.0	0.0	0.0
	Median	3.00	3.50	3.00	3.00
	Maximum	7.0	6.0	7.0	7.0
**2**	N_{valid}	122	88	98	308
	$N_{missing}$	0	0	0	0
	**Mean**	2.29	2.91	3.07	2.71
	SD	1.55	1.72	1.56	1.64
	Minimum	0.0	0.0	0.0	0.0
	Median	2.50	3.00	3.00	3.00
	Maximum	6.5	6.0	6.0	6.5
**3**	N_{valid}	122	88	98	308
	$N_{missing}$	0	0	0	0
	**Mean**	1.88	2.59	2.82	2.38
	SD	1.52	1.72	1.69	1.68
	Minimum	0.0	0.0	0.0	0.0
	Median	1.50	2.50	3.00	2.50
	Maximum	5.5	6.0	6.0	6.0

04 - Sneezing (daily mean) (imputed)		Stratum <= 12 hours (N=122)	Stratum 12 - 24 hours (N=88)	Stratum > 24 hours (N=98)	Total (N=308)
**4**	N_{valid}	122	88	98	308
	$N_{missing}$	0	0	0	0
	**Mean**	1.54	2.38	2.70	2.15
	SD	1.47	1.65	1.73	1.68
	Minimum	0.0	0.0	0.0	0.0
	Median	1.00	2.50	2.50	2.00
	Maximum	5.0	6.0	6.0	6.0
**5**	N_{valid}	122	88	98	308
	$N_{missing}$	0	0	0	0
	**Mean**	1.22	1.91	2.44	1.81
	SD	1.30	1.59	1.79	1.63
	Minimum	0.0	0.0	0.0	0.0
	Median	1.00	1.75	2.50	1.50
	Maximum	5.0	5.5	6.0	6.0
**6**	N_{valid}	122	88	98	308
	$N_{missing}$	0	0	0	0
	**Mean**	0.93	1.51	2.02	1.44
	SD	1.26	1.42	1.83	1.57
	Minimum	0.0	0.0	0.0	0.0
	Median	0.50	1.00	1.50	1.00
	Maximum	4.5	5.0	6.0	6.0
**7**	N_{valid}	122	88	98	308
	$N_{missing}$	0	0	0	0
	**Mean**	0.66	1.17	1.77	1.16
	SD	1.07	1.41	1.76	1.49
	Minimum	0.0	0.0	0.0	0.0
	Median	0.00	0.50	1.00	0.50
	Maximum	4.5	5.0	6.0	6.0
**8**	N_{valid}	122	88	98	308
	$N_{missing}$	0	0	0	0
	**Mean**	0.50	0.91	1.42	0.91
	SD	0.94	1.34	1.62	1.36
	Minimum	0.0	0.0	0.0	0.0
	Median	0.00	0.00	1.00	0.00
	Maximum	4.0	5.0	6.0	6.0
**9**	N_{valid}	122	88	98	308
	$N_{missing}$	0	0	0	0
	**Mean**	0.38	0.73	1.12	0.72
	SD	0.93	1.30	1.52	1.28
	Minimum	0.0	0.0	0.0	0.0
	Median	0.00	0.00	0.00	0.00
	Maximum	5.0	5.0	6.0	6.0
**10**	N_{valid}	122	88	98	308
	$N_{missing}$	0	0	0	0
	**Mean**	0.32	0.56	0.95	0.59
	SD	0.78	1.12	1.44	1.15
	Minimum	0.0	0.0	0.0	0.0
	Median	0.00	0.00	0.00	0.00
	Maximum	3.7	4.5	6.0	6.0

04 - Sneezing (daily mean) (imputed)		Stratum <= 12 hours (N=122)	Stratum 12 - 24 hours (N=88)	Stratum > 24 hours (N=98)	Total (N=308)
**11**	N_{valid}	122	88	98	308
	$N_{missing}$	0	0	0	0
	**Mean**	0.23	0.33	0.81	0.44
	SD	0.63	0.74	1.41	1.00
	Minimum	0.0	0.0	0.0	0.0
	Median	0.00	0.00	0.00	0.00
	Maximum	3.0	3.5	6.0	6.0
**12**	N_{valid}	122	88	98	308
	$N_{missing}$	0	0	0	0
	**Mean**	0.18	0.23	0.53	0.30
	SD	0.58	0.62	1.09	0.80
	Minimum	0.0	0.0	0.0	0.0
	Median	0.00	0.00	0.00	0.00
	Maximum	3.5	3.0	5.5	5.5
**13**	N_{valid}	122	88	98	308
	$N_{missing}$	0	0	0	0
	**Mean**	0.09	0.22	0.45	0.25
	SD	0.32	0.62	1.04	0.72
	Minimum	0.0	0.0	0.0	0.0
	Median	0.00	0.00	0.00	0.00
	Maximum	2.0	3.0	5.0	5.0
**14**	N_{valid}	122	88	98	308
	$N_{missing}$	0	0	0	0
	**Mean**	0.07	0.15	0.31	0.17
	SD	0.36	0.47	0.84	0.59
	Minimum	0.0	0.0	0.0	0.0
	Median	0.00	0.00	0.00	0.00
	Maximum	3.5	2.5	4.0	4.0
**15**	N_{valid}	122	88	98	308
	$N_{missing}$	0	0	0	0
	**Mean**	0.04	0.07	0.24	0.11
	SD	0.27	0.29	0.93	0.57
	Minimum	0.0	0.0	0.0	0.0
	Median	0.00	0.00	0.00	0.00
	Maximum	2.5	1.5	5.0	5.0
**16**	N_{valid}	122	88	98	308
	$N_{missing}$	0	0	0	0
	**Mean**	0.04	0.05	0.14	0.07
	SD	0.27	0.24	0.62	0.41
	Minimum	0.0	0.0	0.0	0.0
	Median	0.00	0.00	0.00	0.00
	Maximum	2.5	1.5	3.5	3.5
**17**	N_{valid}	122	88	98	308
	$N_{missing}$	0	0	0	0
	**Mean**	0.04	0.05	0.15	0.08
	SD	0.27	0.24	0.65	0.43
	Minimum	0.0	0.0	0.0	0.0
	Median	0.00	0.00	0.00	0.00
	Maximum	2.5	1.5	4.0	4.0

05 - Sore throat (daily mean) (imputed)		Stratum <= 12 hours (N=122)	Stratum 12 - 24 hours (N=88)	Stratum > 24 hours (N=98)	Total (N=308)
**Symptom day**					
**1**	N_{valid}	122	86	96	304
	$N_{missing}$	0	2	2	4
	**Mean**	3.01	3.49	3.29	3.23
	SD	1.91	2.10	1.64	1.89
	Minimum	0.0	0.0	0.0	0.0
	Median	3.00	4.00	3.50	3.50
	Maximum	7.0	7.0	7.0	7.0
**2**	N_{valid}	122	88	98	308
	$N_{missing}$	0	0	0	0
	**Mean**	2.50	3.32	3.45	3.04
	SD	1.82	1.95	1.66	1.85
	Minimum	0.0	0.0	0.0	0.0
	Median	2.50	3.50	3.50	3.00
	Maximum	7.0	7.0	7.0	7.0
**3**	N_{valid}	122	88	98	308
	$N_{missing}$	0	0	0	0
	**Mean**	1.89	2.95	3.33	2.65
	SD	1.63	1.84	1.68	1.82
	Minimum	0.0	0.0	0.0	0.0
	Median	1.50	3.00	3.25	3.00
	Maximum	6.0	7.0	7.0	7.0
**4**	N_{valid}	122	88	98	308
	$N_{missing}$	0	0	0	0
	**Mean**	1.55	2.56	3.05	2.32
	SD	1.63	1.80	1.77	1.84
	Minimum	0.0	0.0	0.0	0.0
	Median	1.00	3.00	3.00	2.00
	Maximum	6.0	6.0	7.0	7.0
**5**	N_{valid}	122	88	98	308
	$N_{missing}$	0	0	0	0
	**Mean**	1.23	2.02	2.73	1.94
	SD	1.55	1.67	1.77	1.77
	Minimum	0.0	0.0	0.0	0.0
	Median	0.50	2.00	3.00	1.50
	Maximum	5.5	5.0	6.5	6.5
**6**	N_{valid}	122	88	98	308
	$N_{missing}$	0	0	0	0
	**Mean**	0.94	1.71	2.35	1.61
	SD	1.36	1.67	1.87	1.73
	Minimum	0.0	0.0	0.0	0.0
	Median	0.00	1.50	2.50	1.00
	Maximum	5.0	5.5	6.0	6.0
**7**	N_{valid}	122	88	98	308
	$N_{missing}$	0	0	0	0
	**Mean**	0.77	1.27	1.97	1.30
	SD	1.30	1.54	1.80	1.62
	Minimum	0.0	0.0	0.0	0.0
	Median	0.00	0.75	1.50	0.50
	Maximum	6.0	5.0	6.0	6.0

05 - Sore throat (daily mean) (imputed)		Stratum <= 12 hours (N=122)	Stratum 12 - 24 hours (N=88)	Stratum > 24 hours (N=98)	Total (N=308)
8	N_{valid}	122	88	98	308
	$N_{missing}$	0	0	0	0
	**Mean**	0.55	0.99	1.58	1.00
	SD	1.14	1.51	1.63	1.48
	Minimum	0.0	0.0	0.0	0.0
	Median	0.00	0.00	1.00	0.00
	Maximum	4.5	6.0	5.0	6.0
9	N_{valid}	122	88	98	308
	$N_{missing}$	0	0	0	0
	**Mean**	0.42	0.77	1.13	0.75
	SD	1.00	1.43	1.46	1.32
	Minimum	0.0	0.0	0.0	0.0
	Median	0.00	0.00	0.50	0.00
	Maximum	4.0	5.5	5.0	5.5
10	N_{valid}	122	88	98	308
	$N_{missing}$	0	0	0	0
	**Mean**	0.36	0.61	0.88	0.59
	SD	0.89	1.27	1.44	1.22
	Minimum	0.0	0.0	0.0	0.0
	Median	0.00	0.00	0.00	0.00
	Maximum	4.3	4.5	5.0	5.0
11	N_{valid}	122	88	98	308
	$N_{missing}$	0	0	0	0
	**Mean**	0.29	0.38	0.75	0.46
	SD	0.79	0.96	1.33	1.05
	Minimum	0.0	0.0	0.0	0.0
	Median	0.00	0.00	0.00	0.00
	Maximum	4.0	4.0	5.0	5.0
12	N_{valid}	122	88	98	308
	$N_{missing}$	0	0	0	0
	**Mean**	0.22	0.28	0.53	0.34
	SD	0.70	0.84	1.13	0.90
	Minimum	0.0	0.0	0.0	0.0
	Median	0.00	0.00	0.00	0.00
	Maximum	5.0	4.0	5.0	5.0
13	N_{valid}	122	88	98	308
	$N_{missing}$	0	0	0	0
	**Mean**	0.12	0.20	0.46	0.25
	SD	0.45	0.67	1.01	0.74
	Minimum	0.0	0.0	0.0	0.0
	Median	0.00	0.00	0.00	0.00
	Maximum	3.0	3.5	5.0	5.0
14	N_{valid}	122	88	98	308
	$N_{missing}$	0	0	0	0
	**Mean**	0.11	0.16	0.28	0.18
	SD	0.47	0.55	0.76	0.60
	Minimum	0.0	0.0	0.0	0.0
	Median	0.00	0.00	0.00	0.00
	Maximum	3.5	3.0	4.0	4.0

05 - Sore throat (daily mean) (imputed)		Stratum <= 12 hours (N=122)	Stratum 12 - 24 hours (N=88)	Stratum > 24 hours (N=98)	Total (N=308)
15	N_{valid}	122	88	98	308
	$N_{missing}$	0	0	0	0
	**Mean**	0.07	0.09	0.18	0.11
	SD	0.33	0.41	0.67	0.48
	Minimum	0.0	0.0	0.0	0.0
	Median	0.00	0.00	0.00	0.00
	Maximum	2.5	3.0	3.5	3.5
16	N_{valid}	122	88	98	308
	$N_{missing}$	0	0	0	0
	**Mean**	0.07	0.09	0.12	0.09
	SD	0.27	0.40	0.52	0.40
	Minimum	0.0	0.0	0.0	0.0
	Median	0.00	0.00	0.00	0.00
	Maximum	1.5	3.0	3.0	3.0
17	N_{valid}	122	88	98	308
	$N_{missing}$	0	0	0	0
	**Mean**	0.07	0.09	0.14	0.09
	SD	0.27	0.40	0.59	0.43
	Minimum	0.0	0.0	0.0	0.0
	Median	0.00	0.00	0.00	0.00
	Maximum	1.5	3.0	3.0	3.0

06 - Scratchy throat (daily mean) (imputed)		Stratum <= 12 hours (N=122)	Stratum 12 - 24 hours (N=88)	Stratum > 24 hours (N=98)	Total (N=308)
**Symptom day**					
1	N_{valid}	122	86	96	304
	$N_{missing}$	0	2	2	4
	**Mean**	2.80	3.57	2.95	3.06
	SD	1.78	1.90	1.76	1.83
	Minimum	0.0	0.0	0.0	0.0
	Median	2.50	3.75	3.00	3.00
	Maximum	6.0	7.0	7.0	7.0
2	N_{valid}	122	88	98	308
	$N_{missing}$	0	0	0	0
	**Mean**	2.58	3.43	3.16	3.01
	SD	1.76	1.69	1.64	1.73
	Minimum	0.0	0.0	0.0	0.0
	Median	2.75	3.50	3.50	3.00
	Maximum	7.0	7.0	7.0	7.0
3	N_{valid}	122	88	98	308
	$N_{missing}$	0	0	0	0
	**Mean**	2.00	3.05	3.09	2.65
	SD	1.65	1.67	1.74	1.76
	Minimum	0.0	0.0	0.0	0.0
	Median	2.00	3.50	3.00	3.00
	Maximum	6.0	7.0	6.5	7.0

06 - Scratchy throat (daily mean) (imputed)		Stratum <= 12 hours (N=122)	Stratum 12 - 24 hours (N=88)	Stratum > 24 hours (N=98)	Total (N=308)
**4**	N_{valid}	122	88	98	308
	$N_{missing}$	0	0	0	0
	**Mean**	1.66	2.68	2.87	2.34
	SD	1.59	1.66	1.72	1.74
	Minimum	0.0	0.0	0.0	0.0
	Median	1.50	3.00	3.00	2.25
	Maximum	6.0	6.0	6.5	6.5
**5**	N_{valid}	122	88	98	308
	$N_{missing}$	0	0	0	0
	**Mean**	1.23	2.21	2.51	1.92
	SD	1.45	1.59	1.75	1.69
	Minimum	0.0	0.0	0.0	0.0
	Median	0.75	2.00	2.50	1.50
	Maximum	5.0	5.0	6.5	6.5
**6**	N_{valid}	122	88	98	308
	$N_{missing}$	0	0	0	0
	**Mean**	1.00	1.70	2.17	1.57
	SD	1.37	1.51	1.87	1.65
	Minimum	0.0	0.0	0.0	0.0
	Median	0.50	1.50	2.00	1.00
	Maximum	5.0	5.0	6.0	6.0
**7**	N_{valid}	122	88	98	308
	$N_{missing}$	0	0	0	0
	**Mean**	0.80	1.35	1.89	1.30
	SD	1.32	1.41	1.79	1.57
	Minimum	0.0	0.0	0.0	0.0
	Median	0.00	1.00	1.50	0.50
	Maximum	5.5	5.0	6.0	6.0
**8**	N_{valid}	122	88	98	308
	$N_{missing}$	0	0	0	0
	**Mean**	0.58	1.12	1.44	1.01
	SD	1.14	1.44	1.62	1.44
	Minimum	0.0	0.0	0.0	0.0
	Median	0.00	0.50	1.00	0.00
	Maximum	4.5	5.0	5.0	5.0
**9**	N_{valid}	122	88	98	308
	$N_{missing}$	0	0	0	0
	**Mean**	0.41	0.81	1.13	0.75
	SD	0.92	1.32	1.48	1.27
	Minimum	0.0	0.0	0.0	0.0
	Median	0.00	0.00	0.50	0.00
	Maximum	4.0	5.0	5.0	5.0
**10**	N_{valid}	122	88	98	308
	$N_{missing}$	0	0	0	0
	**Mean**	0.35	0.65	0.83	0.59
	SD	0.87	1.26	1.38	1.18
	Minimum	0.0	0.0	0.0	0.0
	Median	0.00	0.00	0.00	0.00
	Maximum	4.3	5.0	5.0	5.0

06 - Scratchy throat (daily mean) (imputed)		Stratum <= 12 hours (N=122)	Stratum 12 - 24 hours (N=88)	Stratum > 24 hours (N=98)	Total (N=308)
**11**	N_{valid}	122	88	98	308
	$N_{missing}$	0	0	0	0
	**Mean**	0.30	0.40	0.71	0.46
	SD	0.83	0.87	1.29	1.02
	Minimum	0.0	0.0	0.0	0.0
	Median	0.00	0.00	0.00	0.00
	Maximum	5.0	4.0	5.0	5.0
**12**	N_{valid}	122	88	98	308
	$N_{missing}$	0	0	0	0
	**Mean**	0.18	0.27	0.54	0.32
	SD	0.55	0.71	1.05	0.80
	Minimum	0.0	0.0	0.0	0.0
	Median	0.00	0.00	0.00	0.00
	Maximum	4.0	4.0	4.5	4.5
**13**	N_{valid}	122	88	98	308
	$N_{missing}$	0	0	0	0
	**Mean**	0.14	0.20	0.42	0.25
	SD	0.53	0.68	0.97	0.74
	Minimum	0.0	0.0	0.0	0.0
	Median	0.00	0.00	0.00	0.00
	Maximum	4.0	4.0	4.0	4.0
**14**	N_{valid}	122	88	98	308
	$N_{missing}$	0	0	0	0
	**Mean**	0.11	0.15	0.26	0.17
	SD	0.52	0.52	0.83	0.64
	Minimum	0.0	0.0	0.0	0.0
	Median	0.00	0.00	0.00	0.00
	Maximum	4.5	3.0	4.5	4.5
**15**	N_{valid}	122	88	98	308
	$N_{missing}$	0	0	0	0
	**Mean**	0.07	0.10	0.22	0.13
	SD	0.39	0.43	0.71	0.53
	Minimum	0.0	0.0	0.0	0.0
	Median	0.00	0.00	0.00	0.00
	Maximum	3.5	3.0	3.5	3.5
**16**	N_{valid}	122	88	98	308
	$N_{missing}$	0	0	0	0
	**Mean**	0.07	0.10	0.15	0.10
	SD	0.36	0.43	0.59	0.46
	Minimum	0.0	0.0	0.0	0.0
	Median	0.00	0.00	0.00	0.00
	Maximum	3.5	3.0	3.0	3.5
**17**	N_{valid}	122	88	98	308
	$N_{missing}$	0	0	0	0
	**Mean**	0.07	0.10	0.12	0.09
	SD	0.36	0.43	0.52	0.43
	Minimum	0.0	0.0	0.0	0.0
	Median	0.00	0.00	0.00	0.00
	Maximum	3.5	3.0	3.0	3.5

Symptom day	07 - Cough (daily mean) (imputed)	Stratum <= 12 hours (N=122)	Stratum 12 - 24 hours (N=88)	Stratum > 24 hours (N=98)	Total (N=308)
1	N_{valid}	122	86	96	304
	$N_{missing}$	0	2	2	4
	**Mean**	2.00	2.10	2.51	2.19
	SD	1.93	1.95	1.72	1.88
	Minimum	0.0	0.0	0.0	0.0
	Median	1.50	2.00	2.75	2.00
	Maximum	7.0	6.5	6.0	7.0
2	N_{valid}	122	88	98	308
	$N_{missing}$	0	0	0	0
	**Mean**	1.86	2.02	2.81	2.21
	SD	1.81	1.93	1.72	1.86
	Minimum	0.0	0.0	0.0	0.0
	Median	1.50	1.50	3.00	2.00
	Maximum	6.0	6.0	6.0	6.0
3	N_{valid}	122	88	98	308
	$N_{missing}$	0	0	0	0
	**Mean**	1.70	2.03	2.98	2.20
	SD	1.81	1.87	1.85	1.92
	Minimum	0.0	0.0	0.0	0.0
	Median	1.00	2.00	3.00	2.00
	Maximum	6.5	5.5	7.0	7.0
4	N_{valid}	122	88	98	308
	$N_{missing}$	0	0	0	0
	**Mean**	1.45	1.91	2.80	2.01
	SD	1.71	1.94	1.79	1.89
	Minimum	0.0	0.0	0.0	0.0
	Median	1.00	1.50	3.00	1.50
	Maximum	7.0	6.0	6.5	7.0
5	N_{valid}	122	88	98	308
	$N_{missing}$	0	0	0	0
	**Mean**	1.24	1.68	2.51	1.77
	SD	1.51	1.84	1.89	1.81
	Minimum	0.0	0.0	0.0	0.0
	Median	0.50	1.25	2.75	1.00
	Maximum	5.0	6.0	7.0	7.0
6	N_{valid}	122	88	98	308
	$N_{missing}$	0	0	0	0
	**Mean**	0.98	1.28	2.12	1.43
	SD	1.36	1.54	1.80	1.63
	Minimum	0.0	0.0	0.0	0.0
	Median	0.00	0.50	2.00	1.00
	Maximum	5.0	5.5	6.0	6.0
7	N_{valid}	122	88	98	308
	$N_{missing}$	0	0	0	0
	**Mean**	0.76	1.11	1.85	1.21
	SD	1.27	1.55	1.78	1.59
	Minimum	0.0	0.0	0.0	0.0
	Median	0.00	0.00	1.50	0.00
	Maximum	5.0	5.0	6.0	6.0

07 - Cough (daily mean) (imputed)		Stratum <= 12 hours (N=122)	Stratum 12 - 24 hours (N=88)	Stratum > 24 hours (N=98)	Total (N=308)
8	N_{valid}	122	88	98	308
	$N_{missing}$	0	0	0	0
	**Mean**	0.72	0.98	1.51	1.05
	SD	1.34	1.47	1.63	1.51
	Minimum	0.0	0.0	0.0	0.0
	Median	0.00	0.00	1.00	0.00
	Maximum	5.5	5.0	6.0	6.0
9	N_{valid}	122	88	98	308
	$N_{missing}$	0	0	0	0
	**Mean**	0.55	0.82	1.14	0.81
	SD	1.12	1.41	1.51	1.35
	Minimum	0.0	0.0	0.0	0.0
	Median	0.00	0.00	0.50	0.00
	Maximum	5.0	5.0	5.5	5.5
10	N_{valid}	122	88	98	308
	$N_{missing}$	0	0	0	0
	**Mean**	0.49	0.69	0.92	0.68
	SD	1.05	1.31	1.42	1.26
	Minimum	0.0	0.0	0.0	0.0
	Median	0.00	0.00	0.00	0.00
	Maximum	4.5	5.0	5.5	5.5
11	N_{valid}	122	88	98	308
	$N_{missing}$	0	0	0	0
	**Mean**	0.39	0.58	0.76	0.56
	SD	0.91	1.22	1.41	1.18
	Minimum	0.0	0.0	0.0	0.0
	Median	0.00	0.00	0.00	0.00
	Maximum	5.0	5.0	5.5	5.5
12	N_{valid}	122	88	98	308
	$N_{missing}$	0	0	0	0
	**Mean**	0.25	0.47	0.61	0.43
	SD	0.75	1.15	1.26	1.06
	Minimum	0.0	0.0	0.0	0.0
	Median	0.00	0.00	0.00	0.00
	Maximum	5.0	5.0	5.0	5.0
13	N_{valid}	122	88	98	308
	$N_{missing}$	0	0	0	0
	**Mean**	0.24	0.40	0.54	0.38
	SD	0.79	1.06	1.21	1.02
	Minimum	0.0	0.0	0.0	0.0
	Median	0.00	0.00	0.00	0.00
	Maximum	5.0	5.0	5.0	5.0
14	N_{valid}	122	88	98	308
	$N_{missing}$	0	0	0	0
	**Mean**	0.21	0.31	0.43	0.31
	SD	0.78	0.98	1.13	0.96
	Minimum	0.0	0.0	0.0	0.0
	Median	0.00	0.00	0.00	0.00
	Maximum	5.0	5.0	6.0	6.0

07 - Cough (daily mean) (imputed)		Stratum <= 12 hours (N=122)	Stratum 12 - 24 hours (N=88)	Stratum > 24 hours (N=98)	Total (N=308)
**15**	N_{valid}	122	88	98	308
	$N_{missing}$	0	0	0	0
	**Mean**	0.18	0.22	0.25	0.21
	SD	0.67	0.79	0.85	0.76
	Minimum	0.0	0.0	0.0	0.0
	Median	0.00	0.00	0.00	0.00
	Maximum	4.0	4.5	5.0	5.0
**16**	N_{valid}	122	88	98	308
	$N_{missing}$	0	0	0	0
	**Mean**	0.18	0.20	0.21	0.19
	SD	0.66	0.76	0.82	0.74
	Minimum	0.0	0.0	0.0	0.0
	Median	0.00	0.00	0.00	0.00
	Maximum	4.0	4.5	5.0	5.0
**17**	N_{valid}	122	88	98	308
	$N_{missing}$	0	0	0	0
	**Mean**	0.18	0.19	0.19	0.19
	SD	0.66	0.75	0.77	0.72
	Minimum	0.0	0.0	0.0	0.0
	Median	0.00	0.00	0.00	0.00
	Maximum	4.0	4.5	4.0	4.5
