# Supplementary material for: The impact of cineole treatment timing on common cold duration and symptoms: Non-randomized exploratory clinical trial
Source: PLoS One. 2024 Jan 18;19(1):e0296482. doi: 10.1371/journal.pone.0296482 (PMC10795983; doi:10.1371/journal.pone.0296482)
Supplement: S9 Table — (PDF) [file pone.0296482.s009.pdf]

S9 Table: Symptom severity peak

Symptom severity peak	Time to treatment stratum ≤12 h (N=122)	>12 to 24 h (N=88)	>24 h (N=98)	Total (N=308)
N_{valid}	122	88	98	308
$N_{missing}$	0	0	0	0
Mean	29.9	33.0	35.1	32.4
SD	10.7	9.6	10.4	10.5
Minimum	4	9	11	4
Median	30.0	34.0	33.3	32.5
Maximum	57	54	56	57
