# Supplementary material for: The impact of cineole treatment timing on common cold duration and symptoms: Non-randomized exploratory clinical trial
Source: PLoS One. 2024 Jan 18;19(1):e0296482. doi: 10.1371/journal.pone.0296482 (PMC10795983; doi:10.1371/journal.pone.0296482)
Supplement: S10 Table — (PDF) [file pone.0296482.s010.pdf]

S10 Table: Time to symptom relief

	Time to treatment stratum			Total
**Time to symptom relief (Days)**	**≤12 h (N=122)**	**>12 to 24 h (N=88)**	**>24 h (N=98)**	**(N=308)**
N_{valid}	121	87	94	302
$N_{missing}$	1	1	4	6
Mean	3.9	4.7	5.2	4.5
SD	2.3	2.8	2.9	2.7
Minimum	1	1	1	1
Median	3.0	4.0	4.0	4.0
Maximum	14	14	14	14
