# Supplementary material for: The impact of cineole treatment timing on common cold duration and symptoms: Non-randomized exploratory clinical trial
Source: PLoS One. 2024 Jan 18;19(1):e0296482. doi: 10.1371/journal.pone.0296482 (PMC10795983; doi:10.1371/journal.pone.0296482)
Supplement: S11 Table — (PDF) [file pone.0296482.s011.pdf]

S11 Table: Investigator assessment of common cold symptoms

| Time to total JSS (Days) | Time to treatment stratum ≤12 h (N=122) | >12 to 24 h (N=88) | >24 h (N=98) | Total (N=308) |
|---|---|---|---|---|
| **Visit 2** | | | | |
| $N_{valid}$ | 122 | 88 | 98 | 308 |
| $N_{missing}$ | 0 | 0 | 0 | 0 |
| Mean | 10.2 | 10.4 | 11.2 | 10.6 |
| SD | 3.2 | 3.2 | 3.5 | 3.3 |
| Minimum | 4 | 4 | 5 | 4 |
| Median | 10.0 | 10.0 | 10.5 | 10.0 |
| Maximum | 21 | 19 | 22 | 22 |
| **Visit 3** | | | | |
| $N_{valid}$ | 119 | 88 | 97 | 304 |
| $N_{missing}$ | 3 | 0 | 1 | 4 |
| Mean | 7.1 | 7.4 | 8.8 | 7.7 |
| SD | 4.0 | 3.4 | 3.6 | 3.8 |
| Minimum | 0 | 2 | 1 | 0 |
| Median | 7.0 | 7.0 | 9.0 | 7.5 |
| Maximum | 20 | 16 | 20 | 20 |
| **Visit 4** | | | | |
| $N_{valid}$ | 122 | 88 | 98 | 308 |
| $N_{missing}$ | 0 | 0 | 0 | 0 |
| Mean | 0.3 | 0.3 | 0.3 | 0.3 |
| SD | 1.6 | 1.0 | 1.3 | 1.4 |
| Minimum | 0 | 0 | 0 | 0 |
| Median | 0.0 | 0.0 | 0.0 | 0.0 |
| Maximum | 15 | 5 | 9 | 15 |

JSS: Jackson Symptom Score
