# Supplementary material for: The impact of cineole treatment timing on common cold duration and symptoms: Non-randomized exploratory clinical trial
Source: PLoS One. 2024 Jan 18;19(1):e0296482. doi: 10.1371/journal.pone.0296482 (PMC10795983; doi:10.1371/journal.pone.0296482)
Supplement: S12 Table — (PDF) [file pone.0296482.s012.pdf]

S12 Table: Days of bed rest due to common cold

| Days of bed rest due to common cold | Time to treatment stratum ≤12 h (N=122) | >12 to 24 h (N=88) | >24 h (N=98) | Total (N=308) |
|---|---|---|---|---|
| $N_{valid}$ | 122 | 88 | 98 | 308 |
| $N_{missing}$ | 0 | 0 | 0 | 0 |
| Mean | 0.6 | 0.6 | 0.6 | 0.6 |
| SD | 1.5 | 1.4 | 1.2 | 1.4 |
| Minimum | 0 | 0 | 0 | 0 |
| Median | 0.0 | 0.0 | 0.0 | 0.0 |
| Maximum | 12 | 8 | 5 | 12 |
