# Supplementary material for: The impact of cineole treatment timing on common cold duration and symptoms: Non-randomized exploratory clinical trial
Source: PLoS One. 2024 Jan 18;19(1):e0296482. doi: 10.1371/journal.pone.0296482 (PMC10795983; doi:10.1371/journal.pone.0296482)
Supplement: S13 Table — (PDF) [file pone.0296482.s013.pdf]

S13 Table: Summary of reported adverse events

Category	AEs with suspected causal relationship to IMP (N=329)		All AEs (N=329)	
Subcategory	**AEs[1]**	**n patients (%)**	**AEs[2]**	**n patients (%)**
**Overall**			**25**	**22 (6.7)**
Related	17	14 (4.3)	17	14 (4.3)
Not related			8	8 (2.4)
**Intensity**				
Mild	11	10 (3.0)	17	16 (4.9)
Moderate	6	4 (1.2)	7	5 (1.5)
Severe	0	0 (0.0)	1	1 (0.3)
**Action taken**				
IMP treatment not changed	12	10 (3.0)	13	11 (3.3)
Concomitant medication given	0	0 (0.0)	6	6 (1.8)
Discontinued prematurely	1	1 (0.3)	3	3 (0.9)
Reduced IMP dose	3	3 (0.9)	3	3 (0.9)
Interrupted IMP	1	1 (0.3)	1	1 (0.3)
**Outcome**				
Resolved	17	14 (4.3)	23	20 (6.1)
Ongoing	0	0 (0.0)	2	2 (0.6)
**SAE**	0	0 (0.0)	0	0 (0.0)

[1]One subject interrupted treatment for 2 days due to diarrhoea (moderate), one subject discontinued prematurely due to vomiting (moderate). [2]Early discontinuation of two subjects due to Tonsilitis bacterial (severe) and diverticulitis (moderate). AE: adverse event; IMP: investigational medicinal product; SAE: serious adverse event.
