# Supplementary material for: The impact of cineole treatment timing on common cold duration and symptoms: Non-randomized exploratory clinical trial
Source: PLoS One. 2024 Jan 18;19(1):e0296482. doi: 10.1371/journal.pone.0296482 (PMC10795983; doi:10.1371/journal.pone.0296482)
Supplement: S1 File — (DOCX) [file pone.0296482.s014.docx]

**Source data for results presented within the manuscript (in addition to supplementary tables)**

GLM Model 1 (basic) for AUC-WURSS (mITT, N=308)

|  | | **AUC-WURSS (imputed)** | | | **Type 3  two-sided  p-values** |
| --- | --- | --- | --- | --- | --- |
| **Statistic** | | **Time to treatment stratum** | | |  |
|  |  | **≤12 h** | **>12 to 24 h** | **>24 h** |  |
| LS mean  [95% CI]^1^ | | 134.0  [112.9, 155.1] | 171.7  [147.8, 195.5] | 229.5  [205.6, 253.4] |  |
| LS means difference  [95% CI]^2^ | | -95.5  [-123.5, -67.5] | -57.8  [-87.8, -27.8] | Not applicable |  |
| Type 3 effects (fixed factors)^1^: | | | | | |
|  | Time to treatment stratum | |  |  | <.0001 |
|  | Age |  |  |  | 0.7349 |
|  | Sex |  |  |  | 0.4599 |
|  | Previous influenza vaccination | |  |  | 0.0011 |
|  | Baseline WURSS-11 score | |  |  | <.0001 |

^1^ Model contains time-to-treatment strata, baseline total symptom score, sex, age, vaccination status. Non-overlapping CIs of LS-Means between two strata indicate a significant difference between the strata. ^2^ Compared with initiation of therapy at > 24 hours after start of illness. If 0 was outside the CI for the LS-Means difference, this comparison was significant.

GLM Model 2 for AUC-WURSS (mITT, N=308; source for Fig. 3)

|  | | **AUC-WURSS (imputed)** | | | **Type 3  two-sided  p-values** |
| --- | --- | --- | --- | --- | --- |
| **Statistic** | | **Time to treatment stratum** | | |  |
|  |  | **≤12 h** | **>12 to 24 h** | **>24 h** |  |
| LS mean  [95% CI]^1^ | | 143.1  [117.7, 168.5] | 181.6  [152.5, 210.8] | 232.0  [203.4, 260.5] |  |
| LS means difference  [95% CI]^2^ | | -88.9  [-118.0, -59.8] | -50.3  [-82.0, -18.7] | Not applicable |  |
| Type 3 effects (fixed factors)^1^: | | | | | |
|  | Time to treatment stratum | |  |  | <.0001 |
|  | Previous influenza vaccination | |  |  | 0.0028 |
|  | Alcohol consumption (categorized) | |  |  | 0.0002 |
|  | Working status | |  |  | 0.0164 |
|  | Baseline WURSS-11 score (categorized) | | |  | <.0001 |

^1^ Model contains time-to-treatment stratum, previous influenza vaccination, alcohol consumption, working status and categorized baseline WURSS-11 score. Non-overlapping CIs of LS-Means between two strata indicate a significant difference between the strata. ^2^ Compared with initiation of therapy at > 24 hours after start of illness. If 0 was outside the CI for the LS-Means difference, this comparison was significant.

Mean daily dose (SAF, N=329)

|  | **Time to treatment stratum** | | | **Total** |
| --- | --- | --- | --- | --- |
| **Mean daily dose (mg/day)** | **≤12 h**  **(N=129)** | **>12 to 24 h**  **(N=94)** | **>24 h**  **(N=105)** | **(N=329) ^1^** |
| N_valid_ | 129 | 94 | 105 | 329 |
| N_missing_ | 0 | 0 | 0 | 0 |
| Mean | 563.60 | 572.48 | 572.96 | 568.87 |
| SD | 55.64 | 44.08 | 75.66 | 60.15 |
| Minimum | 311.1 | 300.0 | 100.0 | 100.0 |
| Median | 575.00 | 580.00 | 583.33 | 580.00 |
| Maximum | 771.4 | 672.7 | 920.0 | 920.0 |

^1^ One subject (24-009) could not be assigned to any stratum, this subject had a mean daily dose of 480 mg/day.

Duration of IMP intake (SAF, N=329)

|  | **Time to treatment stratum** | | | **Total** |
| --- | --- | --- | --- | --- |
| **Duration of IMP intake (days)** | **≤12 h**  **(N=129)** | **>12 to 24 h**  **(N=94)** | **>24 h**  **(N=105)** | **(N=329)**^1^ |
|  | **N** | **N** | **N** | **N** |
| N_valid_ | 129 | 94 | 105 | 329 |
| N_missing_ | 0 | 0 | 0 | 0 |
| Mean | 9.6 | 9.7 | 9.7 | 9.6 |
| SD | 3.4 | 3.4 | 3.5 | 3.4 |
| Minimum | 2 | 2 | 1 | 1 |
| Median | 9.0 | 9.0 | 9.0 | 9.0 |
| Maximum | 17 | 17 | 17 | 17 |

^1^ One subject (24-009) could not be assigned to any stratum, this subject had a duration of IMP intake of 5.0 days.

Percentage of patients with rescue medication – Modified Intention-to-Treat Set

**
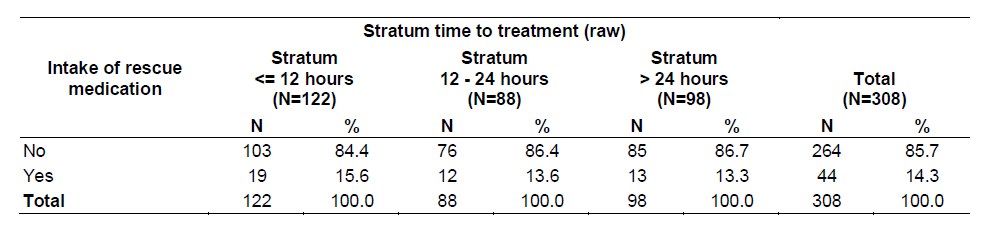
**

AUC WURSS - Modified Intention-to-Treat Set


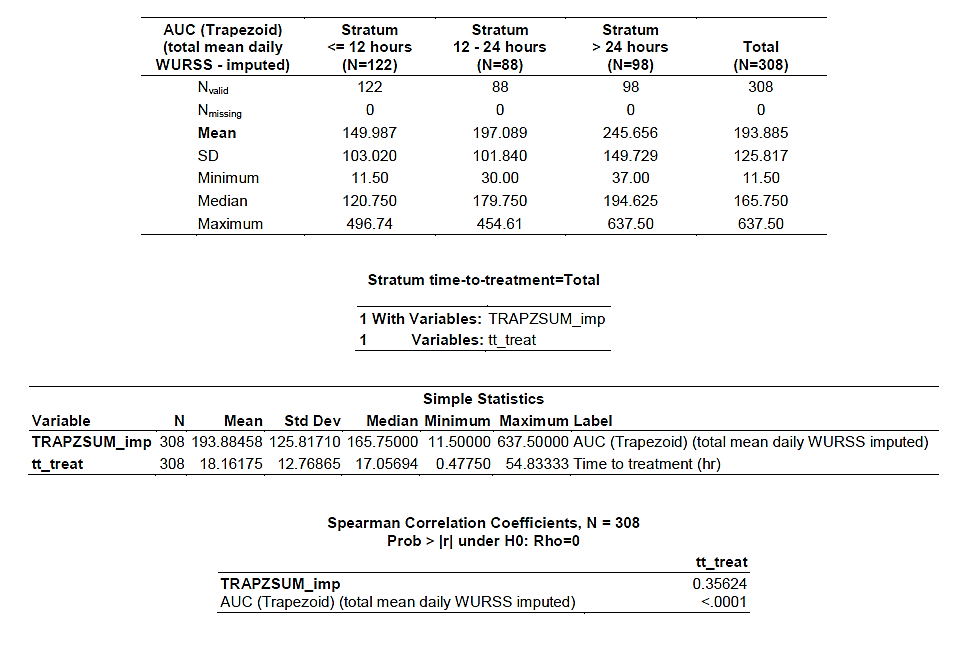


LS-Means [95% CI] of WURSS-11 mean daily total score for each time to treatment stratum and [95% CI] for LS-Means difference between stratum ≤12 h and >24 h using MMRM Model 2 data (mITT, N=308; source for Fig. 4)

| **Symptom Day** | **LS-Means**  **[95% CI] for strata:** | | | **[95% CI] of  LS-Means difference between ≤12h and >24h** |  |
| --- | --- | --- | --- | --- | --- |
|  | **≤12 h** | **>12 to 24 h** | **>24 h** |  |  |
| 1 | 27.61 [25.73, 29.49] | 28.87 [26.67, 31.07] | 28.65 [26.55, 30.76] | [-3.71, 1.63] |  |
| 2 | 24.80 [22.88, 26.72] | 28.56 [26.31, 30.81] | 30.46 [28.31, 32.60] | [-8.39, -2.93] | * |
| 3 | 20.41 [18.36, 22.46] | 26.30 [23.90, 28.71] | 29.65 [27.35, 31.94] | [-12.18, -6.30] | * |
| 4 | 16.72 [14.59, 18.85] | 23.11 [20.62, 25.61] | 27.65 [25.27, 30.03] | [-13.99, -7.87] | * |
| 5 | 13.36 [11.21, 15.52] | 19.30 [16.77, 21.82] | 24.76 [22.35, 27.17] | [-14.49, -8.30] | * |
| 6 | 10.41 [8.24, 12.59] | 15.38 [12.83, 17.93] | 20.87 [18.43, 23.30] | [-13.59, -7.32] | * |
| 7 | 7.97 [5.78, 10.15] | 11.76 [9.20, 14.33] | 18.05 [15.61, 20.50] | [-13.24, -6.94] | * |
| 8 | 6.05 [3.93, 8.17] | 9.36 [6.88, 11.84] | 14.31 [11.95, 16.68] | [-11.30, -5.22] | * |
| 9 | 4.36 [2.37, 6.35] | 6.84 [4.51, 9.17] | 11.23 [9.01, 13.46] | [-9.71, -4.03] | * |
| 10 | 3.57 [1.68, 5.45] | 5.31 [3.11, 7.52] | 9.05 [6.95, 11.15] | [-8.15, -2.81] | * |
| 11 | 2.71 [1.03, 4.38] | 3.30 [1.34, 5.25] | 7.36 [5.49, 9.23] | [-6.99, -2.31] | * |
| 12 | 1.56 [0.10, 3.03] | 2.24 [0.53, 3.95] | 5.43 [3.79, 7.07] | [-5.87, -1.87] | * |
| 13 | 1.06 [0.00, 2.42] | 1.60 [0.01, 3.18] | 4.50 [2.97, 6.02] | [-5.27, -1.62] | * |
| 14 | 0.67 [0.00, 1.87] | 0.91 [0.00, 2.30] | 3.01 [1.66, 4.36] | [-3.89, -0.78] | * |
| 15 | 0.31 [0.00, 1.43] | 0.36 [0.00, 1.66] | 2.06 [0.81, 3.32] | [-3.18, -0.34] | * |
| 16 | 0.16 [0.00, 1.20] | 0.24 [0.00, 1.44] | 1.49 [0.32, 2.66] | [-2.60, -0.06] | * |
| 17 | 0.09 [0.00, 1.13] | 0.13 [0.00, 1.33] | 1.38 [0.21, 2.54] | [-2.55, -0.02] | * |

* = Significant LS-Means difference.

Comparison of time to treatment strata using MMRM Model 2 data for WURSS-11 mean daily QoL score (mITT, N=308; source for Fig. 5)

| **Symptom Day** | **LS-Means**  **[95% CI] for strata:** | | | **[95% CI] of  LS-Means difference between ≤12h and >24h** |  |
| --- | --- | --- | --- | --- | --- |
|  | **≤12 h** | **>12 to 24 h** | **>24 h** |  |  |
| 1 | 6.07  [5.49, 6.65] | 6.03  [5.35, 6.72] | 6.23  [5.57, 6.88] | [-1.00, 0.69] |  |
| 2 | 5.78  [5.22, 6.34] | 6.38  [5.73, 7.04] | 6.68  [6.05, 7.30] | [-1.70, -0.09] | * |
| 3 | 4.76  [4.16, 5.36] | 5.99  [5.29, 6.69] | 6.62  [5.95, 7.29] | [-2.73, -1.00] | * |
| 4 | 3.87  [3.27, 4.47] | 5.21  [4.50, 5.91] | 6.18  [5.51, 6.86] | [-3.19, -1.44] | * |
| 5 | 3.13  [2.54, 3.72] | 4.38  [3.69, 5.07] | 5.49  [4.84, 6.15] | [-3.22, -1.52] | * |
| 6 | 2.43  [1.87, 3.00] | 3.53  [2.86, 4.20] | 4.64  [4.01, 5.28] | [-3.03, -1.39] | * |
| 7 | 1.96  [1.39, 2.53] | 2.61  [1.94, 3.28] | 4.11  [3.47, 4.74] | [-2.97, -1.33] | * |
| 8 | 1.52  [0.98, 2.07] | 2.09  [1.45, 2.74] | 3.27  [2.66, 3.89] | [-2.54, -0.96] | * |
| 9 | 1.07  [0.55, 1.58] | 1.50  [0.89, 2.10] | 2.67  [2.09, 3.24] | [-2.34, -0.87] | * |
| 10 | 0.91  [0.42, 1.40] | 1.17  [0.59, 1.74] | 2.25  [1.70, 2.80] | [-2.04, -0.64] | * |
| 11 | 0.69  [0.26, 1.11] | 0.73  [0.23, 1.22] | 1.79  [1.31, 2.27] | [-1.70, -0.51] | * |
| 12 | 0.38  [0.00, 0.75] | 0.46  [0.03, 0.90] | 1.42  [1.00, 1.84] | [-1.55, -0.53] | * |
| 13 | 0.26  [0.00, 0.59] | 0.32  [0.00, 0.71] | 1.16  [0.79, 1.54] | [-1.35, -0.46] | * |
| 14 | 0.18  [0.00, 0.48] | 0.20  [0.00, 0.55] | 0.80  [0.46, 1.13] | [-0.99, -0.24] | * |
| 15 | 0.08  [0.00, 0.37] | 0.11  [0.00, 0.45] | 0.57  [0.24, 0.89] | [-0.84, -0.12] | * |
| 16 | 0.06  [0.00, 0.33] | 0.09  [0.00, 0.41] | 0.42  [0.12, 0.73] | [-0.70, -0.04] | * |
| 17 | 0.04  [0.00, 0.30] | 0.08  [0.00, 0.38] | 0.35  [0.06, 0.64] | [-0.62, -0.01] | * |

* = Significant LS-Means difference.

AFT Model 2 for time to remission (mITT, N=308; source for Fig. 6)

|  | **Time to treatment stratum** | | |
| --- | --- | --- | --- |
| **AFT Statistics** | **≤12 h** | **>12 to 24 h** | **>24 h** |
| Geometric mean  [95% CI] | 8.9  [8.2, 9.7] | 10.3  [9.4, 11.4] | 10.7  [9.7, 11.8] |
| Acceleration factor  [95% CI] | 0.833  [0.754, 0.920] | 0.964  [0.865, 1.073] | Not applicable |

Note: The geometric mean 95% CIs between strata ≤12 h and >24 h did not overlap: the 95% CI for stratum ≤12 h was [8.1937; 9.7307] and for stratum >24 h it was [09.7311; 11.8088]. As such, a significant difference between strata (p < 0.05) could be concluded.

WURSS-11 time to symptom relief: Descriptive statistics – Modified Intention-to-Treat Set

**
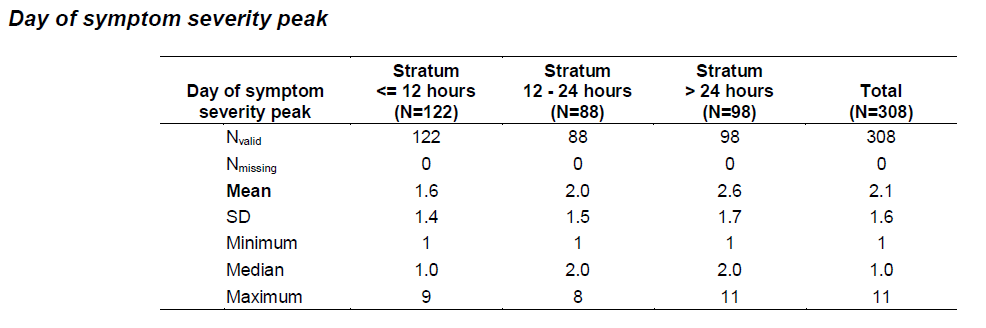
**

Days of sick leave – Modified Intention-to-Treat Set

**
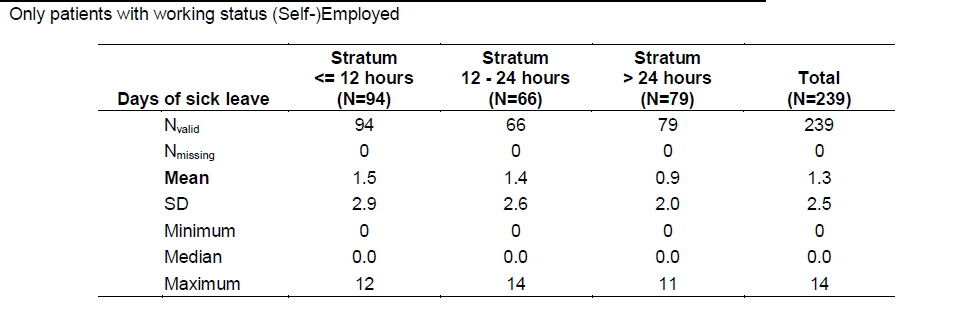
**

**Global judgement of efficacy by investigator – Modified Intention-to-Treat Set**

**
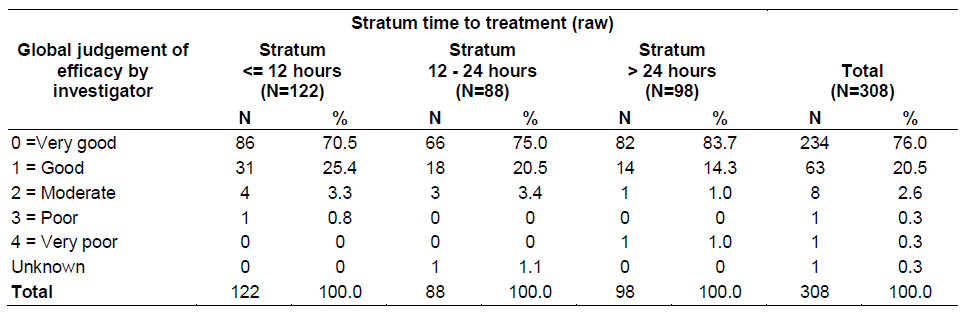
**Global judgement of tolerability by the investigator and the subject (SAF, N=329)

|  | **Total** |  |
| --- | --- | --- |
| **Global judgement of tolerability** | **(N=329)** |  |
|  | **n** | **%** |
| **By the investigator** |  |  |
| 0 = Very good | 266 | 80.9 |
| 1 = Good | 52 | 15.8 |
| 2 = Moderate | 6 | 1.8 |
| 3 = Poor | 0 | 0.0 |
| 4 = Very poor | 1 | 0.3 |
| Not done | 3 | 0.9 |
| Unknown | 1 | 0.3 |
| **By the subject** |  |  |
| 0 = Very good | 224 | 68.1 |
| 1 = Good | 90 | 27.4 |
| 2 = Moderate | 6 | 1.8 |
| 3 = Poor | 4 | 1.2 |
| 4 = Very poor | 1 | 0.3 |
| Not done | 3 | 0.9 |
| Unknown | 1 | 0.3 |
