# Supplementary material for: The impact of cineole treatment timing on common cold duration and symptoms: Non-randomized exploratory clinical trial
Source: PLoS One. 2024 Jan 18;19(1):e0296482. doi: 10.1371/journal.pone.0296482 (PMC10795983; doi:10.1371/journal.pone.0296482)
Supplement: S2 File — (PDF) [file pone.0296482.s015.pdf]

EudraCT No.: 2020-000860-51

CLINICAL TRIAL PROTOCOL

The effect of early administered cineole on the course of a common cold

An open-label, non-randomized, multicenter Phase IV trial

EudraCT number:	2020-000860-51
Protocol version no.:	Final 3
Protocol date:	18 November 2020
Sponsor’s clinical trial protocol code:	CASK0120
Pharmalog project number:	19542
Investigational medicinal product (IMP):	Soledum® forte capsules (cineole 200 mg)
Co-ordinating investigator:<br>„Leiter der klinischen Prüfung“ according to ICH-GCP and §40 German drug law	Prof. Dr. med. Andreas Michalsen<br>Immanuel Krankenhaus Berlin<br>Königstraße 63<br>14109 Berlin-Wannsee (Germany)
Clinical research organization (CRO):	PHARMALOG Institut für klinische Forschung GmbH<br>Oskar-Messter-Str. 29<br>85737 Ismaning (Germany)
Statistical planning:	Holger Stammer, Monika Rost<br>PHARMALOG Institut für klinische Forschung GmbH
Authors of clinical trial protocol:	Dr. med. Norbert Gmeinwieser<br>Monika Rost<br>PHARMALOG Institut für klinische Forschung GmbH
Sponsor:	Cassella-med GmbH & Co. KG<br>Gereonsmühlengasse 1<br>50670 Köln (Germany)
Scientific project manager at sponsor:	Dr. Kim Goldenstein<br>(address see “Sponsor”)

TABLE OF CONTENTS

Page

1 LIST OF ABBREVIATIONS AND TERMS 7

2 SYNOPSIS 10

3 INTRODUCTION 23

3.1 Medical background 23

3.2 Drug characteristics 23

3.2.1 Description of clinical trial rationale 23

3.3 Risk-benefit assessment 24

3.4 Subsequent treatment and medical attendance after clinical trial termination 24

3.5 References / guidelines 24

4 Clinical TRIAL OBJECTIVES 25

5 CLINICAL TRIAL PLAN 26

5.1 Clinical trial design 26

5.2 Clinical trial centers 26

5.3 Subject selection, sample size 26

5.4 Clinical trial visits 27

5.5 Planned trial duration 27

5.6 End of trial 27

6 SUBJECT POPULATION AND INCLUSION / EXCLUSION CRITERIA 28

6.1 Definition of subjects / inclusion diagnosis 28

6.2 Inclusion criteria 28

6.3 Exclusion criteria 28

6.4 Previous and concomitant medication 30

6.4.1 Previous medication not permitted 30

6.4.2 Concomitant medication not permitted 30

6.4.3 Permitted medication and measures 30

6.5 Declaration regarding inclusion of subjects who are possibly dependent on the sponsor or the investigator 31

7 CLINICAL TRIAL THERAPY 32

7.1 Description of clinical trial medication and control 32

7.1.1 Identity of clinical trial medication 32

7.1.2 Manufacturing and packaging of Soledum® forte capsules 32

7.1.3 Packaging and Labelling 33

7.2 Release of trial medication 33

7.3 Shipment and drug accountability 33

7.4 Storage 33

7.5 Drug supply per subject ... 34
7.6 Dosage and route of administration, duration of treatment ... 34
7.7 Compliance ... 35
7.8 Blinding, unblinding, emergency codes ... 35

8 CONDUCT OF CLINICAL TRIAL AND ASSESSMENTS ... 36
8.1 Planned visits (Visit 1 - Visit 4) ... 36
8.2 Premature withdrawal ... 38
8.3 Flow chart ... 38

9 METHODOLOGY ... 39
9.1 Subject information and informed consent ... 39
9.2 Demographic data ... 39
9.3 Confounding variables / parameters ... 40
9.4 Confirmation of inclusion diagnosis ... 40
9.5 Jackson symptom score ... 41
9.6 Previous and concomitant diseases ... 41
9.7 Previous and concomitant medication ... 42
9.8 Physical examination ... 42
9.9 Body weight and height ... 43
9.10 Vital signs (blood pressure, pulse rate, body temperature, oxygen saturation) ... 43
9.11 SARS-CoV-2 test ... 43
9.12 Urine pregnancy test ... 43
9.12.1 Investigator's global judgement on efficacy ... 44
9.13 Investigator's and subject's global judgement on tolerability ... 44
9.14 Bronchitis Severity Score (BSS) ... 44
9.15 Wisconsin Upper Respiratory Symptom Survey (WURSS-11) ... 45
9.16 Electronic diary (eDiary) / electronic patient reported outcomes (ePRO) ... 46
9.17 Adverse events (AE) ... 47

10 Adverse events (AE) ... 48
10.1 Definitions ... 48
10.1.1 Adverse event (AE) ... 48
10.1.2 Adverse reactions (ARs) ... 48
10.1.3 Serious adverse events (SAEs) ... 48
10.1.4 Unexpected adverse reactions (UARs) ... 49
10.1.5 Suspected unexpected serious adverse reactions (SUSARs) ... 49
10.1.6 Definition and assessment of the intensity ... 49
10.1.7 Classification and coding of the causal relationship ... 49
10.2 Documentation and reporting of AEs ... 50

10.3 Documentation and reporting of SAEs 51
10.3.1 Reporting duties of the investigator 51
10.3.2 Reporting duties of the sponsor 52
10.4 Follow-up of (S)AE 52
10.5 Handling of pregnancies 52

11 STOPPING RULES AND DISCONTINUATION CRITERIA 54
11.1 Replacement of drop-outs 54
11.2 Discontinuation criteria for individual subjects 54
11.3 Premature discontinuation of the clinical trial at a clinical trial site 55
11.4 Premature discontinuation of the entire clinical trial 55

12 RECORDING AND ARCHIVING OF DATA 56
12.1 Data recording in the eCRF 56
12.2 Archiving 57
12.2.1 Medical files (investigator site) 57
12.2.2 Trial Master File (sponsor site) 57
12.3 Quality assurance 57
12.3.1 Monitoring 58
12.3.2 Source data / source data verification (SDV) 58
12.3.3 Audit 59

13 STATISTICAL METHODS 60
13.1 Statistical analysis plan 60
13.2 General considerations 60
13.3 Analysis sets 60
13.4 Definition of strata and comparability 62
13.4.1 Definition of strata 62
13.4.2 Comparability of strata 62
13.5 Primary endpoint (mITT, PPS) 62
13.5.1 Definition of primary endpoint 62
13.5.2 Analysis of primary endpoint – basic approach 63
13.5.3 Confounders adjustment approach 63
13.5.4 Propensity score approach 63
13.6 Secondary endpoints 63
13.6.1 Secondary endpoint definitions 63
13.6.2 Analyses of secondary endpoints – metric scales 65
13.6.3 Analyses of secondary endpoints – time to event 65
13.7 Baseline parameters / confounders 65
13.8 Compliance and exposure 66
13.9 Interim analyses 66
13.10 Handling of missing data 66

13.11 Sample size estimation 67

14 PERFORMANCE ACCORDING TO GCP 68

14.1 Declaration of Helsinki / Ethical Guidelines 68

14.2 Adherence to the protocol 68

14.3 Summary of Product Characteristics 68

14.4 Data protection 68

14.5 Subject informed consent 69

14.5.1 Subject information 69

14.5.2 Subject informed consent 69

14.6 Information of other physicians 69

14.7 Financial or other interests of the investigator 70

15 LEGAL AND ETHICAL ASPECTS 71

15.1 Notification to authorities and ethics committees 71

15.1.1 Commencement of the clinical trial 71

15.1.2 Ending of the clinical trial 71

15.2 Protocol amendment 71

15.3 Inspection 72

15.4 Direct access to source data / documents 72

15.5 Subject insurance 72

15.6 Contracts, finances 72

16 CLINICAL TRIAL RESULTS 73

16.1 Final report 73

16.2 Publication 73

17 TIME SCHEDULE OF the CLINICAL TRIAL 74

18 LITERATURE 75

19 SUPPLEMENT 78

LIST OF TABLES

Table 1: Flow chart..20
Table 2: Identity of the used drug in this clinical trial..32

Rationale for Amendment and Justification

During the course of the study new test methods for SARS-CoV-2 have been established, i.e. an antigen rapid test is now commercially available on the market. In order to allow the sites also to use this antigen rapid test as an alternative to the already implemented PCR-test, this protocol amendment became necessary. The sites are now allowed to use either the PCR test or the antigen rapid test. The advantage of the antigen rapid test is, that the result is available within a couple of minutes and a decision of exclusion of patient from further study participation can be made immediately. Furthermore, the currently limited test capacities for PCR test are not blocked for study purposes. This change has been considered as a substantial change to the protocol.

In parallel some other minor protocol specifications are to be changed too:

- Behavior in case of influenza vaccination during the screening phase (V1 – V2) and patients react with feverish symptoms
- Replacement of screening failure patients is implemented
- The period of documentation of previous medication is now better specified
- Criteria for timepoint of conduction of interim analysis was specified
- mITT: one criterion was better specified in order to avoid misunderstandings.

These changes are considered to be non-substantial.

1 LIST OF ABBREVIATIONS AND TERMS

**AE**	Adverse event(s)
**AFT**	Accelerated failure time
**AMG**	Arzneimittelgesetz (German drug law)
**AR**	Adverse reaction
**ASA**	Acetylsalicylic acid
**ATC**	Anatomical-therapeutic-chemical (classification system for drugs)
**AUC**	Area under curve
**BMI**	Body mass index
**bpm**	Beats per minute (measurement of pulse)
**CA**	Competent authority
**CD-ROM**	Compact disc read-only memory
**CFR**	Code of Federal Regulations
**CPMP**	Committee for Proprietary Medicinal Products
**CRO**	Contract research organization
**DBL**	Data base lock
**DRM**	Data review meeting
**DSGVO**	Datenschutzgrundverordnung = General Data Protection Regulation (GDPR)
**DVD**	Digital versatile disc
**e.g.**	Exempli gratia (for example)
**EC**	Ethics committee
**ECG**	Electrocardiogram
**eCRF**	Electronic case report form
**EDC**	Electronic data capture
**EMA**	European Medicines Agency
**ENT**	Ears nose throat
**EudraCT**	European clinical trials database
**FAS**	Full analysis set
**GCP**	Good clinical practice
**GDPR**	General Data Protection Regulation
**GLM**	General linear models

**i.e.**	Id est (that is)
**ICF**	Informed consent form
**ICH**	International conference on harmonization
**IMP**	Investigational medicinal product
**IPW**	Inverse probability weights
**IRB**	Institutional Review Board
**ISF**	Investigator site file
**IUD**	Intrauterine device
**kg**	Kilogram
**LKP**	Leiter der klinischen Prüfung (coordinating investigator)
**MedDRA**	Medical dictionary for regulatory activities
**mg**	Milligram
**mITT**	Modified Intention-to-treat set
**mmHg**	Millimetre hydrargyrum = mercury (measurement of blood pressure)
**MRMM**	Mixed models for repeated measurements
**NIMP**	Non-investigational medicinal product
**PDF**	Portable document format
**PPS**	Per protocol set
**RT-PCR**	Reverse transcription polymerase chain reaction
**SAE**	Serious adverse event
**SAF**	Safety set
**SAP**	Statistical analysis plan
**SAR**	Serious adverse reaction
**SARS-CoV-2**	Severe acute respiratory syndrome coronavirus 2
**SDV**	Source data verification
**SmPC**	Summary of product characteristics
**SOP**	Standard operating procedure
**SpO2**	Peripheral capillary oxygen saturation
**SUSAR**	Suspected unexpected serious adverse reactions

**t.i.d.**	Tres in diem (three times a day)
**TMF**	Trial master file
**vs.**	Versus
**WHO**	World Health Organization

2 SYNOPSIS

EudraCT number	2020-000860-51
Protocol code	CASK0120
Protocol title	The effect of early administered cineole on the course of a common cold
Clinical trial design	Open-label, non-randomized, exploratory, multicenter clinical trial
Clinical phase	Phase IV
Investigators	General practitioners, otolaryngologists (ENT specialists), specialists in internal medicine
Number of centers	Approximately 25 centers
Number of subjects	Inclusion of approximately 400 – 500 adult subjects in order to get 330 evaluable subjects under treatment with the investigational medicinal product (IMP)
Countries	Germany
Clinical trial objectives	To investigate the relation between timing of treatment onset with cineole and the course of a common cold with or without acute bronchitis
Clinical trial duration	In total: Up to 8 months<br>Per subject: Screening phase: up to 7 months<br>Illness phase: maximum 15 -2/+4 days<br>Treatment phase: maximum 15 ±2 days
Planned trial period	FSFV (first subject, first visit): September / October 2020<br>LSO (last subject out): End of March / April 2021
Clinical trial visits	4 scheduled visits in total:<br><u>Screening phase</u>:<br>Visit 1 (recruitment): Informed consent, screening of subjects, training of eDiary on subject's smartphone / tablet / computer<br><br><u>Illness phase:</u><br>Up to 48 hours prior 1st IMP intake: Symptom onset, i.e. start of eDiary documentation<br>Visit 2 (Day 1): Inclusion diagnosis, start of treatment<br>Visit 3 (Day 4± 1): Control visit<br>Visit 4 (at common cold recovery but latest Day 15±2): Final visit: end of treatment and clinical trial<br><br><u>Treatment phase:</u><br>Visit 2 (Day 1) till common cold recovery but latest till Day 15±2<br><br>Subjects are advised to start the eDiary documentation immediately once they feel that they have a common cold and to contact the site for an appointment (for further details see "Methodology" below). The appointment should take place ideally on the same day when the subject feels he[1] has a common cold.<br>Subjects who do not meet the inclusion diagnosis at Visit 2 (see inclusion criteria no. 7 to 10) are allowed to continue screening phase and will become eligible for a new common cold episode after complete recovery of current episode.

[1] In the framework of this text, the male term always includes the female term.

Follow-up observation	None
Methodology	<u>Visit 1 (up to 7 months before Visit 2): Screening</u><br>▪ Subject information, subject's written informed consent<br>▪ Demographic data: age, gender, ethnic origin<br>*only females*: childbearing potential:<br>if yes: method of contraception<br>if no: date of menopause (at least year) or other measures<br>▪ Body height and weight (questioning the subject); body mass index (BMI) will be automatically calculated in the eCRF<br>▪ Confounding variables / parameters:<br>o Number of common colds during previous winter season<br>o Alcohol consumption<br>o Smoking habits<br>o Working status<br>▪ Previous and concomitant diseases<br>▪ Concomitant medication<br>▪ Physical examination<br>▪ *Females of childbearing potential only*: urine pregnancy test<br>▪ Check of inclusion and exclusion criteria applicable at Visit 1<br>▪ Training of eDiary<br><br><u>Visit 2 (Day 1): inclusion diagnosis, start of treatment:</u><br>▪ Current number of common cold episodes within this clinical trial (technical parameter)<br>▪ Check of eDiary entries<br>▪ Days of sick-leave (for subjects working employed or self-employed) & days of bed rest due to common cold since day of onset<br>▪ Confounding variable / parameter:<br>o Vaccination against influenza for the coming / current winter season<br>▪ Jackson Symptom Score (JSS)<br>▪ Inclusion diagnosis: common cold (for details see "Methodology" and inclusion criteria 7 to 10)<br>▪ Common cold complications, e.g. otitis media, pneumonia, tonsillitis<br>▪ Pharyngeal swab for SARS-CoV-2 test via RT-PCR <u>or</u> antigen rapid test<br>▪ *Only if cough is present*: Bronchitis Severity Score (BSS)<br>▪ Previous and concomitant diseases (update)<br>▪ Previous and concomitant medication (update)<br>▪ Physical examination<br>▪ Vital signs: systolic and diastolic blood pressure, pulse, body temperature, oxygen saturation [SpO_2] of the blood<br>▪ *Females of childbearing potential only:* urine pregnancy test<br>▪ Check of inclusion and exclusion criteria applicable at Visit 2<br>▪ Dispense of clinical trial medication (IMP)<br>▪ First administration of IMP in the investigator's office and documentation in eDiary<br>▪ *Subjects with fever and / or headache only*: Dispense of rescue medication: paracetamol<br><u>Note</u>: Visit 2 must not take place on the same day as Visit 1. For details refer to subsection "Subject selection, screening phase" in this synopsis.

<table>
<tr><td>Methodology (continued)</td><td>
<u>Visit 3 (Day 4±1): control visit:</u>

Check of eDiary entries
Jackson Symptom Score (JSS)
Common cold complications, e.g. otitis media, pneumonia, tonsillitis
Only if cough is present at this or a previous visit: Bronchitis Severity Score (BSS)
Concomitant medication (changes)
Physical examination
Vital signs: systolic and diastolic blood pressure, pulse, body temperature
Check of withdrawal criteria
Check / documentation of adverse events
Days of sick-leave (for subjects working employed or self-employed) & days of bed rest due to common cold since previous visit
Drug accountability (pill count: IMP & if applicable rescue medication)
Only subjects with new onset of fever and / or headache having not received rescue medication at Visit 2: Dispense of rescue medication: paracetamol

<u>Visit 4 (at common cold recovery but latest Day 15±2): final visit: end of treatment and clinical trial:</u>

Check of eDiary entries
Jackson Symptom Score (JSS)
Common cold complications, e.g. otitis media, pneumonia, tonsillitis
Only if cough is present at this or a previous visit: Bronchitis Severity Score (BSS)
Concomitant medication (changes)
Physical examination
Vital signs: systolic and diastolic blood pressure, pulse, body temperature
Females of childbearing potential only: urine pregnancy test
Check / documentation of adverse events
Investigator’s global judgement on efficacy
Investigator’s and subject’s global judgment on tolerability
Days of sick-leave (for subjects working employed or self-employed) & days of bed rest due to common cold since previous visit
Recording of clinical trial termination
Drug accountability (pill count and collection IMP & if applicable rescue medication)

Visit 4 may be performed earlier and if applicable also instead of Visit 3, when the subject has recovered from common cold before Day 15±2. A subject is recovered, in case he rated the items 1-10 of the WURSS-11 in the eDiary with “0” (for details see below) on two consecutive assessments after Visit 2.
Assessments of Visit 4 will be performed in case of subject’s premature termination of the clinical trial but only in subjects who had at least one administration of IMP. If subject is withdrawn from the clinical trial, Visit 4 shall be performed as soon as possible.
</td></tr>
</table>

Methodology (continued)	Subject selection, screening phase<br>Subjects visiting their general practitioner, ENT specialist or specialist in internal medicine for any reason will be offered to participate in this clinical trial (i.e. Visit 1). Other methods of recruiting may include letters to family doctors, flyers, advertisements. Subjects who have given their informed consent will be screened for eligibility. Subjects fulfilling all inclusion and none of the exclusion criteria applicable at Visit 1 will enter the screening phase:<br>They will be instructed on the use of the eDiary and the need to contact the investigator immediately once he has the impression of having a common cold. Once a week they will get a reminder on their smartphone / tablet / computer and will be asked to start documentation in the eDiary via their smartphone / tablet / computer immediately once they feel having a common cold, which is to be documented in the eDiary and set as onset of common cold. At this time point and subsequently of this twice daily (i.e. in the morning and in the evening) subjects will be asked via reminder to answer all questions of WURSS-11 in the eDiary. In addition, they should make an appointment (i.e. Visit 2) and visit their investigator as soon as possible. Visit 2 needs to be scheduled earlier than 48 hours after start of eDiary documentation. If this is not possible, the subject is not eligible and cannot continue for the current illness episode (including eDiary documentation). However, such a subject may continue screening phase for another illness episode.<br>Note: A first reminder regarding start of eDiary documentation will be sent one week after Visit 1. Subjects consulting their investigator for any kind of acute respiratory tract infection at Visit 1 will receive the first reminder three weeks after Visit 1, to ensure that the current respiratory tract infection is resolved. In addition, the subjects will be instructed not to start eDiary documentation if the respiratory tract infection has not completely disappeared within three weeks after Visit 1.
Methodology (continued)	eDiary / ePRO (electronic patient reported outcomes)<br>A web-based system for electronic collection of subject data (eDiary) will be used in this clinical trial. Subjects participating in this trial must be capable of using the system on their own device (e.g. smartphone, tablet or computer) and be comfortable with it after a short period of training provided by the investigator / site staff at Visit 1.
Inclusion criteria	Visit 1 – Screening<br>1. Age between 18 and 70 years<br>2. Recollecting having ≥1 common cold in the last winter season by asking the subject<br>3. Informed consent, personally dated and signed by the subject and the investigator, and data protection declaration<br>4. Willingness and ability to use an eDiary<br>5. Availability of an appropriate device for eDiary data entry, i.e. smartphone, tablet or computer<br>6. Subject able to follow the instructions given by the investigator<br><br>Visit 2 – Inclusion diagnosis, start of treatment<br>7. Start of eDiary documentation ≤48 hours before first IMP intake<br>8. Common cold with or without bronchitis clinically diagnosed by the investigator<br>9. “Yes” to the question “Does it feel to you that you are developing a cold or that you have a cold?”<br>10. Using Jackson’s criteria, subjects have to report at least a sum score of ≥ 3 in up to the four cold symptoms:<br>▪ Nasal discharge<br>▪ Nasal obstruction<br>▪ Sneezing<br>▪ Sore throat

Exclusion criteria	Visit 1 – Screening<br>1. Any known chronic ear, nose, throat and respiratory tract disease or any other known serious disease that can influence the course of the common cold, e.g. chronic bronchitis (WHO Definition), chronic obstructive pulmonary disease (COPD), bronchial asthma, chronic active allergic rhinitis (e.g. dust mite allergy), chronic rhinosinusitis with or without nasal polyp(s), cystic fibrosis within the past 2 years before Visit 1<br>2. Pneumonia, pertussis and / or pseudo-croup<br>3. Long-term treatment (>14 days) with systemically administered antibiotics within the last 7 days before Visit 1<br>4. Previous SARS-CoV-2 infection confirmed by RT-PCR (based on medical history)<br>5. Known hypersensitivity to cineole or any of the other compounds of Soledum® forte capsules<br>6. Known hereditary fructose intolerance<br>7. Presence of clinically relevant renal and / or liver diseases<br>8. Any respiratory disease or symptoms related to smoking: asymptomatic smokers are allowed to be included<br>9. Presence of immunosuppressive state, malignancy (actual, condition after carcinoma less than 2 years without relapse), autoimmune disease(s), AIDS<br>10. Pregnancy (positive urine pregnancy test) or lactation<br>11. Female of childbearing potential without medically accepted contraception<br>12. History or presence of dependency from alcohol or drugs<br>13. Participation in another clinical study, already terminated or still ongoing, within the last 30 days<br>14. Known to be, or suspected of being unable to comply with the trial protocol (e.g., no permanent address, history of drug abuse, known to be non-compliant or presenting an unstable psychiatric history)<br>15. Legal incapacity and / or other circumstances rendering the subject unable to understand the nature, scope and possible impact of the trial<br>16. Subject in custody by juridical or official order<br>17. Subject who has difficulties in understanding the language (German) in which the subject information (informed consent form) is given<br>18. Subjects who are members of the staff of the trial center, staff of the sponsor or the clinical research organization (CRO), the investigator himself or close relatives of the investigator<br><br>Visit 2 – Inclusion diagnosis, start of treatment<br>Same exclusion criteria as for Visit 1 plus the following ones:<br>19. Start of eDiary documentation longer than 12 h after symptom onset<br>20. Any known acute ear, nose, throat and respiratory tract disease that can influence the course of the common cold e.g. assured diagnosis of the flu, Covid-19 (acute or previous infection since Visit 1), allergic rhinitis (e.g. allergy to a current available seasonal allergen)<br>21. Hypoxemia, i.e. SpO_2 ≤92%<br>22. Any complications of the common cold that have to be treated with antibiotics<br>23. Subjects treated with or need for treatment with antibiotics (systemic), glucocorticosteroids (systemic, per inhalation or nasal), β2-mimetics, theophylline, common cold or cough medication (systemic, per inhalation or nasal) within 7 days prior to Visit 2 or at the time of Visit 2<br>24. Regular treatment (i.e. >2 single doses since onset of common cold) with systemic non-steroidal anti-inflammatory drugs [NSAIDs] / analgesics (except low dose acetylsalicylic acid [ASA]) within 7 days prior to Visit 2 or at the time of Visit 2<br>25. Drugs without marketing authorization or drugs without marketing authorization in the indication they are used for within 7 days prior to Visit 2 or at the time of Visit 2

Exclusion criteria (continued)	26. Angiotensin converting enzyme (ACE) blockers, unless given as long-term therapy over at least 8 weeks on a stable dose prior to enrolment into the study and not causing cough as undesirable effect
Not permitted previous medication	Within 7 days prior to Visit 2<br>▪ Antibiotics (systemic)<br>▪ Glucocorticosteroids (systemic, per inhalation or nasal)<br>▪ β2-mimetics<br>▪ Theophylline<br>▪ Common cold or cough medication, e.g. expectorants (e.g. acetylcysteine) / mucolytics, drops / liquids for nasal decongestion, antitussives, cineole, or other essential oils (systemic or per inhalation), phytotherapeutics (including pharmacy-only lozenges)<br>▪ Treatment with systemic NSAIDs / analgesics (except low dose ASA)<br>▪ Drugs without marketing authorization or drugs without marketing authorization in the indication they are used for<br>Angiotensin converting enzyme (ACE) blockers are also not permitted, unless given as long-term therapy over at least 8 weeks on a stable dose prior to enrolment into the study and not causing cough as undesirable effect
Not permitted concomitant medication	Any not permitted previous medication listed above (cineole as IMP and paracetamol as rescue medication excluded) is also not allowed in the period from Visit 2 to Visit 4.
Permitted concomitant medication / measures	Other continuing pharmacotherapy not listed above in constant dose for diseases other than common cold is allowed.<br>Pure water or salt water steam inhalation without additives are also allowed.<br>Note: Further measures (tea, gargling etc.) with non-pharmaceutical products will not be documented.
Rescue medication / measures	▪ Paracetamol<br>Paracetamol 500 mg tablets (one pack containing 20 tablets) as rescue medication will be provided to each subject with fever and / or headache at Visit 2. Subjects developing these symptoms after Visit 2 will receive one blister pack with paracetamol at Visit 3.<br>▪ Pure water steam inhalations or salt-water steam inhalations without additives
Investigational medicinal product (IMP)	
IMP 1: Test drug<br>Form<br>Active ingredient(s) per form<br>Mode of administration	Soledum® forte capsules<br>Gastro-resistant capsule, soft<br>Cineole (200 mg per capsule)<br><br>Oral
IMP 2: Reference drug<br>Form<br>Active Ingredient(s) per form<br>Mode of administration	Not applicable
Treatment dosages<br><br>IMP 1: Test drug<br><br><br>IMP 2: Reference drug	 <br>3x1 capsule (corresponding to 3x200 mg cineole) daily starting on day of Visit 2 till Visit 4 (i.e. for up to 15±2 days).<br><br>Not applicable

Primary endpoint	Area under the curve global symptom severity (AUC-WURSS):<br>Derivation of AUC-WURSS is based on mean daily total WURSS-11 scores, which are derived by averaging evening assessment of considered day and morning assessment of the subsequent day per item. The mean item scores will then be summarized to the mean total score of a day. For the AUC-WURSS, the mean daily total WURSS-11 scores will be summed across all days using trapezoidal approximation.
Secondary Endpoints	Wisconsin Upper Respiratory Symptom Survey (WURSS-11):<br>▪ Course of mean daily total score:<br>Course of mean daily total scores from common cold onset to end of study.<br>▪ Course of mean daily group scores (symptom, and QoL domain):<br>Course of mean daily group scores from common cold onset to end of study. Mean daily group scores are derived analogously to mean daily total score.<br>▪ Course of Single Scores (for each question): mean daily scores will be utilized for WURSS-11 questions 2-10.<br>For the first general WURSS-11 question asking for “How sick do you feel today” and last question asking for general assessment of cold compared to the previous day: For these presentations, the worse answer will be chosen for considered day (i.e. maximum score).<br>▪ Symptom severity peak:<br>The symptom severity peak will be assessed based on mean daily total scores<br>▪ Time to remission:<br>Remission is considered as present if the first general WURSS-11 question is ≤1 together with a maximum of 1 symptom scored ≤3 and all other symptoms scored 0 of considered day. The day of first documented remission in relation to day of symptom onset is considered as time to remission.<br>▪ Time to treatment response:<br>Response is defined as a reduction in the total WURSS-11 score by at least 50% of assessed symptom severity peak. The day of first documented treatment response in relation to day of symptom severity peak is considered as time to treatment response.<br>▪ Jackson symptom score (JSS) as assessed by investigator:<br>o Course of total JSS over Visit 2, Visit 3 and Visit 4:<br>The total JSS is derived by summing up eight symptom severity assessments ending up in a score ranging from 0 (no symptoms) to 24 (all symptoms assessed as “Severe”)<br>o Course of single symptom severities over Visit 2, Visit 3, Visit 4<br>▪ Bronchitis severity scale (BSS)<br>o Probability of developing an acute bronchitis as assessed by the percentage of subjects with a total BSS score >2 at least at one study visit (i.e. Visit 2, Visit 3 or Visit 4)<br>o Probability of developing an mild, moderate, severe, very severe acute bronchitis according to the classification made by Kardos [17]<br>▪ Overall judgement on efficacy by investigator<br>▪ Overall judgement on tolerability by investigator / subject<br>▪ Days of sick leave and days of bed rest due to common cold since day of onset<br>▪ Percentage of subjects with rescue medication intake<br>▪ Incidence of adverse events
Statistical plan	<u>Pre-defined analysis sets</u><br>**Screened set (Screen):** The Screened set will comprise all subjects with informed consent. The Screened Set will be used for presentation of disposition and drop-outs.<br><br>**Safety set (SAF):** The safety set will include all subjects, who received at least one IMP dose. The SAF will be used for analysis of safety and tolerability.

Statistical plan (continued)	**Modified Intention-to-treat set (mITT):** The mITT will comprise all subjects,<br>▪ who received at least 4 IMP doses within the first 4 days after Visit 2 with maximum one day without treatment<br>▪ who provide at least 5 valid WURSS-11 assessments within the first 7 illness days after symptom onset (i.e. symptom day 1 to 7) with a maximum of 2 days without assessment, which should not be on two consecutive days<br>▪ without intake of antibiotics after start of IMP treatment due to symptoms associated with upper respiratory tract infections<br>The mITT will be used for the identification of potential confounders as well as for evaluation of primary and secondary efficacy endpoints in a more "real-life" setting.<br><br>**Per protocol Set (PPS):** the PPS includes all subjects in the mITT without any major protocol deviations, which might affect interpretation of efficacy.<br>Major protocol deviations include:<br>▪ Non-compliance with IMP intake, i.e. IMP compliance of <80% or >120%<br>▪ Completed <80% of WURSS-11 assessments<br>▪ Intake of forbidden concomitant medication/therapies<br>▪ Illness was verifiable no common cold (but e.g. influenza)<br>▪ Deviations from eligibility criteria, which might affect course of symptoms<br>The PPS will be used for the evaluation of primary and secondary endpoints under optimal conditions<br>Assignment to analysis sets will be reviewed before data analysis during a data review documenting all assignments and modifications of planned analysis including underlying reasons.<br><br><u>Statistical Analysis</u><br>The scope of the study is purely exploratory. All statistical testing will be done on a nominal significance level of 5% without controlling for multiplicity issues.<br>The statistical evaluation will be carried out by default by tabular display of the number of valid observations (N_{valid}), number of missing observation values (N_{miss}), arithmetic means, standard deviation, minimum, median and maximum. This applies in case of analysis of metric-scaled data, but also in case that metric method seems also reasonable to ordinal-scaled data.<br>Otherwise, categorical data will be displayed in tables by absolute frequencies and their percentages. Two.-sided 95% confidence intervals will be presented, where applicable.<br>Generally, results will be presented by time-to-treatment strata. Time to treatment is derived as the difference between date/time of first IMP intake and date/time of common cold onset.<br><br><u>Strata</u><br>For the analysis the following strata of subjects are planned:<br>▪ Stratum 1: Subjects visit investigator early after feeling sick: Onset of common cold till first IMP administration 0 to ≤16 hours<br>▪ Stratum 2: Subjects visit investigator in regular time after feeling sick: Onset of common cold till first IMP administration: >16 to ≤32 hours<br>▪ Stratum 3: Subjects visit investigator late after feeling sick: Onset of common cold till first IMP administration >32 to ≤48 hours<br>These strata might be adapted due to observed distributions (e.g. by using three equal subject groups from the empirical distribution) during data review before start of the analysis.

Statistical plan (continued)	Comparability of strata<br>To assure the comparability of strata two models will be applied:<br>▪ Model including risk factors resulting from investigation of confounders as covariates, as calculated for the primary endpoint<br>▪ Model utilizing propensity score methods based on risk factors for comparing strata<br>Propensity scores will be calculated by defining logistic regression model with strata as dependent variable and including all identified confounders from the primary endpoint into the model. From this model, the chance of subjects with a specific risk factor combination to enter the respective strata can be obtained. In order to adjust for differences in risks between the strata, inverse probabilities weights (IPW) will be calculated.<br>These analyses will be performed for the mITT as well as for the PPS.<br><br>Analysis of primary endpoint<br>The area under the curve global symptom severity (AUC-WURSS) serves as primary endpoint for this study. The AUC-WURSS is based on mean daily total WURSS-11 scores, which are derived by averaging evening assessment of considered day and morning assessments of the subsequent day per item.<br>The mean item scores will then be summed up to the mean total score of a day. For the AUC-WURSS, the mean daily total WURSS-11 symptom scores will be summed across symptom Day 1 to symptom Day 15 using trapezoidal approximation.<br>Scatter plots will be presented plotting time-to-treatment vs. AUC-WURSS values. In addition, Spearman's rank order correlation coefficient will be presented.<br>General linear models (GLM) will be used for investigating the influence of time to treatment on the AUC-WURSS presenting stratum differences including 95% two-sided confidence intervals. Test on stratum differences will be performed pairwise utilizing the stratum with the longer time-to-treatment interval as reference.<br>For the initial statistical model describing the primary endpoint, baseline score (i.e. total symptom score assessed immediately after common cold onset), gender, age and previous influenza vaccination for the current season will be considered as fixed effects.<br>Additional methods to provide comparability of strata (see above) will be applied as sensitivity analysis.<br><br>Investigation of confounders<br>The investigation of confounders is based on total WURSS-11 score of subjects in the mITT.<br>A linear regression model will be performed using „backward elimination procedure", which includes all potential confounders (e.g. baseline, age, gender, influenza vaccination for the current season, smoking status) as a first step. Only those risk factors should remain, who are significant on a level of $p<0.05$ at each step until all other factors were eliminated. Consequently, the finally selected risk factors can be considered as relevant to the healing process of common cold.<br><br>Analyses of secondary endpoints<br>For the investigation of secondary endpoints representing metric-scaled total scores over time, mixed models for repeated measurements (MMRM) will be applied with baseline (i.e. the earliest value before IMP start) as covariate, and time to treatment, assessment time, time to treatment x assessment time interaction, age, gender, influenza vaccination for the current season as fixed effects and subject as random effect repeated in time. In addition, 95% two-sided confidence limits will be presented overall and by time points for the considered strata as well as stratum differences utilizing the later stratum as reference.

Statistical plan (continued)	Generally, logarithmic transformation will be considered in case of deviations of model residuals from normal distribution. No models will be performed for single-item assessments.<br>To investigate the effect of confounders, the additional models to provide comparability of the strata will be applied following a similar approach described for the primary endpoint.<br>Time to remission in relation to time-to-treatment strata will be investigated by accelerated failure time model (AFT model) for estimating predicted median time and corresponding 95% confidence intervals for the reduction in illness duration (in relation to the maximum time-to-treatment stratum). In addition, the corresponding acceleration factor will be estimated. Within the model, estimation will be done on the natural log scale utilizing log-normal distribution for the error terms.<br>The initial model will include time-to-treatment strata, baseline disease severity (i.e. the earliest WURSS-11 total score before IMP start) and corresponding interaction as well as factor age, gender and previous influenza vaccination. The sensitivity to the effects of confounders will be investigated accordingly.<br>The probability of developing an acute bronchitis will be investigated by logistic regression models utilizing time to treatment, age, gender, influenza vaccination for the current season. In addition, 95% two-sided confidence limits will be presented overall and for each stratum. The sensitivity of observed estimates with respect of confounding factors will be investigated accordingly.<br><br><u>Safety analyses</u><br>For the presentation of safety related incidences, separate analyses will be provided for events starting before / after first intake of IMP.
Randomization	Not applicable
Sample size calculation	Due to the exploratory character of the study aiming to investigate complete common cold episodes including the very early phase under real-life conditions only sparse information is available. As a consequence the clinical trial is designed based on precision of confidence intervals for the total WURSS-11 score rather than on power considerations based on statistical testing.<br>Expecting a balanced distribution of subjects into the 3 time periods of time-to-treatment strata, with 110 subjects the 95% confidence intervals of a width of estimated mean ± 2 points could be estimated assuming a total WURSS-11 symptom score of 15 at symptom severity peak based on published common cold trial results. So estimated total WURSS-11 symptom scores at symptom severity peak will have a precision of 4 score points, which is considered as a substantial precision for clinical interpretation of key results.<br>Assuming that only 66% - 85.5% of screened subjects will experience a cold episode fulfilling all eligibility criteria within a screening phase of up to 7 months, about 400-500 patients are planned to be included into the screening to achieve 330 treated subjects eligible for the analysis (mITT).
Interim analysis	An interim analysis is planned after approx. 60 subjects eligible for the mITT have completed the trial and each stratum has at least approx. 10 subjects to gain a preliminary insight into the drop-out rates, efficacy and heterogeneity of collected data. The results of interim analysis will be summarized in a short report. Any potential changes in conduct or analysis of the study will be documented in the short report together with an evaluation of the impact of planned changes.

Table 1: Flow chart

**Procedures**	**Screening Phase**	**Illness Phase**			
	**Visit 1**		**Visit 2**	**Visit 3**	**Visit 4**
	**Screening**	**Symptom Onset**	**Inclusion Diagnosis, Start of Treatment**	**Control**	**Final (End of Treatment and Clinical Trial)** [1]
	Up to 7 months before Visit 2		Day 1 (≤48 hours after start of eDiary documentation)	Day 4±1	At common cold recovery but latest on Day 15±2
Written informed consent	X				
Check of inclusion / exclusion criteria applicable at Visit 1	X				
SARS-CoV-2 test			X		
Training of eDiary	X				
Check of eDiary entries			X	X	X
Demographic data: age, gender, ethnic origin	X				
Body height and weight (questioning the subject); BMI automatically calculated in eCRF	X				
Previous and concomitant diseases	X		X		
Previous medication			X		
Concomitant medication	X		X	X	X
Inclusion diagnosis for treatment			X		
Check of inclusion / exclusion criteria applicable at Visit 2			X		
Dispense of clinical trial medication			X		
Subjects with fever and / or headache only: Dispense of rescue medication: paracetamol			X	X [2]	
First administration of IMP in the investigator's office and documentation in eDiary			X		

**Procedures**	**Screening Phase**	**Illness Phase**			
	**Visit 1**		**Visit 2**	**Visit 3**	**Visit 4**
	**Screening**	**Symptom Onset**	**Inclusion Diagnosis, Start of Treatment**	**Control**	**Final (End of Treatment and Clinical Trial)** [1]
	Up to 7 months before Visit 2		Day 1 (≤48 hours after start of eDiary documentation)	Day 4±1	At common cold recovery but latest on Day 15±2
Documentation of Confounding variables / parameters:<br>▪ Number of common colds during previous winter season<br>▪ Alcohol consumption<br>▪ Smoking habits<br>▪ Working status	X				
Documentation of confounding variable / parameter:<br>▪ Vaccination against influenza for the coming / current winter season			X		
Current number of common cold episodes within this clinical trial (technical parameter)			X		
Subject: Wisconsin Upper Respiratory Symptom Survey-11 (WURSS-11) in eDiary [3]		←———	———	———	———→
Subject: Administration of IMP and documentation in eDiary			←———	———	———→
Jackson Symptom Score (JSS)			X	X	X
Bronchitis Severity Score (BSS) [4]			X	X	X
Check for common cold complications, e.g. otitis media, pneumonia, tonsillitis			X	X	X
Investigator's global judgement on efficacy					X
Investigator's and subject's global judgement on tolerability					X
Days of sick-leave (for subjects working employed or self-employed) & days of bed rest due to common cold			X	X	X
Vital signs: systolic and diastolic blood pressure, pulse, body temperature, only at Visit 2: oxygen saturation			X	X	X

**Procedures**	**Screening Phase**	**Illness Phase**			
	**Visit 1**		**Visit 2**	**Visit 3**	**Visit 4**
	**Screening**	**Symptom Onset**	**Inclusion Diagnosis, Start of Treatment**	**Control**	**Final (End of Treatment and Clinical Trial)** [1]
	Up to 7 months before Visit 2		Day 1 (≤48 hours after start of eDiary documentation)	Day 4±1	At common cold recovery but latest on Day 15±2
Physical examination	X		X	X	X
For females of childbearing potential:<br>▪ Method of contraception<br>▪ Urine pregnancy test	X		X [5]		X [5]
Check of withdrawal criteria				X	
Check / documentation of adverse events				X	X
Recording of clinical trial termination					X
Drug accountability (IMP & if applicable rescue medication) [6]				X	X

<u>Legend to flow chart</u>

[1] Visit 4 should be performed

- when subject has recovered from common cold (defined as items 1 to 10 in WURSS-11 in the eDiary rated with "0" on two consecutive assessments after Visit 2 in the diary) but latest on Day 15±2,
- in case of premature clinical termination but only in subjects who had at least one administration of IMP. If subject is withdrawn from the clinical trial, Visit 4 shall be performed as soon as possible.

[2] Only in subjects with new onset of fever and / or headache having not received rescue medication paracetamol at Visit 2

[3] Documentation of WURSS-11 in eDiary shall start once the subject feels that he has a common cold. The first entry should be done immediately after the recording of feeling of common cold, then WURSS-11 will be recorded twice daily in the morning and in the evening.

[4] BSS only if subject has a cough. If the subject shows cough according to the corresponding Jackson criterion at Visit 2 or a later visit, the BSS will be evaluated at each subsequent visit.

[5] Visit 2 and Visit 4: urine pregnancy test only

[6] Drug accountability at Visit 3: pill count: IMP and if applicable rescue medication

Drug accountability at Visit 4: pill count and collection of IMP and if applicable rescue medication

3 INTRODUCTION

3.1 Medical background

The common cold is mostly a viral infection of the upper respiratory tract with general and respiratory symptoms such as sneezing, sore throat, and running nose. It is one of the most prevalent illnesses in the world. On average, adults suffer from about two to four episodes per year. Although the disease is usually mild, with symptoms lasting one to two weeks and self-limited, it is one of the leading causes of doctor visits in the United States and missed days from school and work, and has an enormous economic impact. The high incidence along with significant symptomatic and functional impairment makes this syndrome an important health problem. Subjects with common cold may present concomitantly an acute bronchitis [11][17][18].

On average, total cold symptom scores indicate a peak in symptom severity 48 h after initiation of experimental infection and then decrease [11][15]. The symptoms are provoked by a mucus-producing, inflammatory reaction on the infection. Therefore it is desirable to treat subjects early in the course of the disease with an anti-inflammatory and mucolytic agent such as the essential oil cineole, which is the active ingredient of Soledum® Kapseln forte (i.e. trade name in Germany). This product has been used since decades for the treatment of common cold. Further details about this drug are provided in Section 3.2.

3.2 Drug characteristics

Soledum® forte capsules are an anti-inflammatory and mucolytic medicinal product for symptomatic treatment of respiratory colds including bronchitis.

The active ingredient cineole is isolated from eucalyptus oil. It has expectorant, secreto-motoric, weakly hyperemic and local anesthetic effects. Antimicrobial effects were shown in vitro against a broad spectrum of gram positive and gram negative bacteria as well as fungi; an antiviral effect was also shown in vitro.

For information on Soledum® forte capsules regarding preclinical data, clinical investigations, pharmacokinetics, drug interactions, use during pregnancy and lactation and undesirable effects refer to summary of product characteristics (SmPC) [14].

3.2.1 Description of clinical trial rationale

The aim of this clinical trial is to investigate the relation between the timing of treatment onset with cineole and the course of a common cold with or without acute bronchitis. Thus, subjects will be enrolled in the clinical trial before they develop a common cold under investigation. Subsequently, they shall start documentation in a eDiary once they feel having a common cold and visit their investigator as soon as possible again for confirmation of inclusion diagnosis and immediate start of treatment with the investigational medicinal product (IMP). This way a complete common cold episode will be investigated including the very early phase before treatment.

Analogously to another clinical trial with an acute viral respiratory disease investigating whether early start of treatment after symptom onset is more effective [10], this clinical trial was set-up as prospective, open-label, uncontrolled, exploratory, multicenter study.

3.3 Risk-benefit assessment

The major risk in participating in this clinical trial for a subject is that treatment with cineole (Soledum® forte capsules) and rescue medication paracetamol in case of fever and / or headache is not sufficient for symptom control. Nevertheless, the sponsor considers that the benefits prevail the risks due the following reasons:

- The current clinical trial will include male and female adults (aged between 18 and 70 years) with recent onset of common cold without complications for whom immediate antibiotic prescribing is not indicated.
- Women of reproductive potential must agree to use adequate contraception starting latest at the screening visit.
- The IMP (Soledum®), an over-the-counter drug, is approved for the indication, which is investigated in this clinical trial.
- During the 15-day treatment period, all subjects will be closely monitored for safety and tolerability at control visits scheduled 3 (±1), and 14 (±2) days after start of treatment (i.e. at Visit 3 and Visit 4).
- If symptoms get worse and further administration of the IMP is medically not acceptable for the subject, he can be withdrawn from the clinical trial at the discretion of the investigator.
- Participation in the trial is voluntary. Each subject may refuse to participate or withdraw from the trial, at any time, without penalty or loss of benefits to which the subject is otherwise entitled.
- The clinical trial will only be started in September/October 2020 if the situation concerning COVID-19 allows, i.e. there are no relevant travel restrictions in place and there is a very low probability for participants of getting infected. Otherwise the trial will be postponed to September/October 2021. That way the risks concerning COVID-19 for participants are minimized.

3.4 Subsequent treatment and medical attendance after clinical trial termination

The subject's further treatment for "common cold" with or without acute bronchitis - if necessary - is left to the discretion of the investigator.

3.5 References / guidelines

The following references with respect to GCP, legal and ethical matters have been followed:

- Declaration of Helsinki [9]
- Note for Guidance on Clinical Safety Data Management Definitions and Standards for Expedited Reporting: ICH Topic E 2 A [1]
- Note for Guidance on Good Clinical Practice: ICH Topic E 6 (R2) [2]
- Note for Guidance on Statistical Principles for Clinical Trials: ICH Topic E 9 [3]
- Detailed guidance on the collection, verification and presentation of adverse event / reaction reports:'CT3' [4]

4 CLINICAL TRIAL OBJECTIVES

The objective of this clinical trial is to investigate the relation between timing of treatment onset with cineole and course of a common cold with or without acute bronchitis.

5 CLINICAL TRIAL PLAN

5.1 Clinical trial design

This is a Phase IV, prospective, open-label, non-randomized, exploratory multi-center clinical trial to investigate the relation between timing of treatment onset with the IMP and course of a common cold with or without acute bronchitis in adults.

The individual clinical trial duration is up to 8 months with a total of four visits. There will be screening phase of up to 7 months starting at Visit 1 till onset of a common cold. Within the following 48 hours (i.e. start of eDiary documentation, for details refer to Section 9.16) Visit 2 has to be performed, which comprises confirmation of inclusion diagnosis by the investigator and start of the 15-day treatment with the IMP (Soledum® forte capsules). There are two further visits 3 and 14 days after Visit 2, respectively.

Subjects with fever and / or headache will be provided with rescue medication (paracetamol 500 mg tablets) on an “as needed” basis in the event of fever and / or profound / protracted pain.

5.2 Clinical trial centers

The trial will be conducted in approximately 25 suitably qualified trial centers (otolaryngologists (ear, nose, and throat [ENT] specialists), internists, and general practitioners) in Germany.

They must be qualified by education, training and experience as appropriate experts to investigate the drug and to assume responsibility for the proper conduct of the trial in accordance with GCP and applicable regulatory requirements.

5.3 Subject selection, sample size

<u>Subject selection</u>

Female and male adult outpatients visiting their general practitioner, ENT specialist or specialist in internal medicine for any reason will be offered to participate in this clinical trial (i.e. Visit 1). Methods of recruitment may include letters, e-mails, posters, paid advertisements, announcement on the campus and distribution of flyers. Subjects who have given their informed consent will be screened for eligibility. Subjects fulfilling all inclusion and none of the exclusion criteria applicable at Visit 1 will enter the screening phase

They will be instructed on the use of the eDiary and the need to contact the investigator immediately once they have a cold. Once a week they will get a reminder on their smartphone / tablet / computer and will be asked to start documentation in the eDiary via their smartphone / tablet / computer immediately once they feel having a common cold, which is to be documented in the eDiary and set as onset of common cold.

At this time point and subsequently of this twice daily (i.e. in the morning and in the evening) subjects will be asked via reminder to answer all questions of Wisconsin Upper Respiratory Symptom Survey 11 (WURSS-11) in the eDiary. In addition, they should make an appointment (i.e. Visit 2) and visit their investigator as soon as possible. Visit 2 needs to be scheduled earlier than 48 hours after start of eDiary documentation. If this is not possible, the subject is not eligible and

cannot continue for the current illness episode (including eDiary documentation). However, such a subject may continue screening phase for another illness episode.

Sample size

Approximately 400 to 500 adult subjects will be screened in order to get 330 evaluable subjects with confirmed diagnosis of "common cold" under treatment with the IMP.
Further details with respect to sample size calculation are provided in Section 13.11.

5.4 Clinical trial visits

Participation involves four in-person visits within maximum 6.5 months:

Screening phase:

- Visit 1 (up to 7 months before Visit 2): Informed consent, screening of subjects, installation and training of eDiary on subject's smartphone / tablet / computer

Illness phase:

- Up to 48 hours prior 1st IMP intake: Onset common cold, i.e. start of eDiary documentation
- Visit 2 (Day 1): Inclusion diagnosis, start of treatment
- Visit 3 (Day 4± 1): Control visit
- Visit 4 (Day 15± 2): Control visit, end of treatment and clinical trial

Subjects are advised to start the eDiary documentation immediately once they feel that they have a common cold and contact the site for an appointment. The appointment should take place ideally on the same day. The time interval between Visit 1 and Visit 2 depends on the onset of a common cold in the individual subject.

Subjects who do not meet the inclusion diagnosis at Visit 2 (see inclusion criteria no. 7 to 10 in Section 6.2) are allowed to continue screening phase and will become eligible for a new common cold episode after complete recovery of current episode.

5.5 Planned trial duration

The clinical part of the trial is planned to be conducted between September / October 2020 (first subject first visit in the first trial center [FSFV]) and End of March / April 2021 (last subject out in the last trial center [LSO]).

5.6 End of trial

The end of the trial as a whole will be the date when last subject under treatment with IMP had completed the last visit.

6 SUBJECT POPULATION AND INCLUSION / EXCLUSION CRITERIA

6.1 Definition of subjects / inclusion diagnosis

Outpatient adults with the inclusion diagnosis common cold (for definition refer to inclusion criteria no. 7 to 10) at Visit 2 are eligible for clinical trial participation if they meet all of the inclusion and exclusion criteria listed in Sections 6.2 and 6.3 below.

6.2 Inclusion criteria

Visit 1 – Screening

1. Age between 18 and 70 years
2. Recollecting having ≥1 common cold in the last winter season by asking the subject
3. Informed consent, personally dated and signed by the subject and the investigator, and data protection declaration
4. Willingness and ability to use an eDiary
5. Availability of an appropriate device for eDiary data entry, i.e. smartphone, tablet or computer
6. Subject able to follow the instructions given by the investigator

Visit 2 – Inclusion diagnosis, start of treatment

7. Start of eDiary documentation ≤48 hours before first IMP intake
8. Common cold with or without bronchitis clinically diagnosed by the investigator
9. “Yes” to the question “Does it feel to you that you are developing a cold or that you have a cold?”
10. Using Jackson’s criteria, subjects have to report at least a sum score of ≥ 3 in up to the following four cold symptoms:
    - Nasal discharge
    - Nasal obstruction
    - Sneezing
    - Sore throat

6.3 Exclusion criteria

Visit 1 - Screening

1. Any known chronic ear, nose, throat and respiratory tract disease or any other known serious disease that can influence the course of the common cold, e.g. chronic bronchitis (WHO Definition), chronic obstructive pulmonary disease (COPD), bronchial asthma, chronic active allergic rhinitis (e.g. dust mite allergy), chronic rhinosinusitis with or without nasal polyp(s), cystic fibrosis within the past 2 years before Visit 1
2. Pneumonia, pertussis and / or pseudo-croup

3. Long-term treatment (>14 days) with systemically administered antibiotics within the last 7 days before Visit 1
4. Previous SARS-CoV-2 infection confirmed by RT-PCR (based on medical history)
5. Known hypersensitivity to cineole or any of the other compounds of Soledum® forte capsules
6. Known hereditary fructose intolerance
7. Presence of clinically relevant renal and / or liver diseases
8. Any respiratory disease or symptoms related to smoking: asymptomatic smokers are allowed to be included
9. Presence of immunosuppressive state, malignancy (actual, condition after carcinoma less than 2 years without relapse), autoimmune disease(s), AIDS
10. Pregnancy (positive urine pregnancy test) or lactation
11. Female of childbearing potential without medically accepted contraception
12. History or presence of dependency from alcohol or drugs
13. Participation in another clinical study, already terminated or still ongoing, within the last 30 days
14. Known to be, or suspected of being unable to comply with the trial protocol (e.g., no permanent address, history of drug abuse, known to be non-compliant or presenting an unstable psychiatric history)
15. Legal incapacity and / or other circumstances rendering the subject unable to understand the nature, scope and possible impact of the trial
16. Subject in custody by juridical or official order
17. Subject who has difficulties in understanding the language (German) in which the subject information (informed consent form) is given
18. Subjects who are members of the staff of the trial center, staff of the sponsor or the clinical research organization (CRO), the investigator himself or close relatives of the investigator

Visit 2 – Inclusion diagnosis, start of treatment

Same exclusion criteria as for Visit 1 plus the following ones:

19. Start of eDiary documentation longer than 12 h after symptom onset
20. Any known acute ear, nose, throat and respiratory tract disease that can influence the course of the common cold e.g. assured diagnosis of the flu, Covid-19 (acute and previous infection since Visit 1), allergic rhinitis (e.g. allergy to a current available seasonal allergen)
21. Hypoxemia, i.e. $SpO_2 \leq 92\%$
22. Any complications of the common cold that have to be treated with antibiotics
23. Subjects treated with or need for treatment with antibiotics (systemic), glucocorticosteroids (systemic, per inhalation or nasal), β2-mimetics, theophylline, common cold or cough medication (systemic, per inhalation or nasal) within 7 days prior to Visit 2 or at the time of Visit 2

24. Regular treatment (i.e. >2 single doses since onset of common cold) with systemic non-steroidal anti-inflammatory drugs [NSAIDs] / analgesics (except low dose acetylsalicylic acid [ASA]) within 7 days prior to Visit 2 or at the time of Visit 2
25. Drugs without marketing authorization or drugs without marketing authorization in the indication they are used for within 7 days prior to Visit 2 or at the time of Visit 2
26. Angiotensin converting enzyme (ACE) blockers, unless given as long-term therapy over at least 8 weeks on a stable dose prior to enrolment into the study and not causing cough as undesirable effect

6.4 Previous and concomitant medication

6.4.1 Previous medication not permitted

Within 7 days prior to Visit 2

- Antibiotics (systemic)
- Glucocorticosteroids (systemic, per inhalation or nasal)
- β2-mimetics
- Theophylline
- Common cold or cough medication, e.g. expectorants (e.g. acetylcysteine) / mucolytics, drops / liquids for nasal decongestion, antitussives, cineole, or other essential oils (systemic or per inhalation), phytotherapeutics (including pharmacy-only lozenges)
- Treatment with systemic NSAIDs / analgesics (except low dose ASA)
- Drugs without marketing authorization or drugs without marketing authorization in the indication they are used for

Angiotensin converting enzyme (ACE) blockers are also not permitted, unless given as long-term therapy over at least 8 weeks on a stable dose prior to enrolment into the study and not causing cough as undesirable effect.

6.4.2 Concomitant medication not permitted

Any not permitted previous medication listed in Section 6.4.1 (cineole as IMP and paracetamol as rescue medication excluded) is also not allowed in the period from Visit 2 to Visit 4.

6.4.3 Permitted medication and measures

Other continuing pharmacotherapy not listed above in constant dose for diseases other than common cold is allowed.

Pure water or salt water steam inhalation without additives are also allowed.

Note: Further measures (tea, gargling etc.) with non-pharmaceutical products will not be documented.

Note: in subjects who receive an influenza vaccination during the screening period (i.e. between Visit 1 and Visit 2) and suffer from side effects which could be interpreted as an upcoming common cold (e.g. feverish feeling), these symptoms should not be considered as an onset of symptoms for a “normal” common cold episode in the sense of this protocol for up to 5 days after the vaccination.

The subjects should not start the eDiary documentation or come to Visit 2 (or at least be re-enrolled by the site in case the subjects come to Visit 2).

6.5 Declaration regarding inclusion of subjects who are possibly dependent on the sponsor or the investigator

Subjects, who are members of the staff of the clinical trial center, sponsor or involved CRO, the investigator himself or close relatives are not allowed to participate in this clinical trial.

7 CLINICAL TRIAL THERAPY

7.1 Description of clinical trial medication and control

7.1.1 Identity of clinical trial medication

The drug that is used in this clinical trial is Soledum® forte capsules. The following table provides relevant information of the trading goods of Soledum® forte capsules used in this clinical trial.

Table 2: Identity of the used drug in this clinical trial

	Test product
Brand name	German original name: Soledum® Kapseln forte<br>English translation: Soledum® forte capsules
Manufacturer	Klosterfrau Berlin GmbH, Motzener Str. 41, 12277 Berlin, Germany
ATC-code	R05CA13
Pharmacological group	Expectorants
Form	Enteric-coated capsule, soft
Color	Colorless
Route of administration	Oral
Active ingredient per form	1 capsule contains 200 mg cineole
Non-active ingredients (excipients)	Medium chain triglycerides, sorbitol solution 70% (not crystallising) (Ph. Eur.), gelatine, glycerol 85%, ethyl cellulose, ammonia solution 28%, oleic acid, sodium alginate, stearic acid, candelilla wax
Shelf life	5 years*
Special storage recommendations	Do not store above 30.0°C

* Date will be assigned during the process of labelling of the trial medication and reported in the trial report

7.1.2 Manufacturing and packaging of Soledum® forte capsules

The manufacturer of Soledum® forte capsules is Klosterfrau Berlin GmbH (Motzener Str. 41, 12277 Berlin, Germany), which belongs as the sponsor Cassella-med GmbH & Co. KG to the Klosterfrau Health Care Group. Klosterfrau Berlin GmbH confirms the manufacturing in compliance with the guidelines of Good Manufacturing Practice (GMP) by releasing the product to the market. Certificates of analysis, a GMP certificate and the release certificate will be made available by Klosterfrau Berlin GmbH.

7.1.3 Packaging and Labelling

Investigational medicinal products (IMPs)

The external service provider Hubertus Apotheke (Hubertus Apotheke am Salzufer, Salzufer 13/14, 10587 Berlin) will be responsible for the labelling of the trial medication in accordance with the applicable regulations and guidelines. The IMP contains 4 blisters with 25 capsules each which are sufficient for 17 days (scheduled treatment plus reserve medication). Each IMP medication pack will be labeled with one label and one tear-off label.

Upon dispensing the IMP medication pack to the subject, the tear-off label is detached and stuck on the drug account form.

The text on the labels will be in German language.

Rescue medication

Rescue medication packs show the labeling information provided for the marketed product used in this trial and will be re-labeled in the same way as the IMP.

7.2 Release of trial medication

The release of the trial medication will be done by Hubertus Apotheke, Berlin.

7.3 Shipment and drug accountability

The clinical trial medication may not be dispatched to the clinical trial site before all ethical and legal requirements have been fulfilled. The CRO will check, if all necessary requirements are met and will inform the Hubertus Apotheke accordingly and instruct them to send the IMP to the trial site.

The responsibility for the clinical trial medication accountability at the clinical trial site rests with the investigator. The investigator should maintain records of the delivery of products (IMPs and rescue medication) to the trial site, the inventory at the site, the use by each subject, and the return of unused products. After completion of reconciliation by monitor the unused medication and empty medication packs (IMP and rescue medication) will be returned to and destroyed by the contracted service provider Hubertus Apotheke, Berlin. A written proof of destruction will be made available to the sponsor.

7.4 Storage

The investigator must store the clinical trial medication (IMP and rescue medication) in accordance with the manufacturer’s instructions, i.e. in a dry place, not above 30.0°C and protected from light. At the clinical trial center, the IMP and rescue medication should be stored in a safe place (access restricted to clinical trial personnel only). This trial medication (IMP and rescue medication) must not be used after the expiry date has elapsed.

7.5 Drug supply per subject

At Visit 2 (after assessment of eligibility), each subject will receive one IMP medication pack containing 100 capsules (scheduled supply plus reserve medication) sufficient for the complete trial duration. Additionally, each subject with fever and / or headache will receive one rescue medication pack containing 20 paracetamol 500 mg tablets (rescue medication). In case of new onset of fever and / or headache after Visit 2, rescue medication will be dispensed at Visit 3.

The investigator will explain the correct use of the IMP and - if applicable - rescue medication to each subject before dispensing the medication pack(s). The subject will be instructed to keep an electronic diary (eDiary) documenting date and time of IMP administration and - if applicable - intake of rescue medication (if needed), see Section 9.16.

Subjects are asked to bring back the IMP and - if applicable - rescue medication pack (empty blister / blister with unused capsules / tablets) to the trial site at Visit 3 and Visit 4 for compliance checks (see Section 7.7).

7.6 Dosage and route of administration, duration of treatment

IMP

In this clinical trial, the IMP will be administered three times a day (t.i.d.: 3x1 capsules). The chosen dosing regimen is in accordance with the dosing recommendations of the summary of products characteristics for adults.

The scheduled treatment duration is 15 days (±2 days). Treatment starts on the day of Visit 2 (Day 1) and ends on the day of Visit 4 (Day 15±2). Subjects, who recover from common cold before Visit 4, may terminate the administration of the IMP, once the first 10 WURSS-11 questions were rated by the subject “0” on two consecutive assessments in the eDiary.

Subjects will be instructed to administer the IMP t.i.d. except for the first and the last day of treatment, as specified below:

- On Day 1 (start of treatment), the IMP will be administered once (1x1 capsule), twice (2x1 capsule) or three times (3x1 capsule) by the subject depending on the time of Visit 2. The first dose will be administered at the trial site, after the subject was found eligible by the investigator. Possible further administration(s) on Day 1 and on the following days until end of treatment will take place at subject’s home.
- On each subsequent day (Day 2 until the day before Visit 4), the IMP will be administered t.i.d. (3x1 capsule), (i.e. in the morning, at noon and in the evening).
- On the day of Visit 4 (Day 15±2), the IMP will only be administered once in the morning (1x1 capsule, last dose).

IMP has to be swallowed unchewed with sufficient fluid (preferably one glass of water [200 mL]), but no hot beverage, approximately half an hour before a meal. Subjects with a sensitive stomach are recommended to take the IMP during their regular meals.

Subjects will be instructed to document in the eDiary the total number of capsules taken per day (see Section 9.16).

Rescue medication

The single dose is limited to 500 - 1,000 mg paracetamol (1-2 tablets). The maximum daily dose is limited to 1,500 mg (3 tablets / day) not exceeding the recommendations of the summary of product characteristics (SmPC) / package insert. The interval between two consecutive dosages should be at least 6 hours. The investigator should instruct the subjects at Visit 2 to limit the use of paracetamol to an absolute minimum and only in the event of fever and / or profound / protracted pain.

7.7 Compliance

Apart from the first dose in the investigator's office, the IMP will be self-administered by the subject at home. Drug administration (IMP and if applicable rescue medication) will be documented in an eDiary (Section 9.16). Each investigator has to judge whether the subject is able to use the IMP and if applicable rescue medication properly and to make regular visits to the clinical trial site.

Compliance to visit schedule

A subject is regarded compliant to visit schedule (according to protocol) if Visit 3 takes place at Day 4 (±1 day) and Visit 4 on Day 15 (±2 days). Same applies in case of premature recovery (see Sections 8.1 and 9.15) if Visit 4 is performed earlier.

Compliance to IMP treatment

Treatment compliance will be checked based on the returned IMP medication pack (number of used / unused capsules and subject's documented intake of capsules in the eDiary (see Section 9.16)).

Any discrepancies between actual and expected amount of returned capsules must be discussed with the subject at the time of Visit 3 and Visit 4, and an explanation must be documented.

A compliance of at least 80% is required, meaning documented intake of at least 80% of the planned number of capsules.

Drug Accountability

The clinical trial staff must ensure that all unused medication (empty and non-dispensed clinical trial drug – IMP and if applicable rescue medication) are retained by the site's clinical trial personnel in a safe location and capsules / tablets are counted. This is to be documented in the eCRF and on the Drug Account Form.

7.8 Blinding, unblinding, emergency codes

Not applicable – this is an open-label clinical trial. All subjects eligible at Visit 2 will receive the IMP Soledum® forte capsules.

8 CONDUCT OF CLINICAL TRIAL AND ASSESSMENTS

8.1 Planned visits (Visit 1 - Visit 4)

In total, four in-person visits are scheduled within up to 8 months per subject. Clinical trial procedures and assessments by visits are listed in the following sections.

Steps to be taken in case of early termination of IMP treatment (dropout) are provided in Section 11.2.

Visit 1 (up to 7 months before Visit 2): Screening and enrolment

- Subject information, subject's written informed consent
- Demographic data: age, gender, ethnic origin
  only females: childbearing potential:
  if yes: method of contraception
  if no: date of menopause (at least year) or other measures
- Body height and weight (questioning the subject); body mass index (BMI) will be automatically calculated in the eCRF
- Confounding variables / parameters:
  - Number of common colds during previous winter season
  - Smoking habits
  - Alcohol consumption
  - Working status
- Previous and concomitant diseases
- Concomitant medication
- Physical examination
- *Females of childbearing potential only*: urine pregnancy test
- Check of inclusion and exclusion criteria applicable at Visit 1
- Training of eDiary

Visit 2 (Day 1): inclusion diagnosis, start of treatment

Prior to dispense of the IMP / rescue medication, the investigator must ensure that the subject meets the inclusion diagnosis "common cold" and all of the corresponding inclusion and none of the exclusion criteria (refer to Sections 6.2 and 6.3).

- Current number of common cold episodes within this clinical trial (technical parameter)
- Check of eDiary entries
- Days of sick-leave (for subjects working employed or self-employed) & days of bed rest due to common cold since day of onset
- Confounding variable / parameter:
  - Vaccination against influenza for the coming / current winter season
- Jackson Symptom Score (JSS)
- Inclusion diagnosis: common cold (for details see Section 6.2, inclusion criteria no. 7 to 10)

- Common cold complications, e.g. otitis media, pneumonia, tonsillitis
- Pharyngeal swab for SARS-CoV-2 test via RT-PCR or antigen rapid test
- *Only if cough is present*: Bronchitis Severity Score (BSS)
- Previous and concomitant diseases (update)
- Previous and concomitant medication (update)
- Physical examination
- Vital signs: systolic and diastolic blood pressure, pulse, body temperature, oxygen saturation [SpO2] of the blood
- *Females of childbearing potential only*: urine pregnancy test
- Check of inclusion and exclusion criteria applicable at Visit 2
- Dispense of clinical trial medication (IMP)
- First administration of IMP in the investigator's office and documentation in eDiary
- *Subjects with fever and / or headache only*: Dispense of rescue medication: paracetamol

Note: Visit 2 must not take place on the same day as Visit 1. For details refer to Section 9.16.

Visit 3 (Day 4±1): control visit

- Check of eDiary entries
- Jackson Symptom Score (JSS)
- Common cold complications, e.g. otitis media, pneumonia, tonsillitis
- *Only if cough is present is present at this or a previous visit:* Bronchitis Severity Score (BSS)
- Concomitant medication (changes)
- Physical examination
- Vital signs: systolic and diastolic blood pressure, pulse, body temperature
- Check of withdrawal criteria
- Check / documentation of adverse events
- Days of sick-leave (for subjects working employed or self-employed) & days of bed rest due to common cold since previous visit
- Recording of clinical trial termination (only if applicable)
- Drug accountability (pill count: IMP & if applicable rescue medication)
- *Only subjects with new onset of fever and / or headache having not received rescue medication at Visit 2*: Dispense of rescue medication: paracetamol

Visit 4 (at common cold recovery but latest Day 15±2): final visit: end of treatment and clinical trial

- Check of eDiary entries
- Jackson Symptom Score (JSS)
- Common cold complications, e.g. otitis media, pneumonia, tonsillitis
- *Only if cough is present at this or a previous visit:* Bronchitis Severity Score (BSS)
- Concomitant medication (changes)
- Physical examination

- Vital signs: systolic and diastolic blood pressure, pulse, body temperature
- *Females of childbearing potential only*: urine pregnancy test
- Check / documentation of adverse events
- Investigator's global judgement on efficacy
- Investigator's and subject's global judgment on tolerability
- Days of sick-leave (for subjects working employed or self-employed) & days of bed rest due to common cold since previous visit
- Recording of clinical trial termination
- Drug accountability (pill count and collection: IMP & if applicable rescue medication)

Visit 4 may be performed earlier and if applicable also instead of Visit 3, when the subject has recovered from common cold before Day 15±2 but only in subjects who had at least one administration of IMP. A subject is recovered in case he rated the items 1 to 10 of the WURSS-11 in the eDiary with "0" on two consecutive assessments after Visit 2.

8.2 Premature withdrawal

In the case of premature withdrawal once treatment with IMP was started, the subject, wherever possible, will be submitted as soon as possible to a discontinuation visit; hereto Visit 4 clinical trial assessments will be performed. For further details regarding discontinuation criteria please refer to Section 11.2.

8.3 Flow chart

A flow chart of clinical trial assessments is provided on page 20.

9 METHODOLOGY

9.1 Subject information and informed consent

All relevant information on the clinical trial is summarized in an integrated subject information sheet and informed consent form (ICF) (see Section 14.5). The ICF will also include the consent for handling of personal data in concurrence with the General Data Protection Regulation (GDPR). Based on this subject information sheet, the investigator must explain all aspects of the clinical trial to each subject prior to subject's participation in the trial (i.e. before any examinations and procedures associated with the selection for the trial are performed not considered "standard of care" or any trial-specific data was recorded on trial-specific forms).

Only if the subject voluntarily agrees to sign the informed consent form and has done so, he may enter the trial. In the event that informed consent is obtained on the date that baseline trial procedures are performed, subject's clinical records must clearly show that informed consent was obtained before these procedures (documentation of clock time of collecting informed consent and clock time of start of first study specific screening activities). For details refer Section 14.5.2.

9.2 Demographic data

Scheduled time of documentation: Visit 1 (screening)

The following demographic data will be recorded in the subject's medical file and transcribed into the eCRF:

- Age at screening (years) and year of birth
- Gender [female, male]
- Ethnic origin (Caucasian [white], Black, Asian, other)
- *Only females:* childbearing potential
  - if yes, method of contraception (for details see below)
  - if no, postmenopausal: date of menopause (month, year or at least year) or other measures (sterilization, hysterectomy, bilateral oophorectomy)

  Females of childbearing potential must commit to use a medically accepted method of contraception / birth control from Visit 1 and during the entire duration of the clinical trial (i.e. up to Visit 4 / Day 15±2).

  Medically accepted methods of contraception / birth control comprise the following measures:
  - Established use of oral, injected, or implanted hormonal methods of contraception
  - Placement of an intrauterine device (IUD)
  - Sexual abstinence or vasectomized partner (if he is the only sexual partner of the female and success of vasectomy was medically proven)
  - Barrier form of contraception (diaphragm, cervical cap, male condom, female condom, spermicidal foam, sponges, and film vaginal spermicides)

9.3 Confounding variables / parameters

The following confounding variables / parameters will be recorded in the subject's medical file and transcribed into the eCRF:

Scheduled time of documentation: Visit 1 (screening)

- Number of common colds during previous winter season (September till March)
- Alcohol consumption (g per day on average)
- Smoking habits (smoker: start year of smoking, average number of cigarettes per day; ex-smoker: start and stop year of smoking, average number of cigarettes per day up to the day of quitting smoking, date of quitting; non-smoker)

  Definitions (according to [20]):
  - Smoker: at least 1 cigarette / day or 5 cigarettes / week or 1 pack / month
  - Ex-smoker: Subject does currently not smoke, also not occasionally
- Working status: (self-)employed /student or not employed

Scheduled time of documentation: Visit 2 (inclusion diagnosis, start of treatment)

- Vaccination against influenza for the coming / current winter season (yes, no)

9.4 Confirmation of inclusion diagnosis

Scheduled time of documentation: Visit 2 (inclusion diagnosis, start of treatment)

Because objective signs of an early common cold are usually lacking, clinical trials of common cold treatment rely primarily on subjective data from the study participant [15].

The definition of inclusion diagnosis "common cold" in this clinical trial follows closely the criteria employed in another clinical trial in 2011: Answer had to be "Yes" to investigator's question "Do you think that you have a cold? or "Do you think you coming down with a cold?" In addition, four of eight Jackson criteria (nasal discharge, nasal obstruction, sneezing and/or sore throat) had to result in a sum score of 2 or higher with 0=absent, 1=mild, 2=moderate, 3=severe, and a symptom onset within 36 hours [12].

At Visit 2 / Day 1, the investigator will check whether the subject has a common cold with or without acute bronchitis (= upper respiratory tract infection) defined as the presence of the following criteria at Visit 2:

- Subject currently feels having a common cold
- Selected Jackson criteria: sum score ≥3 score points:
  - Nasal discharge
  - Nasal obstruction
  - Sneezing
  - Sore throat

  Scoring for each symptom: 0 = none / not present; 1 = mild; 2 = moderate; 3 = severe.
- Onset of common cold ≤48 hours (i.e. start of eDiary documentation) prior to first administration of the IMP
- Clinical confirmation of inclusion diagnosis "common cold" by the investigator

Onset of common cold is defined as the time point of subject's first documentation of feeling to have a common cold in the eDiary prior to Visit 2.

The following information will be recorded in the subject's medical file and transcribed into the eCRF:

- Subject currently feels having a common cold (yes, no)
- Individual score value for each Jackson criterion and sum score (score points)
- Date and time of subject's first documentation of feeling to have a common cold in the eDiary and time till first administration: documentation in source data only in order to verify inclusion diagnosis but no transcription into eCRF. Data will be transferred for analysis from eDiary.
- Confirmation of diagnosis 'common cold' by the investigator (yes, no)

9.5 Jackson symptom score

The Jackson symptom scale was developed in the nineteen-fifties and comprises the following eight symptoms:

- Nasal discharge
- Nasal obstruction
- Sneezing
- Sore throat
- Headache
- Malaise
- Chilliness
- Cough

Scoring for each symptom: 0 = none / not present; 1 = mild; 2 = moderate; 3 = severe [12][16].

Thus, the sum score of the eight Jackson criteria may result in 0 to maximum 24 score points.

Note: At Visit 2 and all subsequent visits all 8 criteria have to be assessed, even if the first four criteria are relevant for assessment of the inclusion diagnosis common cold (refer to Section 9.4).

9.6 Previous and concomitant diseases

Scheduled time of documentation: Visit 1 (screening), Visit 2 (inclusion diagnosis, start of treatment)

The following information on subject's previous and concomitant diseases will be recorded in the subject's medical file and transcribed into the eCRF:

- Any ear, nose, throat (ENT) and respiratory tract disease within 1 year prior to Visit 1 and thereafter till Visit 2;
- Previous SARS-CoV-2 infection confirmed by RT-PCR before Visit 1
- Other concomitant diseases / disorders that did not terminate at least 2 years prior to Visit 1 and those thereafter till Visit 2;
- Surgeries within 2 years prior to Visit 1 and thereafter till Visit 2.

Documentation comprises the medical term, start date and - if applicable - stop date or indicating the disease / disorder as “ongoing”. Sources may be the subject file and / or actually collected information by questioning the subject.

Note: The inclusion diagnosis “common cold” is to be transcribed into the Visit 2 module of the eCRF but not again in the module “Previous and concomitant diseases”.

Detailed instructions on the differentiation between medical history and adverse events (AEs) can be found in Section 10.

9.7 Previous and concomitant medication

Scheduled time of documentation: Visit 1 (screening), Visit 2 (inclusion diagnosis, start of treatment), Visit 3 and Visit 4

The following information on subject’s previous and concomitant medication will be recorded in the subject’s medical file and transcribed into the eCRF:

- Previous medication that was stopped within 4 weeks prior to Visit 2 (to be checked at Visit 2)
- Concomitant medication at Visit 1 and Visit 2.
- From Visit 3 to 4, it will be documented whether there are any changes in the use of concomitant medication. If yes, changes have to be specified too.
- Documentation comprises trade name (preferably for medication with more than one active ingredient) or International Non-proprietary Name (INN), start date and - if applicable - stop date or indicating the medication as “ongoing”, indication, dose, unit, frequency, formulation and route of administration.

Notes:

- IUDs (except copper coils) are to be transcribed into the module “Previous and concomitant medication” of the eCRF.
- Rescue medication paracetamol has to be transcribed into the module “Previous and concomitant medication” of the eCRF. Separate entries for different dosages are required.
- Any form of (salt) water steam inhalations is to be transcribed into the module “Previous and concomitant medication” of the eCRF.
- Further measures (tea, gargling etc.) with non-pharmaceutical products will not to be documented.

9.8 Physical examination

Scheduled time of documentation: Visit 1 (screening), Visit 2 (inclusion diagnosis, start of treatment), Visit 3 and Visit 4

The following information on physical examination will be recorded in the subject’s medical file and transcribed into the eCRF:

Physical examinations will be performed (by inspection, palpation, and auscultation) by an investigator at the trial center. At the minimum, the following aspects/regions need to be assessed as normal / abnormal (including specification): general appearance, skin, eyes, ears / nose / throat, head and neck, lungs, heart, abdomen, lymph nodes, musculoskeletal system, and neurologic findings.

The clinically significant findings of the physical examination at Visit 1 and / or Visit 2 have to be recorded as concomitant disease and changes thereafter as adverse event.

Note: Findings of “common cold” with or without acute bronchitis are not to be recorded as a finding of the physical examination.

9.9 Body weight and height

Scheduled time of measurement: Visit 1 (screening)

Subjects will be asked about their height (cm) and weight (kg). Information will be recorded in the subject’s medical file and transcribed into the eCRF.

Subject’s body mass index (BMI, kg/m^2) will be automatically calculated in the eCRF.

9.10 Vital signs (blood pressure, pulse rate, body temperature, oxygen saturation)

Scheduled times of measurement: Visit 2, Visit 3, and Visit 4

Vital signs (blood pressure, pulse rate, body temperature) will be measured by a member of the investigator’s team. The same method of measurement should be used for the subject during the trial. Results will be recorded in the subject’s medical file and transcribed into the eCRF.

- Blood pressure and pulse rate

  Subject’s systolic / diastolic blood pressure and pulse rate will be measured in sitting position after at least 5 minutes rest in millimeter of mercury (mmHg) and beats per minute (bpm), respectively, using an oscillometric device. If possible, the same arm will be used for all measurements in one subject.

- Body temperature (tympanic)

  Subject’s body temperature in degrees centigrade (°C) will be measured tympanic in the right or left ear or on the forehead using a thermometer with digital display.

- Only at Visit 2: Oxygen saturation [SpO_2%] of the blood

  Oxygen saturation will be measured by pulse oximetry in percent. The pulse oximeter consists of a small device that clips to the body (typically a finger or earlobe).

9.11 SARS-CoV-2 test

Scheduled times of measurement: Visit 2

Sample collection for SARS-CoV-2 virus will be done by a pharyngeal swab. The sample is sent for RT-PCR qualitative analysis to a local laboratory or an antigen rapid test is performed directly at the site.

9.12 Urine pregnancy test

Scheduled times of measurement: Visit 1, Visit 2, Visit 4

A urine pregnancy test (dipstick) will be performed by the clinical trial personnel in all female subjects of childbearing potential:

The tests will be evaluated by the clinical trial staff and the results (negative, positive) documented in the medical file of each subject and transcribed into the eCRF.

9.12.1 Investigator's global judgement on efficacy

Scheduled times of measurement: Visit 4

The investigator will assess the global response to treatment using the following 5-point rating scale (German text provided in square brackets) at Visit 4:

0 = very good *[sehr gut]*

1 = good *[gut]*

2 = moderate *[mäßig]*

3 = poor *[schlecht]*

4 = very poor *[sehr schlecht]*

9.13 Investigator's and subject's global judgement on tolerability

Scheduled times of measurement: Visit 4

The investigator and the subject (each separately) will assess the overall (global) tolerability of the IMP using the following 5-point rating scale (German text provided in square brackets) at Visit 4:

0 = very good *[sehr gut]*

1 = good *[gut]*

2 = moderate *[mäßig]*

3 = poor *[schlecht]*

4 = very poor *[sehr schlecht]*

In case of a rating of 3 or worse by either the investigator or the subject, an adverse event should be evaluated and recorded.

9.14 Bronchitis Severity Score (BSS)

Scheduled times of measurement: If the subject shows cough according to the corresponding Jackson criterion at Visit 2 or a later visit, the BSS will be evaluated at each subsequent visit (Visit 3, Visit 4)

The BSS is an appropriate outcome measure in acute bronchitis which has been successfully used in many clinical studies since its introduction in 1996. The scale comprises the five most important features of acute bronchitis:

- Cough
- Sputum production (expectoration)
- Rales on auscultation
- Chest pain on coughing
- Dyspnea

The BSS is an instrument which combines objective and subjective items, because the assessment is based on the investigator's clinical evaluation in conjunction with the subjective

feedback of the subject. Each constituent of the BSS is assessed by the investigator using a 5-point verbal rating scale ranging from 0 to 4:

0 = absent *[kein / nicht vorhanden]*

1 = mild *[leicht]*

2 = moderate *[mäßig]*

3 = severe *[stark]*

4 = very severe *[sehr stark]*

The total score of BSS is the sum of the five ratings with a maximum of 20 points [17].

The relationship between BSS score and severity of acute bronchitis is as follows:

- BSS total score of 0: No acute bronchitis
- BSS total score of 1 to 2: Acute bronchitis unlikely
- BSS total score of 3 to 7: Mild acute bronchitis
- BSS total score of 8 to 12: Moderate acute bronchitis
- BSS total score of 13 to 17: Severe acute bronchitis
- BSS total score of 18 to 20: Very severe acute bronchitis

9.15 Wisconsin Upper Respiratory Symptom Survey (WURSS-11)

Scheduled times of measurement: Twice daily in the eDiary starting when subject indicates feeling to have a cold until recovery or latest till the morning of Visit 4

The WURSS was developed to provide a standardized measure for evaluating the negative consequences of a common cold. It is a health related quality of life instrument that represents the symptomatic and functional dimensions that are important to persons suffering from a common cold. It is based on self-assessment because neither accepted criteria nor adequate tests are available to diagnose upper respiratory tract infection and because the vast majority of common cold treatments are taken without professional input after self-diagnosis [11]. The WURSS is widely used in clinical trials in subjects with upper respiratory tract infections as an evaluative illness-specific quality of life instrument to assess the negative impact of acute upper respiratory infection [12].

The WURSS-11 used in this clinical trial is shorter version of WURSS-21 intending to reduce questionnaire completion time and increase response rate. WURSS-11 preserved its reliability and domain structure [19].

In addition, to the first question about feeling sick, the indicated dimensions (items) in WURSS-11 include nasal (runny nose, plugged nose, sneezing), throat (cough, sore throat, scratchy throat), and quality of life (feeling tired, think clearly, accomplish daily activities) [19]. The severity of each item can be rated on a 0 (Not sick / Do not have this symptom / Not at all) to 7 (Severely/ Severe) Likert scale. Finally, there is a question regarding the change of common cold compared to previous day. Subject's rating shall consider the preceding 24 hours [13].

The WURSS-11 will be presented to the subjects in local language (German) in the eDiary via their smartphone / tablet / computer for completion at the time of the subject's first documentation of feeling having a common cold and subsequently twice daily (morning and evening) until the

subject's recovery but latest till the morning of the day of Visit 4. A subject is recovered in case he rated the items 1-10 of the WURSS-11 in the eDiary with "0" on two consecutive assessments.

9.16 Electronic diary (eDiary) / electronic patient reported outcomes (ePRO)

Scheduled times of record keeping in the diary: Visit 1 (screening) to Visit 4

A web-based system for electronic collection of subject data (eDiary) will be used in this trial. Subjects participating in this trial must be capable of using the system on their own device (e.g. smartphone, tablet or computer) and be comfortable with it after a short period of training provided by the investigator / site staff at Visit 1.

Subjects will be asked to keep the eDiary for the entire trial period to document the following items:

Sending of alerts

- From one week after Visit 1 till subject's start of documentation in eDiary
  - Once weekly always on the same day (i.e. weekday of Visit 1) a reminder will be sent to the subject's smartphone / tablet / computer to document immediately when he feels first time having a common cold and answering WURSS-11. Documentation can be started at any day time.

  Note: Subjects consulting their investigator for any kind of acute respiratory tract infection at Visit 1 will receive the first alert three weeks after Visit 1, to ensure that the current respiratory tract infection is resolved. In addition, the subjects will be instructed not to start eDiary documentation if the respiratory tract infection has not completely disappeared within three weeks after Visit 1.
- From subject's start of documentation in eDiary until the subject's recovery from common cold but latest till the morning of Visit 4
  - Twice daily (morning, evening) a link will be sent to answer WURSS-11 once the subject had started documentation in the eDiary (see above). After first IMP intake in the investigator's office at Visit 2, the evening link comprises the request to document the intake of the IMP and rescue medication for the current day.
  - Alerts will be sent in the morning and in the evening and up to two times again at given intervals if the subject has not done the eDiary documentation. After the last morning and evening reminder, respectively, there is another hour to complete the eDiary documentation. Thereafter, the entry will be locked for the respective time.

Documentation in eDiary

- Confirmation, that subject feels the first time having a common cold plus answering WURSS-11 including automatic registration of recording time (time stamp) which will be considered as time point of onset of common cold. In addition, the subject will be asked to document in the eDiary when he felt the first time having a common cold which must not be no longer than 12 hours before recording time. If this interval is exceeded, the current common cold episode cannot be considered for final inclusion of subject. In this case the eDiary will be locked for the next 3 weeks. Thereafter, the eDiary can be used for documentation of another common cold episode.
- Subsequently answering WURSS-11 (morning, evening) including automatic registration of recording time (time stamp)

- First administration of IMP including clock time (in the investigator’s office at Visit 2)
- Administration of IMP for the current day (number of capsules taken in the morning, noon and evening) including recording of clock time: record for the whole day in the evening
- Administration of rescue medication for the current day (number of tablets taken, clock time): record for the whole day in the evening

The subject’s eDiary documentation must be reviewed for completeness by the investigator via web at Visits 2, 3 and 4 and checked for drug compliance.

Note: Subjects who do not meet the inclusion diagnosis at Visit 2 (see inclusion criteria no. 7 to 10 and “Verification of inclusion diagnosis” above) shall stop documentation in the eDiary for the current illness episode but are allowed to continue screening phase and might become eligible for a new common cold episode after complete recovery of current episode. A new message will be sent and entries for the next episode will be possible 21 days after the last eDiary entry of the previous episode. The screening no. for the respective subject allocated at Visit 1 will be kept.

9.17 Adverse events (AE)

Scheduled times of measurement: Visit 3, Visit 4

Adverse events (AEs) spontaneously reported by the subject or observed by the investigator have to be recorded.

To obtain comparable documentation on AEs, the investigator will ask the subject the following open, standard question, in his native language at each visit:

“Has anything changed in your state of health since you last came to see me?”

For further details regarding recording of AEs, see Section 10.

10 ADVERSE EVENTS (AE)

10.1 Definitions

10.1.1 Adverse event (AE)

An adverse event (AE) is defined as any untoward medical occurrence in a clinical trial subject to whom an IMP / rescue medication has been administered and which does not necessarily have a causal relationship with this treatment.

An AE can therefore be any unfavorable and unintended sign (including an abnormal laboratory finding), symptom, or disease temporarily associated with the use of an IMP / rescue medication, whether or not considered related to the IMP / rescue medication. Abnormalities of laboratory parameters, vital signs or electrocardiogram (ECG) abnormalities are to be recorded as AEs only if they are medically relevant, requiring corrective treatment, leading to discontinuation or fulfil the criterion of seriousness.

In addition, the following points will be considered:

- Conditions that started between Visit 1 and first IMP administration at Visit 2 will be recorded as previous or concomitant disease, respectively. Same applies for surgeries. Thus, they will not be considered as AEs.
- Worsening and / or onset of common cold symptoms after first IMP administration will not be recorded as AE.
- Worsening and / or onset of concomitant acute bronchitis symptoms will not be recorded as AE.
- A hospitalization between Visit 2 and Visit 4 but planned before Visit 2 will be reported as AE but not as serious adverse event (SAE).

10.1.2 Adverse reactions (ARs)

An adverse reaction (AR) is any untoward and unintended response to an IMP / rescue medication irrespective of the dose administered.

All AEs judged by either the reporting investigator or the sponsor as having a reasonable causal relationship to a medicinal product qualify as ARs. The expression “reasonable causal relationship” means to convey in general that there is evidence or argument to suggest a causal relationship.

10.1.3 Serious adverse events (SAEs)

A SAE is any untoward medical occurrence or effect that at any dose:

- results in death,
- is life-threatening,
- Note: Life-threatening is considered any AE or AR in which the subject was at risk of death at the time of the event; it does not refer to an event which hypothetically might have caused death if it were more severe.
- requires hospitalization or prolongation of existing hospitalization,
- results in persistent or significant disability or incapacity,

- is a congenital anomaly or birth defect.

An important medical event that is not immediately life-threatening or will result in death or hospitalization, but which may jeopardize the subject or may require medical intervention to prevent one of the outcomes listed above, should be reported as “serious” as well.

Any suspected transmission of an infectious agent via a medicinal product is considered serious and should be assessed under the category of medically important events in the absence of other seriousness criteria.

Emergency room and day or night observational visits are not considered serious until one of the above criteria is met. Prolongation of hospitalization is defined as a hospital stay prolonged by at least one day.

10.1.4 Unexpected adverse reactions (UARs)

An unexpected adverse reaction (UAR) is an AR, the nature or severity of which is not consistent with the information available on the IMP / rescue medication (e.g. Investigator's Brochure for an unapproved IMP or summary of product characteristics [SmPC] for an authorized IMP and rescue medication).

10.1.5 Suspected unexpected serious adverse reactions (SUSARs)

All suspected ARs related to an IMP / rescue medication which occur in the concerned clinical trial and that are both unexpected and serious are classified as suspected unexpected serious adverse reactions (SUSARs).

SUSARs are subject to expedited reporting as specified in Section 10.3.

10.1.6 Definition and assessment of the intensity

Regardless of the classification of an AE as “serious” or “non-serious”, its intensity must be assessed according to the following categories:

- Mild: Symptoms do not interfere with routine activities.
- Moderate: The adverse event causes discomfort and affects the subject’s normal activities / interferes with routine activities.
- Severe: The adverse event causes considerable interference with the subject’s usual activities, e.g. inability to work, necessity to discontinue the clinical trial medication.

It should be noted that a severe AE does not necessarily have to be serious in nature and that a SAE does not need to be severe.

10.1.7 Classification and coding of the causal relationship

The investigator will be responsible for the classification of a causal relationship of an AE separately to the IMP and rescue medication using the following categories:

Term	Description
Related	There is a reasonable causal relationship, which means that there is evidence to suggest a causal relationship. The adverse event could medically (pharmacologically / clinically) be attributed to the IMP / rescue medication under clinical trial in this protocol.
Not related	There is no reasonable causal relationship, which means that there is no evidence to suggest a causal relationship. The adverse event could not medically (pharmacologically / clinically) be attributed to the IMP / rescue medication under clinical trial in this protocol. A reasonable alternative explanation must be given.

10.2 Documentation and reporting of AEs

At each visit the Investigator will assess any occurred AE. AEs communicated by the subject or by the subject's relatives or delegates through phone calls, letters, faxes or e-mails will also be recorded. In these cases the investigator will try to obtain medical confirmation and assessment of the occurred AE.

Any AE occurring during the clinical trial has to be documented, including the following data:

- Description of the AE (symptoms, diagnosis) in medical terms, not as reported by clinical trial subject
- Seriousness: classification of the AE as "serious" or "not serious"
- Intensity (mild, moderate, severe)
- Date of first occurrence (start date and clock time) and stop date / ongoing
- Frequency (single event, repeatedly, permanently)
- Action taken (none, concomitant medication, IMP definitely discontinued, rescue medication definitely discontinued clinical trial discontinued permanently for the subject, hospitalization, other, unknown: [multiple answers possible])
- Causal relationship separately for IMP and rescue medication (according to Section 10.1.7).
- Outcome of AE
  - Resolved: Fully recovered, or by medical or surgical treatment the condition has returned to the level observed at the first clinical trial related activity after the signed ICF.
  - Resolved with sequelae: As a result of the AE, the subject suffers from persistent and significant disability / incapacity (e.g. became blind, deaf, paralyzed). Any AE recovered with sequelae should be rated as an SAE.
  - Not resolved (detailed description required)
  - Fatal
  - Unknown

The general obligation to record AEs by the investigator ends at last study visit per subject (usually Visit 4).

The investigator is expected to record also any IMP / rescue medication related AE occurred during 1 week after the administration of the last IMP / rescue medication dose, which becomes aware to the investigator.
Note: It is not the obligation of the investigator to contact the subjects actively and ask for AR occurred in this time period.

10.3 Documentation and reporting of SAEs

10.3.1 Reporting duties of the investigator

Any serious adverse event (SAE) which occurs during the course of the clinical trial whether or not related to the IMP must be reported **immediately (i.e. within 24 hours)** to

Marketing Authorisation Holder / Sponsor:		Subcontracted CRO Safety Manager:
Cassella-med GmbH & Co KG	**AND**	Dr. med. Reinhard Nibler
Global Product Safety Department		Dr. Nibler & Partner, Ärzte
Gereonsmühlengasse 1		Fuerstenriederstrasse 105
50670 Köln, Germany		80686 Muenchen

Reporting of SAEs will be done via eCRF. In case that an SAE is documented in the eCRF, an e-mail notification is triggered automatically and immediately sent to the Global Product Safety Department of Cassella-med GmbH & Co. KG and to Dr. Nibler & Partner. A report with the details of the documented SAE(s) will be available for download or printout in the eCRF.

The preliminary information must contain at least the following data:

- Name of the reporting investigator
- Identification of the clinical trial: clinical trial code or title, respectively, IMP / rescue medication.
- Identification of the subject: assigned screening no., gender and year of birth.
- Description of the SAE: symptoms / diagnosis, therapeutic measures taken, outcome as far as already known, causal relationship.

The investigator should provide a follow-up of the SAE in the eCRF within a reasonable time of the initial reporting, depending on duration of the event and availability of evaluations and reports of third parties. The original of the SAE report form will remain in the subject file at the clinical trial center.

In the event of death of a clinical trial subject, the investigator shall supply the Global Product Safety Department of the sponsor and Dr. Nibler & Partner with any additional information requested. Personal data must be made pseudonymous prior to being communicated by using the identification code of the clinical trial subject.

Any further information and supporting documentation that become available (copies of laboratory reports, tests, procedures, autopsy evidence of the cause of death, etc.) shall be provided by the

investigator through follow-up in the eCRF to the Global Product Safety Department of the sponsor and Dr. Nibler & Partner (see above) for a detailed description and a final evaluation of the case.

Only if there is no access to internet or eCRF for such a long time period that the above mentioned reporting timeline cannot be maintained, the SAE shall documented on a paper form and transmitted via email (preferred communication route) or telephone. In the case of a phone report, this preliminary report is to be followed by a written report as soon as possible to the Global Product Safety Department of the sponsor and Dr. Nibler & Partner (see above) if the SAE entry in eCRF is still not possible. Otherwise the documentation shall switch to eCRF entry.

10.3.2 Reporting duties of the sponsor

All SUSARs with the IMP will be expedited reported to the competent authorities (CAs) by the sponsor and reporting to the ethics committees (EC) / Institutional Review Boards (IRBs) will be done by Dr. Nibler & Partner on behalf of the sponsor. All SUSARs with the rescue medication will be expedited reported by Dr. Nibler & Partner.

Reporting must be done following general and local rules and procedures and within these deadlines after the first knowledge

- Fatal and life threatening SUSARs: within a maximum of **7 days**
- All other SUSARs: within a maximum of **15 days**

Relevant follow-up information of fatal or life-threatening SUSARs will be communicated subsequently within an additional **8 days**.

Any circumstances which require a review of the risk-benefit assessment of the IMP will be reported to the CAs concerned and to the ECs concerned promptly in accordance with local laws and the Commission Directive 2001/20/EC [6].

10.4 Follow-up of (S)AE

All AEs not resolved at the end of the clinical trial irrespective of severity and whether serious or not, are to be followed up by the investigator until they have satisfactorily subsided or have stabilized to such an extent that further marked improvement can no longer be expected.

10.5 Handling of pregnancies

The investigator will collect pregnancy information on any female subject, who becomes pregnant between Visit 2 and Visit 4. The investigator has to record pregnancy information in the eCRF within 2 weeks of acknowledgment of a subject’s pregnancy. The eCRF entry of pregnancy will trigger automatically an e-mail notification which will be sent immediately to the Global Product Safety Department of Cassella-med GmbH & Co. KG and to Dr. Nibler & Partner. The subject’s state of health will also be followed to determine the outcome of the pregnancy. Information on the status of the mother and child will be forwarded to the sponsor. Generally, follow-up will be no longer than 3 months following the estimated delivery date. Any premature termination of the pregnancy will be reported.

While pregnancy itself is not considered to be an AE or SAE, any pregnancy complication or elective termination of a pregnancy for medical reasons will be recorded as an AE or SAE.

A spontaneous abortion is always considered to be an SAE and will be reported as such. Furthermore, any SAE occurring as a result of a post-clinical-trial pregnancy which is considered reasonably related to the IMP / rescue medication by the investigator will be reported to the sponsor.

11 STOPPING RULES AND DISCONTINUATION CRITERIA

11.1 Replacement of drop-outs

Subjects who discontinue the clinical trial participation before Visit 2 and before the entire end of the screening phase of the trial are considered to be screening failures and will be replaced. Subjects who discontinue the clinical trial participation prematurely after first clinical trial drug administration will not be replaced. However screening will be continued until 330 evaluable subjects have started treatment with IMP.

11.2 Discontinuation criteria for individual subjects

The subject is entitled to terminate the clinical trial at any time without giving any reasons and without experiencing any disadvantages. However, the investigator has to try to find out and document the reasons for a premature discontinuation.

<u>Screening failures (i.e. drop-out till Visit 2)</u>

Subjects are considered to be a screening failure, if one of the following constellations occurs:

- Withdrawal of consent prior to start of treatment with IMP
- eDiary documentation not started during the screening phase
- Eligibility not given at Visit 1 or Visit 2 (i.e. at least one inclusion criterion not met or exclusion criterion met)
- Subject still in screening phase at end of clinical part of the trial
- SARS-CoV-2 infection confirmed by RT-PCR

<u>Drop-outs after Visit 2</u>

Once treatment of common cold with IMP has started subjects have to be withdrawn from treatment with IMP and if applicable also from the clinical trial in case of:

- Withdrawal of consent
- Appearance of non-tolerable adverse events *
- Appearance of exclusion criteria (e.g. SARS-CoV-2 infection) / common cold complications, which concern the safety of the subject
- Appearance of dyspnea and/or fever (>38,5°C) for more than 3 consecutive days and/or purulent or bloody sputum
- Insufficient efficacy which is medically not acceptable for this subject *
- Administration of systemic antibiotics
- Occurrence of pregnancy up to last administration of IMP *
- Poor compliance
- Subject lost to follow-up
- Any situation judged by the investigator to be harmful for the subject *

In case of prematurely withdrawn from the clinical trial after Visit 2, the subject, if available, will be asked as soon as possible to attend a discontinuation visit (i.e. Visit 4) for safety reasons. In

addition, the subject has to return to the investigator any left-over IMP and - if applicable - rescue medication. An AE that has not recovered by Visit 4 will be followed up until resolved or stabilized to such extent that no further improvement can be expected.

Notes: In cases highlighted with an asterisk (*) the subject shall continue the clinical trial as scheduled until Visit 4 but without administration of IMP if he doesn't use another medication (apart from rescue medication) for treatment of common cold and is willing to continue documentation in eDiary.

In case of premature trial discontinuation Visit 4 shall be performed earlier (i.e. as soon as possible) and if applicable also instead of Visit 3.

11.3 Premature discontinuation of the clinical trial at a clinical trial site

The sponsor / CRO may discontinue the clinical trial at one clinical trial center for the following reasons:

- The investigator is unable to include an adequate number of subjects in a given period.
- The investigator fails to comply with the requirements of the clinical trial protocol, with GCP standards and applicable regulatory requirements.
- The investigator inadequately co-operates with the sponsor / CRO.
- The investigator withdraws his consent for participation.

If the clinical trial is prematurely discontinued or suspended for any reason, the investigator / institution should promptly inform the clinical trial subjects, should assure appropriate therapy and follow-up for the subjects. On behalf of the sponsor the CRO Pharmalog will inform the ethics committees as well as regulatory and local authorities.

11.4 Premature discontinuation of the entire clinical trial

Taking into account the requirements to assess the benefit / risk ratio, the sponsor (project manager of the clinical trial) may terminate the entire clinical trial at any time.

It is therefore necessary for the investigator to report to the project manager of the CRO immediately all health hazards occurred and / or every single subject who prematurely terminated the clinical trial, and to inform them continuously about any new findings concerning the clinical trial medication.

If a clinical trial is prematurely terminated or suspended, Pharmalog - on behalf of the sponsor - will promptly inform the investigators, and the regulatory authorities of the early termination or suspension and the reason(s) for the early termination or suspension. Investigators have to inform all subjects including those still in the screening phase between Visit 1 and Visit 2. The ethics committees should also be informed promptly and provided the reason(s) for early termination or suspension of the clinical trial by the sponsor, as specified by the applicable regulatory requirement(s).

12 RECORDING AND ARCHIVING OF DATA

12.1 Data recording in the eCRF

The investigator is responsible for the accuracy, compliance to protocol, completeness and legibility of data documented in the eCRF.

Documentation

All data will be entered in the eCRF using a validated electronic remote data capture system (EDC), by the investigators or authorized staff within 5 working days from data collection in the source documents. The EDC system MARVIN, provided by the company XClinical, complies with all relevant regulations of the United States Food and Drug Administration (U.S. FDA), in particular with Part 11 of Title 21 of the Code of Federal Regulations (21 CFR Part 11: Electronic records; Electronic signatures). For the purpose of this clinical trial the term eCRF will refer to the used EDC system MARVIN.

A hardcopy snapshot of the eCRF (blank CRF) will be provided for guidance of the investigators. No data capture will be done on this paper document.

At the end of the trial and database lock, the recruiting site will receive a compact disc read-only memory (CD-ROM) with the generated subject data as a portable document format (PDF) file.

Corrections

The eCRF system will include auto-edit checks (e.g. range checks, conditional checks, etc.) which show up during the data entry process by the site. Related specifications will be detailed in the Data Validation Plan.

Manual queries will be created by the monitor, data manager or medical reviewer directly in the eCRF upon data verification or medical review, respectively. Queries have to be answered by the sites directly in the eCRF.

All changes of the subjects' eCRF-data will be recorded in an audit trail.

Subject identification form

The subject identification form contains a listing of the identities of all subjects enrolled (i.e. informed consent form signed) in the clinical trial.

Screening failures at Visit 1

Only the following data will be transcribed into the eCRF.

- Demographic data
- Screening failure reason (e.g. specified exclusion criterion etc.)
- Confounding factors

Screening failures at Visit 2 without later inclusion in the clinical trial

Only the following data will be transcribed into the eCRF.

- Visit 1: all data

- Visit 2 data: Date of Visit 2, number of current common cold episode and screening failure reason (e.g. specified exclusion criterion etc.)

Eligible patients at Visit 1 without later inclusion in the clinical trial

The following data will be transcribed into the eCRF:

- Visit 1: all data

12.2 Archiving

12.2.1 Medical files (investigator site)

The investigator has to retain the medical files of his clinical trial subjects in accordance with national legislation and in accordance with the maximum period of time permitted by the hospital, institution or private practice.

Prior to initiation of the clinical trial center, an 'Investigator's Site File" (ISF) will be compiled for the clinical trial center by the CRO. The investigator and / or the institution, respectively, has to maintain and update the essential clinical trial documents specified in chapter 8 of the Guideline for Good Clinical Practice E6 [2], and as required by applicable national regulatory provisions.

As the identification of the subjects and the respective subject's records in each clinical trial center has to be guaranteed, essential documents relating to this clinical trial have to be retained for at least 10 years after clinical trial completion or for a longer period, if required by other applicable requirements or by an agreement between the sponsor and the investigator (according to Commission Directive 2005/28/EC [7]).

The investigator should take measures to prevent accidental or premature destruction of records. If archiving can no longer be maintained at the site, the investigator will notify the sponsor.

12.2.2 Trial Master File (sponsor site)

All essential documents (including the eCRFs during the trial, and CD-ROM / digital versatile disc [DVD] with the generated subject data as a PDF file after clinical trial completion) are the basis for the audit by the sponsor's independent auditor and for the inspection by the CA(s). Essential documents have to be archived in a way that ensures that they are readily available, upon request, to the CAs. During the planning and the conduct of the clinical trial, the TMF with respect to its tasks is located at the CRO and will be transferred to the sponsor at the end of the trial.

Essential documents relating to this clinical trial have to be retained for at least 10 years after clinical trial completion or early discontinuation (according to Commission Directive 2005/28/EC [7]) or for a longer period,.

12.3 Quality assurance

Risk based approach

A risk-based approach is implemented for all phases of this clinical trial (e.g., planning, conduction, statistical analysis). It is laid down in the Risk Quality Management Plan in accordance with the Guideline for Good Clinical Practice: ICH Topic E6(R2) [2].

The implemented quality approach for the trial, a summary of important deviations from predefined tolerance limits and the actions taken will be described in the clinical trial report.

Monitoring

Monitoring and source data verification (SDV) will be performed according to the standard operating procedures (SOPs) of Pharmalog. Data management, including plausibility checks, will be conducted according to ICH-guidelines. Corrections of implausibilities will be completed and documented prior to data base lock. The locking of the data base will be documented.

Data management

Manual Queries will be created by the monitor, data manager or medical reviewer directly in eCRF upon data verification or by medical review. Queries have to be answered by the sites directly in the eCRF. All changes of the subjects' eCRF-data will be recorded in an audit trail.

12.3.1 Monitoring

The clinical monitor will visit the investigator during the clinical trial to review the progress and conduct of the clinical trial and check the eCRF for completeness and accuracy according to GCP and relevant national law. The eDiary entries will be checked for completeness. All personnel involved in the clinical trial should be prepared to make time available to discuss the data with the clinical monitor. The frequency of monitoring visits will be detailed in the monitoring plan. In addition to regular monitoring visits, remote monitoring and centralized monitoring will be conducted.

Prior to the start of the clinical trial, the clinical monitor will verify that all prerequisites at the clinical trial site are met and will discuss the protocol and eCRF with the investigator and any personnel who will have major responsibilities in the clinical trial. After start of the clinical trial a trained investigator will be responsible for training of any new trial personnel.

During the clinical trial, the monitor will verify the correct conduct of the clinical trial, including subject's informed consent, handling of clinical trial medication, documentation, source data and reporting of AE.

Inconsistencies subsequently identified during validation of the database will be referred back to the investigator as data clarification forms.

In accordance with GCP, the clinical monitor will verify the data recorded in the eCRF against the investigator's source documentation (source data verification).

12.3.2 Source data / source data verification (SDV)

All assessments and investigations have to be entered primarily in the patient record which is considered as source data. In case the investigator uses an electronic data system and no audit trail is implemented for the subjects' medical records, he is requested to provide a printout of the electronic medical records of the clinical trial participants. By his dated signature the investigator confirms consistency between printout and original (electronic) file and that the printout represents the whole period since the last print-out. Also the monitor will date and sign the print-outs accordingly. Study data will be entered into a validated and 21 CFR Part 11 compliant eCRF at the investigator's site (see Section 12.1). In addition, the eDiaries used in this clinical trial are regarded as source documents.

The investigator will permit the monitor, the sponsor representative, the auditor(s), the IRB / EC, and the inspector(s) appointed by regulatory authority(ies) direct access to source documents (original records or certified copies) for verification of data entered into the eCRF, guaranteeing confidentiality of data. By signing the written informed consent, each clinical trial subject documents his permission to SDV.

For the following parameters 100%-SDV will be performed; i.e. for every single subject:

- Subject identification
- Subject written informed consent obtained
- Subject's age (year of birth)
- Visit dates
- Subject eligibility (inclusion and exclusion criteria)
- Reported AEs

For all other parameters a 20%-source data verification will be performed, i.e. 100% - SDV for every fifth subject. Further details will be laid down in the monitoring plan.

Subject data recorded in the eCRF will be source verified by the monitor and documented in the eCRF.

12.3.3 Audit

The purpose of a sponsor's audit, which is independent of and separate from routine monitoring or quality control functions, is to evaluate the clinical trial conduct and the compliance with the protocol, SOPs, ICH-GCP, and the applicable regulatory requirements. An audit for quality assurance may be carried out at all participating clinical trial sites and institutions.

13 STATISTICAL METHODS

13.1 Statistical analysis plan

All planned analyses will be described in a statistical analysis plan (SAP), which will be finalized before first subject will be included for Visit 2. It reflects the planned analysis at start of the study. All modifications from the planned analysis, which might become necessary e.g. due to new evidence or unforeseen data issues, will be clearly documented in the study report together with time and rationale for this change. Substantial changes, which become necessary before data base lock will also be documented in a SAP update.

13.2 General considerations

Statistical analysis will be performed using Statistical Analysis System (SAS) software.

The scope of the study is purely exploratory. All statistical testing will be done on a nominal significance level of 5% without controlling for multiplicity issues.

The statistical evaluation will be carried out by default by tabular display of the number of valid observations (N_{valid}), number of missing observation values (N_{miss}), arithmetic means, standard deviation, minimum, median and maximum. This applies in case of analysis of metric-scaled data, but also in case that metric method seems also reasonable to ordinal-scaled data.

Otherwise, categorical data will be displayed in tables by absolute frequencies and their percentages. Two-sided 95% confidence intervals will be presented, where applicable.

Demographic data, confounding factors and baseline characteristics will be analysed by default summary statistics. To investigate the potential selection bias induced by the recruitment process, descriptive summaries for demographic data and confounding factors will be provided for the Screened Set and compared with the corresponding figures for the mITT in a descriptive manner.

13.3 Analysis sets

Screened set (Screen)

The screened set will comprise all subjects with informed consent. The Screened Set will be used for presentation of disposition and drop-outs.

A descriptive analysis of screening failures based on the Screened Set is planned to create drop-out pattern information that could be of value for the planning of future studies. More specifically, this comprises the percentage of subjects with

- consent withdrawal during screening phase
- eligibility not given at Visit 1
- no start of eDiary documentation during screening phase
- report of illness onset during screening phase but eligibility not given at Visit 2.

The documented reasons for being a screening failure at Visit 1 will be presented.

For subjects reporting an illness onset but failing eligibility at Visit 2, the respective reason, results of assessments performed at Visit 2 (i.e. BSS, Jackson), confounding variables and e-diary entries up to Visit 2 will be evaluated descriptively.

<u>Safety set (SAF)</u>

The safety set will include all subjects, who received at least one IMP dose.

The SAF will be used for analysis of safety and tolerability.

<u>Modified Intention-to-treat set (mITT)</u>

The mITT will comprise all subjects,

- who received at least 4 IMP doses within the first 4 days after Visit 2 with maximum one day without treatment
- who provide at least 5 valid WURSS-11 assessments within the first 7 illness days after symptom onset (i.e. symptom day 1 to 7) with a maximum of 2 days without assessment, which should not be on two consecutive days.

  <u>Explanatory Note:</u> This definition should ensure a sufficient number of assessments in the critical early phase of a common cold to achieve reasonable imputation results (see section 13.10). The first 7 days after symptom onset are considered irrespective of the termination status of a subject, e.g. if WURSS-11 assessment stopped on symptom day 5 and the cold is still present, then this subject will be excluded from the mITT due to missing WURSS-11 assessments on symptom day 6 and 7. On the contrary, missing assessments after a common cold recovery before symptom day 7 will not lead to an exclusion from mITT.
- without intake of antibiotics after start of IMP treatment due to symptoms associated with upper respiratory tract infections

The mITT will be used to for the identification of potential confounders as well as for evaluation of primary and secondary efficacy endpoints in a more “real-life” setting.

<u>Per protocol Set (PPS)</u>

The PPS includes all subjects in the mITT without any major protocol deviations, which might affect interpretation of efficacy.

Major protocol deviations include:

- Non-compliance with IMP intake, i.e. IMP compliance of <80% or >120%
- Completed <80% of WURSS-11 assessments
- Intake of forbidden concomitant medication/therapies,
- Illness was verifiable no common cold (but e.g. influenza),
- Deviations from eligibility criteria, which might affect course of symptoms.

The PPS will be used for the evaluation of primary and secondary endpoints under optimal conditions.

Assignment to analysis sets will be reviewed before data analysis during a data review documenting all assignments and modifications of planned analysis including underlying reasons.

All exceptional cases and problems and the subjects disposition will be considered and resolved in a Data Review Meeting (DRM) after all data have been verified, coded and entered into the data base, but before database closure and before starting the analyses.

13.4 Definition of strata and comparability

13.4.1 Definition of strata

Generally, efficacy results will be presented by time-to-treatment strata. Time to treatment is derived as the difference between date / time of first IMP intake and date / time of common cold onset.

Analysis of eDiary entries will generally be presented by symptom day, i.e. the day of symptom onset will be set as symptom Day 1. As a consequence, presentations by symptoms days will generally range from Day 1 to Day 17.

For the analysis the following strata of subjects are planned:

- Stratum 1: Subjects visit investigator early after feeling sick: Onset of common cold till first IMP administration 0 to ≤16 hours
- Stratum 2: Subjects visit investigator in regular time after feeling sick: Onset of common cold till first IMP administration: >16 to ≤32 hours
- Stratum 3: Subjects visit investigator late after feeling sick: Onset of common cold till first IMP administration >32 to ≤48 hours

These strata might be adapted due to observed distributions (e.g. by using three equal subject groups from the empirical distribution) during data review before start of the analysis.

13.4.2 Comparability of strata

To assure the comparability of strata two models will be applied:

- Model including risk factors resulting from investigation of confounders as covariates, as calculated for the primary endpoint (see Section 13.5.3)
- Model utilizing propensity score methods based on the same risk factors for comparing strata.

Propensity scores will be calculated by defining logistic regression model with strata as dependent variable and including all identified confounder from the primary endpoint (see Section 13.5.3) into the model. From this model, the chance of subjects with a specific risk factor combination to enter the respective strata can be obtained. In order to adjust for differences in risks between the strata, inverse probabilities weights (IPW) will be calculated.

These analyses will be performed for the mITT as well as for the PPS.

13.5 Primary endpoint (mITT, PPS)

13.5.1 Definition of primary endpoint

The area under the curve global symptom severity (AUC-WURSS) serves as primary endpoint for this study.

The AUC-WURSS is based on mean daily total WURSS-11 scores, which are derived by averaging evening assessment of considered symptom day and morning assessments of the subsequent day per item.

The mean item scores will then be summed up to the mean total score of a day. For the AUC-WURSS, the mean daily total WURSS-11 symptom scores will be summed across symptom Day 1 to symptom Day 15 using trapezoidal approximation.

13.5.2 Analysis of primary endpoint – basic approach

Scatter plots will be presented plotting time-to-treatment vs. AUC-WURSS values. In addition, Spearman's rank order correlation coefficient will be presented.

General linear models (GLM) will be used for investigating the influence of time to treatment on the AUC-WURSS presenting stratum differences including 95% two-sided confidence intervals.

Test on stratum differences will be performed pairwise utilizing the stratum with the longer time-to-treatment interval as reference.

Generally, logarithmic transformation will be considered in case of GLM-residuals are deviating from normal distribution. In case of substantial deviation from the linearity assumption of log-transformed data, a change to a suitable non-linear model will be considered.

For the initial statistical model describing the primary endpoint, baseline score (i.e. total symptom score assessed immediately after common cold onset), gender, age and previous influenza vaccination for the current season will be considered as fixed effects.

In addition, the following confounder analysis (see Section 13.5.3) will be performed.

13.5.3 Confounders adjustment approach

The investigation of confounders is based on total WURSS-11 score of subjects in the mITT.

A linear regression model will be performed using „backward elimination procedure", which includes all potential confounders (e.g. baseline, age, gender, influenza vaccination for the current season, and smoking status) as a first step. Only those risk factors should remain, who are significant on a level of $p<0.05$ at each step until all other factors were eliminated. Consequently, the finally selected risk factors can be considered as relevant to the healing process of common cold and will be applied for all primary and secondary efficacy endpoints as part of the sensitivity analysis.

13.5.4 Propensity score approach

In addition, a propensity score approach will be applied (see Section 13.4.2) for the analysis of the primary endpoint in order to adjust for risk differences between strata.

13.6 Secondary endpoints

13.6.1 Secondary endpoint definitions

Based on the WURSS-11 assessments, the following secondary endpoints will be investigated:

- Course of mean daily total score:
  Mean daily total score are derived per symptom day as described in the section above and will be analyzed per symptom day.

- Course of mean daily group scores (symptom, and QoL domain):
  Mean daily group scores are derived analogously to mean daily total score by considering only WURSS-11 questions 2 to 8 for the symptom domain and WURSS-11 questions 9 and 10 for the QoL domain.
- Course of single item scores:
  For WURSS-11 questions 2-10 mean daily single scores will be derived per symptom day by averaging evening and morning assessments of the subsequent day.
  For the first general WURSS-11 question asking for "How sick do you feel today" and last question asking for general assessment of cold compared to the previous day: For each symptom day, the worse answer will be chosen (i.e. maximum score), accordingly.
- Symptom severity peak:
  For a subject, the symptom severity peak will be derived as the highest observed mean daily total WURSS-11 score.
- Time to remission:
  Remission is considered as present, if WURSS-11 question 1 is ≤1 together with a maximum of 1 symptom scored ≤3 and all other symptoms scored 0 of considered day. The day of first documented remission in relation to day of symptom onset (i.e. symptom day) is considered as time to remission. A subject will be censored at the time of last measurement, if no remission was observed before.
- Time to treatment response:
  Response is defined as a reduction in the total WURSS-11 score by at least 50% of assessed symptom severity peak. The symptom day of first documented treatment response in relation to day of symptom severity peak is considered as time of treatment response.
  A subject will be censored at the time of last measurement, if symptom severity peak was reached before last documented value but no response was achieved afterwards.
  A subject will not be included in the evaluation of time to treatment response, if symptom severity peak was assessed at the last observed symptom day as such data situation indicates that the symptom severity peak has not yet been achieved.

Based on the Jackson symptom assessment, the following secondary endpoints will be investigated:

- Course of total Jackson symptom score (JSS) over Visit 2, Visit 3 and Visit 4:
  The total JSS is derived by summing up eight symptom severity assessments ending up in a score ranging from 0 (no symptoms) to 24 (all symptoms assessed as "Severe").
- Course of single symptom severities over Visit 2, Visit 3, Visit 4

Further endpoints will be evaluated:

- Probability of developing an acute bronchitis as assessed by the percentage of subjects with a total BSS score >2 at least at one study visit (i.e. Visit 2, Visit 3 or Visit 4)
- Probability of developing an mild, moderate, severe, very severe acute bronchitis according to the classification made by Kardos [17]
- Overall judgement on efficacy by investigator

- Overall judgement on tolerability by investigator / subject
- Days of sick leave and days of bed rest due to common cold since day of onset
- Percentage of subjects with rescue medication intake during the course of illness

Furthermore, the incidence of adverse events will be described for all SAF subjects.

13.6.2 Analyses of secondary endpoints – metric scales

For the investigation of secondary endpoints representing metric-scaled total scores over time, mixed models for repeated measurements (MMRM) will be applied with baseline (i.e. the earliest value before IMP start) as covariate, and time to treatment, assessment time, time to treatment x assessment time interaction, age, gender, influenza vaccination for the current season as fixed effects and subject as random effect repeated in time. In addition, 95% two-sided confidence limits will be presented overall and by time points for the considered strata as well as stratum differences utilizing the later stratum as reference. Generally, logarithmic transformation will be considered in case of deviations of model residuals from normal distribution.

To investigate the effect of confounders, the additional models to provide comparability of the strata will be applied following a similar approach as described for the primary endpoint and defined in Section 13.4.2.

Only descriptive summaries will be presented for single-item assessments of the WURSS-11 as well as Jackson symptom assessments.

13.6.3 Analyses of secondary endpoints – time to event

Time to remission in relation to time-to-treatment strata will be investigated by accelerated failure time model (AFT model) for estimating predicted median time and corresponding 95% confidence intervals for the reduction in illness duration (in relation to the maximum time-to-treatment stratum). In addition, the corresponding acceleration factor will be estimated.

Within the model, estimation will be done on the natural log scale utilizing log-normal distribution for the error terms. The initial model will include time-to-treatment strata, baseline disease severity (i.e. the earliest WURSS-11 total score before IMP start) and corresponding interaction as well as factors age, gender and previous influenza vaccination.

The sensitivity to the effects of confounders will be investigated following a similar approach as described for the primary endpoint.

The probability of developing an acute bronchitis will be investigated by logistic regression models utilizing time to treatment, age, gender, influenza vaccination for the current season. In addition, 95% two-sided confidence limits will be presented overall and for each stratum. The sensitivity of observed estimates with respect of confounding factors will be investigated following a similar approach as described for the primary endpoint and defined in Section 13.4.2.

13.7 Baseline parameters / confounders

Appropriate descriptive summaries will be provided overall and by time to treatment strata for baseline parameters including confounding parameters.

For this study, complications will be act as confounding factor to the course of common cold. Accordingly, the incidence of common cold complications will be presented overall and by time to treatment strata.

13.8 Compliance and exposure

Appropriate descriptive summaries will be provided overall and by time to treatment strata for compliance and exposure for the mITT.

Compliance with WURSS-11 assessments will be derived as the number of valid WURSS-assessments divided by the expected number of assessments in percentages. No assessments are expected after the day of recovery. If no date of recovery is available, the estimated day of recovery truncated at symptom Day 17 will be used for compliance assessment as defined in Section 13.10.

Compliance with IMP intake will be derived as the number of capsules intake divided by the expected number of capsules in percentages. Therefore no IMP intake is expected after the day of recovery. If no date of recovery is available, the estimated day of recovery truncated at symptom Day 17 will be used as defined in Section 13.10.

Exposure will be described by the duration of IMP intake and mean daily dose.

Further details will be specified in the SAP.

13.9 Interim analyses

An interim analysis is planned after approx. 60 subjects eligible for the mITT have completed the trial and each stratum has at least approx. 10 subjects to gain a preliminary insight into the drop-out rates, efficacy and heterogeneity of collected data. Further details will be specified in the SAP.

The results of interim analysis will be summarized in a short report. Any potential changes in conduct or analysis of the study will be documented in the short report together with an evaluation of the impact of planned changes.

13.10 Handling of missing data

Missing data will be imputed by the following procedures:

<u>Safety data</u>

Missing safety event data as evaluated by the SAF set will not be imputed.

<u>Estimated day of symptom recovery</u>

Due to the subjective character as well as the heterogeneous etiology of a common cold, a high inter-subject variability is assumed. However, the natural course of a common cold is reported as self-limiting disease increasing up to a severity peak followed by a constant decline to recovery.

As a consequence population based imputation methods might be too unspecific for dealing with missing information for this exploratory trial.

To accommodate for this, for all subjects, who did not complete the WURSS-11 assessments until recovery (e.g. due to presence of symptoms or due to early withdrawal), individual quadratic

regression models will be fitted to available mean daily symptom scores per subject. The estimated symptom day of common cold recovery will then be determined as the symptom day, when the estimated regression line will cross zero.

WURSS-11

For the presentation by symptom days, missing assessments after common cold recovery will be imputed following the Last-observation-carried forward (LOCF) principle. This means, that all values will be set to zero, once recovery has been achieved.

No further imputations will be performed on the single item scale.

For missing mean daily total score at a specific symptom day not related to common cold recovery, missing values will be imputed up to the estimated duration of common cold (but maximum until symptom day 17) based on the individual regression line as defined above.

The AUC-WURSS will then be derived based on imputed values.

A similar approach will be followed for the mean daily group scores.

To investigate the robustness of the results with respect to missing values, pre-planned models will be repeated without imputed values for the mITT.

No imputations are foreseen for other endpoints.

13.11 Sample size estimation

Due to the exploratory character of the study aiming to investigate complete common cold episodes including the very early phase under real-life conditions only sparse information is available. As a consequence the clinical trial is designed based on precision of confidence intervals for the total WURSS-11 score rather than on power considerations based on statistical testing.

Expecting a balanced distribution of subjects into the 3 time periods of time-to-treatment strata, with 110 subjects the 95% confidence intervals of a width of estimated mean ± 2 points could be estimated assuming a total WURSS-11 symptom score of 15 at symptom severity peak based on published common cold trial results. So estimated total WURSS-11 symptom scores at symptom severity peak will have a precision of 4 score points, which is considered as a substantial precision for clinical interpretation of key results.

Assuming that only 66% - 85.5% of screened subjects will experience a cold episode fulfilling all eligibility criteria within a screening phase of up to 7 months, about 400-500 patients are planned to be included into the screening to achieve 330 treated subjects eligible for the analysis (mITT).

14 PERFORMANCE ACCORDING TO GCP

14.1 Declaration of Helsinki / Ethical Guidelines

This clinical trial will be conducted in accordance with the Declaration of Helsinki on Ethical Principles for Medical Research Involving Human Subjects adopted by the General Assembly of the World Medical Association [9].

The planning and conduct of this clinical trial follows the respective national laws of the participating country (Germany), the principles and guidelines for good clinical practice (GCP) laid down in Directives 2001/20/EC [6] and 2005/28/EC [7] of the European Parliament and the International Conference on Harmonization (ICH) Guideline for Good Clinical Practice E6 (R2) [2], and Pharmalog SOPs that are based on the ICH-GCP guidelines.

14.2 Adherence to the protocol

The investigator ensures that the clinical trial is conducted in compliance with this protocol by signing it. Alterations (i.e. amendments) are to be implemented only after approval by EC(s) / IRB(s) and competent authority (for details refer to Section 15.2).

14.3 Summary of Product Characteristics

The investigators have to be supplied with information concerning the pharmacological, toxicological and clinical properties of the substances used within the clinical trial medication in the form of adequate documentation. Since the IMP used in this clinical trial is an authorized product for this indication, the SmPC (Summary of Product Characteristics) will be provided.

14.4 Data protection

All local legal requirements regarding data protection (i.e. Datenschutzgrundverordnung [DSGVO], English translation: General Date Protection Regulation [GDPR]) will be adhered to.

All drug-related information obtained by the investigator and supplied by the sponsor is to be treated as confidential by the investigator and all other personnel involved in the clinical trial. Third persons who are not participating in or working on this clinical trial are not authorized to receive any clinical trial data or material.

The anonymity of participating subjects must be maintained and clinical trial findings will be treated confidentially. Throughout documentation and evaluation, the subjects will be identified on eCRFs and other documents (patient chart, Subject Identification Code List) by their year of birth, and their screening number (pseudonymous data recording). The subjects will be informed that all clinical trial data will be stored on a computer and handled in the strictest confidence. The data will be passed on (e.g. to the sponsor) pseudonymously. In case of publication the data are anonymized.

14.5 Subject informed consent

At Visit 1, prior to subject's participation in the clinical trial, written informed consent will be obtained from each subject according to the Guideline for Good Clinical Practice E6 (R2) [2], the ethical principles that have their origin in the Declaration of Helsinki [9] and the regulatory and legal requirements of the participating country (Germany).

It is the responsibility of the investigator to ensure that the written information form and written consent form and any other written information to be provided to subjects is reviewed by and received favorable opinion from the IRB / EC.

Should a protocol amendment be made, the written information / consent form may need to be revised to reflect the changes to the protocol. Any revised written informed consent form and written information should receive the IRB's / EC's favorable opinion in advance of use. It is the responsibility of the investigator to ensure that an amended consent form is signed by all subjects subsequently screened for trial participation and those currently in the clinical trial, if affected by the amendment.

14.5.1 Subject information

The investigator should fully inform the subject of all pertinent aspects of the clinical trial prior to his entry into the clinical trial. The oral and written information will be given in the local language (German) and should be as non-technical as practicable.

The subject will be informed in an interview with the investigator or a medical member of the investigating team in detail about all aspects of the clinical trial.

The investigator has to inform the subject also about each piece of information arising during the trial period which is important for the subject.

Each subject will have ample time and opportunity to inquire about details of the trial and to decide whether or not to participate in the clinical trial.

14.5.2 Subject informed consent

Two originals of the written informed consent form must be signed and personally dated by the subject and by the investigator who conducted the informed consent discussion.

One original signed written informed consent form and copies of the written information, and the insurance certificate and conditions must be given to the subject.

The other original signed informed consent form will be kept and archived by the investigator in the ISF. The terms of the consent and the date when it was obtained has to be entered in the patient chart and transcribed into the eCRF.

Subjects who withdraw informed consent may not continue in the clinical trial (premature discontinuation).

14.6 Information of other physicians

It is recommended that the investigator informs the subject's primary physician about the subject's participation in the clinical trial if the subject has a primary physician and if the subject agrees to the primary physician being informed.

14.7 Financial or other interests of the investigator

Possible financial or other interests have to be disclosed by the investigator on a Financial Disclosure Form.

15 LEGAL AND ETHICAL ASPECTS

15.1 Notification to authorities and ethics committees

15.1.1 Commencement of the clinical trial

Before commencing the clinical trial in any clinical trial center, Pharmalog will submit on behalf of the sponsor a valid request for authorization to the CA in Germany including this protocol and a summary of essential pharmacological-toxicological and clinical data.

In parallel, Pharmalog will submit on behalf of the sponsor a valid application to the competent EC(s) / IRBs to give its opinion on the submitted documents.

The clinical trial will not start until the competent EC / IRB has issued a favorable opinion (positive vote) and if the CA in Germany (i.e. Bundesinstitut für Arzneimittel und Medizinprodukte [BfArM]) has not informed the sponsor of any reasons for non-acceptance.

15.1.2 Ending of the clinical trial

Within 90 days after the end of the clinical trial, the sponsor/CRO will notify the CA concerned and the EC(s) / IRB(s) concerned that the clinical trial has ended. If the clinical trial has to be terminated prematurely, the notification period will be reduced to 15 days and the reasons will be explained.

15.2 Protocol amendment

Should any change be required to the approved final protocol, a protocol amendment will be required. The amendment must be signed at least by the project manager of the sponsor and the coordinating investigator, and, if necessary, by the statistician.

<u>Substantial amendment</u>

If this amendment is substantial and is likely to have an impact on the safety of the clinical trial subjects or to change the interpretation of the scientific documents in support of the conduct of the clinical trial, or if it is otherwise significant, the sponsor will notify the CA and EC(s) / IRB(s) in Germany of the reasons for, and content of, this amendment.

Should the opinion of the EC(s) / IRB(s) be favorable and the CA in Germany has raised no reasons for non-acceptance of the above-mentioned substantial amendment, the sponsor will proceed to conduct the clinical trial following the amended protocol.

If the opinion of the EC(s) / IRB(s) is unfavorable, the sponsor will not implement this substantial amendment to the protocol. The sponsor will either take account of the reasons for non-acceptance and adapt the proposed amendment to the protocol accordingly or withdraw the proposed amendment.

<u>Non-substantial amendments</u>

Protocol amendments only for logistical or administrative changes may be implemented immediately; the EC(s) / IRB(s) and CA will be informed by the sponsor accordingly.

Urgent safety measures

If any new event relating to the conduct of the clinical trial or the IMP occurs that is likely to affect the safety of the subject, the sponsor and the investigator will take appropriate urgent safety measures to protect the subject against any immediate hazard. The sponsor will inform the CA of this new event and the measures taken immediately and will ensure that the EC(s) / IRB(s) are notified at the same time.

15.3 Inspection

To verify compliance with the provisions on good clinical practice (GCP) and good manufacturing practice (GMP), inspectors (appointed by the CA) may inspect the sites concerned by this clinical trial (sponsor's or CRO's site, clinical trial center(s), manufacturing site, in accordance with Directive 2001/20/EC and the national law or regulations of Germany.

15.4 Direct access to source data / documents

The CRO – on behalf of the sponsor - should ensure that it is specified in investigator agreement that the investigator(s) / institution(s) will permit clinical trial-related monitoring, audits, EC(s) / IRB(s) review, and regulatory inspection(s), providing direct access to source data / documents.

15.5 Subject insurance

The sponsor confirms that an insurance covering the costs of treatment of clinical trial subjects in the event of clinical trial-related injuries in accordance with the local laws of the involved country has been arranged.

Subjects participating in the trial will be insured against injury caused by the clinical trial procedures.

A copy of the insurance policy(ies) and provisions is filed in the Trial Master File (TMF).

15.6 Contracts, finances

All aspects concerning responsibilities and financial affairs are fixed in separate contracts. The sponsor is financier of this clinical trial.

16 CLINICAL TRIAL RESULTS

16.1 Final report

After data analyses, the clinical trial results will be summarized in an integrated (statistical / medical) report which accurately reflects the clinical data of this clinical trial.

The statistical analysis and the final report according to ICH-GCP guidelines will be performed by Pharmalog and will be signed and approved by the coordinating investigator and all other responsible persons.

All information included in the final clinical trial report will be treated as strictly confidential.

16.2 Publication

By signing the clinical trial protocol, the investigator agrees with the use of results of the clinical trial for the purposes of national and international registration, publication and information for medical and pharmaceutical professionals. If necessary, the authorities will be notified of the investigator’s name, address, qualifications and extent of involvement.

The results of this clinical trial may be published in a scientific journal or presented at a scientific meeting.

Publication of clinical trial results requires the consent of the sponsor. It is generally preferable for the results of a multicenter clinical trial to be published as a whole. Details will be defined in the investigator agreement. Any publication of the clinical trial data by the sponsor or investigator(s) will be wholly consistent with the integrated report in accordance with the ethical principles of the Declaration of Helsinki.

17 TIME SCHEDULE OF THE CLINICAL TRIAL

The duration of the entire clinical trial for each subject will be maximal 8 months. The clinical trial will be closed when all subjects under treatment with IMP will have completed the planned clinical trial period. Trial start is defined as first subject first visit (FSFV) and trial end as last subject under treatment with IMP last visit.

Start of clinical part (FSFV):	September / October 2020
Last subject under treatment with IMP last visit:	End of March / April 2021
Database lock:	May / June 2021
Final trial report:	3rd / 4th quarter 2021

18 LITERATURE

[1] **EMA – European Medicines Agency:** ICH Topic E2A, Step 5. Note for Guidance on Clinical Safety Data Management: Definitions and Standards for Expedited Reporting (CPMP/ICH/377/95). Final approval by CPMP: November 1994 / Date for coming into operation: June 1995. Available from: https://www.ema.europa.eu/en/ich-e2a-clinical-safety-data-management-definitions-standards-expedited-reporting.

[2] **EMA – European Medicines Agency:** ICH Topic E6 (R2), Step 5. Note for Guidance on Good Clinical Practice (CPMP/ICH/135/95). Final approval by CPMP: 15 December 2016 / Date for coming into operation: 14 June 2017. Available from: https://www.ema.europa.eu/en/ich-e6-r2-good-clinical-practice.

[3] **EMA – European Medicines Agency:** ICH Topic E9, Note for Guidance on Statistical Principles for Clinical Trials (CPMP/ICH/363/96). Final approval by CPMP: March 1998 / Date for coming into operation: September 1998. Available from: https://www.ema.europa.eu/en/ich-e9-statistical-principles-clinical-trials.

[4] **European Commission:** Communication from the Commission - Detailed guidance on the collection, verification and presentation of adverse reaction reports arising from clinical trials on medicinal products for human use ('CT-3'). Information from European Union Institutions, Bodies, Offices and Agencies – European Commission. (2011/C 172/01). Official Journal of the European Union; 11.6.2011. Available from: https://ec.europa.eu/health/sites/health/files/files/eudralex/vol-10/2011_c172_01/2011_c172_01_en.pdf.

[5] **European Commission:** EudraLex The Rules Governing Medicinal Products in the European Union - Volume 4 EU Guidelines to Good Manufacturing Practice (GMP), Medicinal Products for Human and Veterinary Use: Annex 13 Investigational Medicinal Products. Revision 1 - Brussels, 03 February 2010 (ENTR/F/2/AM/an D (2010) 3374. Adopted by the European Commission: 31 January 2010. Deadline for coming into operation 31 July 2010. Available from: https://ec.europa.eu/health/sites/health/files/files/eudralex/vol-4/2009_06_annex13.pdf.

[6] **European Parliament and the Council of the European Union:** Directive 2001/20/EC of the European Parliament and of the Council of 4 April 2001 on the approximation of the laws, regulations and administrative provisions of the Member States relating to the implementation of good clinical practice in the conduct of clinical trials on medicinal products for human use. Official Journal of the European Communities (OJ L 121, 1.5.2001, p. 34); Available from: https://ec.europa.eu/health/sites/health/files/files/eudralex/vol-1/dir_2001_20/dir_2001_20_en.pdf.

[7] **The Commission of the European Communities:** Commission Directive 2005/28/EC of 8 April 2005 laying down principles and detailed guidelines for good clinical practice as regards investigational medical products for human use, as well as the requirements for authorisation of the manufacturing or importation of such products. Official Journal of the European Union, 9.4.2005; L91, 13-18. Available from: https://ec.europa.eu/health/sites/health/files/files/eudralex/vol-1/dir_2005_28/dir_2005_28_en.pdf.

[8] **Verordnung über die Anwendung der Guten Klinischen Praxis bei der Durchführung von klinischen Prüfungen mit Arzneimitteln zur Anwendung am Menschen (GCP-Verordnung - GCP-V)** vom 9. August 2004 (BGBl. I S. 2081), zuletzt geändert durch Artikel 8 des Gesetzes vom 19. Oktober 2012 (BGBl. I S. 2192). Available from: https://www.gesetze-im-internet.de/gcp-v/BJNR208100004.html.

[9] **World Medical Association (WMA):** WMA Declaration of Helsinki - Ethical principles for medicinal research involving human patients. Adopted by the 18th WMA General Assembly, Helsinki, Finland, June 1964 and lastly amended by the 64th WMA General Assembly, Fortaleza, Brazil, October 2013. - www.wma.net/policies-post/wma-declaration-of-helsinki-ethical-principles-for-medical-research-involving-human-subjects/.

[10] **Aoki FY et al.**: Early administration of oral oseltamivir increases the benefits of influenza treatment. Journal of Antimicrobial Chemotherapy (2003) 51, 123–129 DOI: 10.1093/jac/dkg007.

[11] **Barret B et al.**: The Wisconsin Upper Respiratory Symptom Survey (WURSS). J Fam Pract. 2002 Mar;51(3):265.

[12] **Barret B et al.**: Echinacea for treating the common cold: A randomized controlled trial. Ann Intern Med. 2010 December 20; 153(12): 769–777.

[13] **Barret B:** WURSS-11© (Wisconsin Upper Respiratory Symptom Survey) 2013. https://www.fammed.wisc.edu/files/webfm-uploads/documents/research/wurss-11.pdf. Visited on 30.01.2020.

[14] **Cassella-med GmbH & Co. KG:** Fachinformation Soledum Kapseln forte. August 2019

[15] **Gwaltney JM et al.**: Symptom Severity Patterns in Experimental Common Colds and Their Usefulness in Timing Onset of Illness in Natural Colds. Clinical Infectious Diseases 2003; 36:714–23.

[16] **Jackson GG et al.:** Transmission of the Common Cold to Volunteers Under Controlled Conditions. A. M. A. ARCHIVES OF INTERNAL MEDICINE. 267-278

[17] **Kardos P et al.**: Assessment of the Effect of Pharmacotherapy in Common Cold / Acute Bronchitis - the Bronchitis Severity Scale (BSS). Pneumologie 2014; 68: 542–546.

[18] **Lizogub VG et al.**: Efficacy of a Pelargonium Sidoides Preparation in Patients With the Common Cold: A Randomized, Double Blind, Placebo-Controlled Clinical Trial. EXPLORE November/December 2007, Vol. 3, No. 6. doi:10.1016/j.explore.2007.09.004.

[19] **Obasi CN et al.**: Item reduction of the Wisconsin Upper Respiratory Symptom Survey (WURSS-21) leads to the WURSS-11. Qual Life Res DOI 10.1007/s11136-013-0561-z.

[20] **Robert Koch Institut**: Erhebung, Quantifizierung und Analyse der Rauchexposition in epidemiologischen Studien https://www.rki.de/DE/Content/Gesundheitsmonitoring/Themen/Rauchen/Rauchexposition_epidstudien.pdf?__blob=publicationFile visited on 29.01.2020.

19 SUPPLEMENT

Not applicable
